# Comparing Metabolomics Profiles in Various Types of Liquid Biopsies among Screening Participants with and without Advanced Colorectal Neoplasms

**DOI:** 10.3390/diagnostics11030561

**Published:** 2021-03-20

**Authors:** Vanessa Erben, Gernot Poschet, Petra Schrotz-King, Hermann Brenner

**Affiliations:** 1 Division of Preventive Oncology, German Cancer Research Center (DKFZ) and National Center for Tumor Diseases (NCT), 69120 Heidelberg, Germany; vanessa.erben@nct-heidelberg.de (V.E.); petra.schrotz-king@nct-heidelberg.de (P.S.-K.); 2 Medical Faculty Heidelberg, Heidelberg University, 69120 Heidelberg, Germany; 3 Centre for Organismal Studies (COS), Heidelberg University, 69120 Heidelberg, Germany; gernot.poschet@cos.uni-heidelberg.de; 4 Division of Clinical Epidemiology and Aging Research, German Cancer Research Center (DKFZ), 69120 Heidelberg, Germany; 5 German Cancer Consortium (DKTK), 69120 Heidelberg, Germany

**Keywords:** metabolomics, liquid biopsy, colorectal neoplasms, urine, feces, plasma

## Abstract

Analysis of metabolomics has been suggested as a promising approach for early detection of colorectal cancer and advanced adenomas. We investigated and compared the metabolomics profile in blood, stool, and urine samples of screening colonoscopy participants and aimed to evaluate differences in metabolite concentrations between people with advanced colorectal neoplasms and those without neoplasms. Various types of bio-samples (plasma, feces, and urine) from 400 participants of screening colonoscopy were investigated using the MxP^®^ Quant 500 kit (Biocrates, Innsbruck, Austria). We detected a broad range of metabolites in blood, stool, and urine samples (504, 331, and 131, respectively). Significant correlations were found between concentrations in blood and stool, blood and urine, and stool and urine for 93, 154, and 102 metabolites, of which 68 (73%), 126 (82%), and 39 (38%) were positive correlations. We found significant differences between participants with and without advanced colorectal neoplasms for concentrations of 123, 49, and 28 metabolites in blood, stool and urine samples, respectively. We detected mostly positive correlations between metabolite concentrations in blood samples and urine or stool samples, and mostly negative correlations between urine and stool samples. Differences between subjects with and without advanced colorectal neoplasms were found for metabolite concentrations in each of the three bio-fluids.

## 1. Introduction

Colorectal cancer (CRC) is the third most common cancer type worldwide [1]. It develops over a long period of time through the adenoma-carcinoma sequence in most cases [2]. Metabolic changes occur early during the course of carcinogenesis [3]. Many of the dysregulated metabolites can be linked to CRC but it is still not clear to what extent metabolic perturbation or metabolic alterations are causes, indicators of causes or consequences of tumor development [4].

As cancer is a very heterogeneous disease it seems to be clear that one altered metabolic pathway might not be sufficient for characterization of metabolic changes associated with tumorigenesis [5]. For the assessment of metabolic changes in early detection of adenomas investigation of tissue samples or tissue biopsies is not the method of choice because of the invasiveness of such an approach. In addition, early cancer stages might be neither easily detected nor accessible for biopsies.

For cancer prevention and early detection, there is a strong need for non-invasive technologies and easily accessible body-fluids, so called liquid biopsies. Metabolomics are closely related to the phenotype and therefore of great interest to and in focus of many researchers investigating biomarkers or biomarker signatures for early detection of CRC [6,7,8] or advanced adenomas [9,10,11,12]. So far, most research groups have investigated metabolomics changes only in one body-fluid. However, changes of metabolic pathways favoring carcinogenesis might go along with changes in metabolomics markers not only in one but in several body-fluids.

To our knowledge, no study so far has investigated and compared the metabolic profile of people with and without advanced colorectal neoplasms in three different bio-fluids (plasma, stool, and urine). In this study, we used the MxP^®^ Quant 500 Kit (Biocrates Life Sciences AG, Innsbruck, Austria) to measure, in parallel, a broad spectrum of metabolites in human blood, urine and fecal samples of participants of screening colonoscopy. We thereby aimed for a comprehensive comparison of metabolic profiles between the various bio-fluids, and their relationship with questionnaire data and findings at screening colonoscopy. Objectives of this study were to assess similarities as well as differences in the metabolic profile of individuals with and without colorectal neoplasms and possible correlations of the metabolites in the different bio-fluids.

## 2. Materials and Methods

### 2.1. Study Design and Population

Participants of screening colonoscopy were recruited in the GEKKO study (Gebt dem Krebs keine Chance—Onkocheck). In this ongoing multi-center study we aimed to evaluate novel noninvasive cancer early detection markers, participants are recruited at a pre-colonoscopy visit to gastroenterological practices in South-West Germany. Participants 30 years or older, speaking and understanding the German language with no previous colonoscopy in the last 5 years, no personal history of CRC, and no inflammatory bowel disease are eligible to participate. The study was approved by the ethics committees of the Medical Faculty Heidelberg and of the physicians’ boards of Baden-Württemberg and Rhineland Palatinate. The study was performed in accordance with the Declaration of Helsinki. Written informed consent is received from all participants.

Upon receipt of written informed consent, participants are asked to fill in a questionnaire regarding lifestyle and demographic data and to provide blood, stool, saliva, and urine samples for biomarker analyses prior to colonoscopy. Findings at colonoscopy are abstracted from colonoscopy and histology reports independently by two trained investigators who are blinded with respect to questionnaire data and results of biomarker analyses. Participants are classified according to the most advanced finding at colonoscopy: CRC, advanced adenoma (defined by either adenoma >1 cm in size or tubulovillous or villous components or high-grade dysplasia), non-advanced adenoma, hyperplastic polyp, or none of these findings [13].

**Figure 1:**
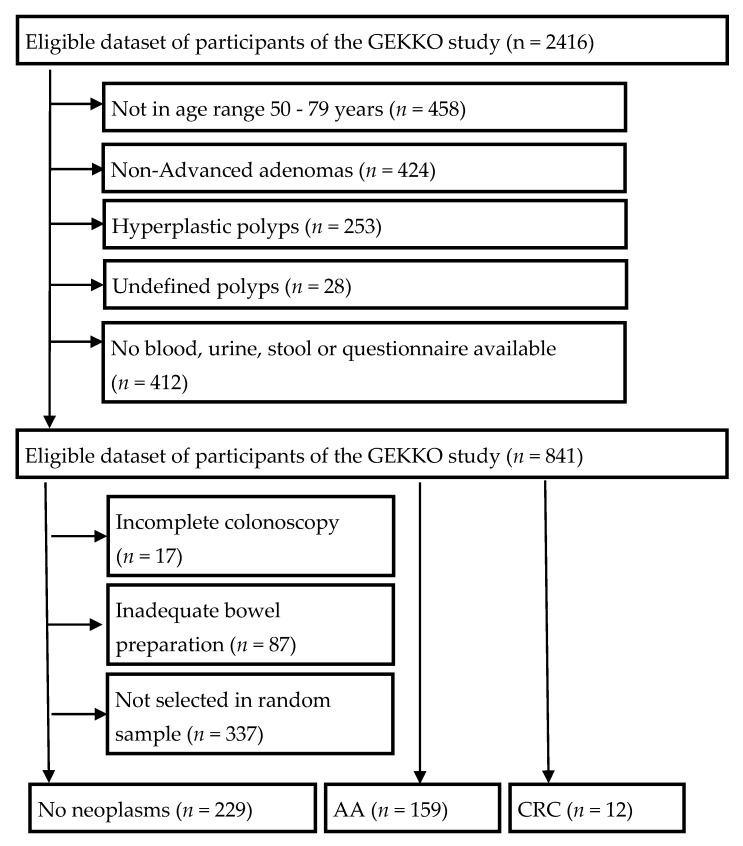
Flow diagram with inclusion and exclusion criteria.

Among 2416 GEKKO participants recruited between January 2016 and August 2019, a total of 400 participants aged 50–79 years were selected as outlined in Figure 1. We selected all eligible participants with either advanced adenoma (*n* = 159) or CRC (*n* = 12), as well as a random sample of 229 participants free of neoplasms or hyperplastic polyps for whom all three types of biospecimen (blood, stool, and urine) as well as questionnaire data were available. For the control group free of neoplasms or hyperplastic polyps, incomplete colonoscopy and inadequate bowel preparation were additional exclusion criteria in order to minimize the risk of false negative colonoscopy results.

### 2.2. Sample Collection and Handling

All bio-samples were collected prior to colonoscopy. Blood and urine samples were processed within 4 h according to standard operating procedures (SOPs) and immediately stored at −80 °C.

Native stool samples were collected by the participants at home from a normal bowel movement prior to bowel preparation for colonoscopy with standard stool collection tubes including a small spoon. The stool samples were frozen by the participants at −20 °C at home. Participants were asked to document the time of sampling and the storage temperature. The stool samples were taken by the participant in a freeze-cool transport container and in an isolated envelope to the gastroenterologists’ practices, where they were immediately frozen again at −20 °C. The samples were delivered within the week of receipt by a transport service on dry ice to the GEKKO study laboratory at the National Center for Tumor Diseases (NCT) in Heidelberg where they were frozen at −80 °C.

### 2.3. Processing of the Samples

In total, 630 metabolites covering 14 small molecule and 12 different lipid classes were analyzed using the MxP^®^ Quant 500 kit (Biocrates Life Sciences AG, Innsbruck, Austria) following the manufacturer’s protocol.

In brief, 10 µL human plasma were pipetted on a 96-well-plate containing internal standards, dried under a nitrogen stream using a positive pressure manifold (Waters, Milford, MA, USA) and 50 µL 5% phenyl isothiocyanate (PITC) solutionwere added to each well to derivatize amino acids and biogenic amines. After 1h incubation time at room temperature, the plate was dried again. To extract the metabolites 300 µL 5 mM ammonium acetate in methanol were pipetted to each filter and incubated for 30 min. The extract was eluted into a new 96-well-plate using positive pressure. For further LC-MS/MS analyses 150 µL of the extract was diluted with an equal volume of water. For FIA-MS/MS analyses 10 µL extract was diluted with 490 µL FIA solvent (provided by Biocrates, Innsbruck, Austria). After dilution, LC-MS/MS and FIA-MS/MS measurements were performed. For chromatographical separation an UPLC I-class PLUS (Waters, Milford, MA, USA) system was used coupled to a SCIEX QTRAP 6500+ mass spectrometry system in electrospray ionization (ESI) mode. Data was generated using the Analyst (Sciex) software suite and transferred to the MetIDQ software (version Oxygen; Biocrates Life Sciences AG, Innsbruck, Austria) which was used for further data processing and analysis. All metabolites were identified using isotopically-labeled internal standards and multiple reaction monitoring (MRM) using optimized MS conditions as provided by Biocrates (Innsbruck, Austria). For quantification either a seven point calibration curve or one point calibration was used depending on the metabolite class.

Urine samples were processed similar to the blood samples with no prior preparation. Additionally, in every well (except for the blank) an internal standard (creatinine) was added before urine or the standards were pipetted onto the plate. Metabolite concentrations were normalized to the creatinine content.

In a pilot study including 3 stool samples from different people, 8 different protocols for sample preparation were evaluated. The protocol with a stable high number of detected metabolites was finally chosen for the 400 stool samples of the GEKKO study. In brief, 50 mg native stool samples were mixed with 200 µL iced ethanol (75%) and vortexed for 2 min. The mixture was then sonicated in an ultrasonic bath on ice-cooled water. Afterwards, 500 µL methyl tert-butyl ether (MTBE) were added and the mixture was shaken at room temperature for 1 h (800–900 rpm). For phase separation, 125 µL water was added to the mixture, vortexed for 2 min and incubated for 10 min at room temperature. The mixture was centrifuged for 15 min at 4 °C at full speed (21,000× *g*) and the supernatant (both phases) then transferred to another tube. The supernatant was then completely dried in a vacuum concentrator (SpeedVac, Concentrator plus, Eppendorf, Hamburg, Germany) without any temperature manipulation (max. 30 °C) and stored at −80 °C until measurement with the Mxp^®^ Quant 500 kit (Biocrates, Innsbruck, Austria). The dried samples had to be resolved before measurements. Therefore, 50 µL 100% isopropanol were added into the vial and the mixture was vortexed for 3 min at room temperature. Additionally, 50 µL 30% isopropanol were added and again vortexed for 3 min at room temperature. A short centrifugation (5 sec) separated the solid substances from the liquid phase which was used for further analysis. Data were normalized with a tissue factor assuming that 1 mg tissue equals 1 µL tissue or stool.

### 2.4. Statistical Analyses

Demographic characteristics of the study population were described. A dietary quality score and a healthy lifestyle score reflecting smoking status, alcohol intake, diet, physical activity, and BMI was calculated from questionnaire data as previously described [14,15] and outlined in Table S1. Differences between participants with and without advanced neoplasms were tested for statistical significance using chi-square test (categorical variables) or Mann–Whitney U test (continuous variables).

Metabolite concentrations in urine, blood, and stool were calculated. We investigated the number and classes of metabolites in blood, urine, and stool samples for which the mean concentrations of the metabolite were above the limit of detection (LOD) among participants without advanced neoplasms. We defined those metabolites as detectable in the respective sample type and assessed how many metabolites were detectable in more than one of the tested bio-fluids.

Moreover, we assessed the correlation of the metabolites between the different bio-fluids using Spearman rank correlation coefficients, both for the total study population as well as separately for the subgroups of participants with and without advanced neoplasms.

Differences in metabolite concentrations in each type of biospecimen between participants with and without advanced colorectal neoplasms were evaluated for statistical significance using Mann–Whitney *U* test.

A *p*-value < 0.05 (two-sided testing) was considered to indicate statistical significance in any of the analyses. Analyses were conducted with SAS Enterprise Guide 7.1 (SAS Institute Inc., Cary, NC, USA).

## 3. Results

### 3.1. Study Population

The selection of the 171 and 229 study participants with and without advanced colorectal neoplasms is shown in Figure 1. Only those participants with available questionnaire data, plasma, urine and native stool samples were included.

Among the participants with advanced colorectal neoplasms, 60% were male and mean age was 64.1 years and among those with no finding at colonoscopy 54% were male and mean age was 60.9 years (Table 1.). The majority of the participants were never smokers. More participants with advanced neoplasms (19%) were current smokers compared to participants with no finding at colonoscopy (10%). Study participants with no finding at colonoscopy had a more favorable lifestyle compared to those with advanced colorectal neoplasms.

### 3.2. Metabolite Profiles in Various Human Bio-Samples

**Figure 2:**
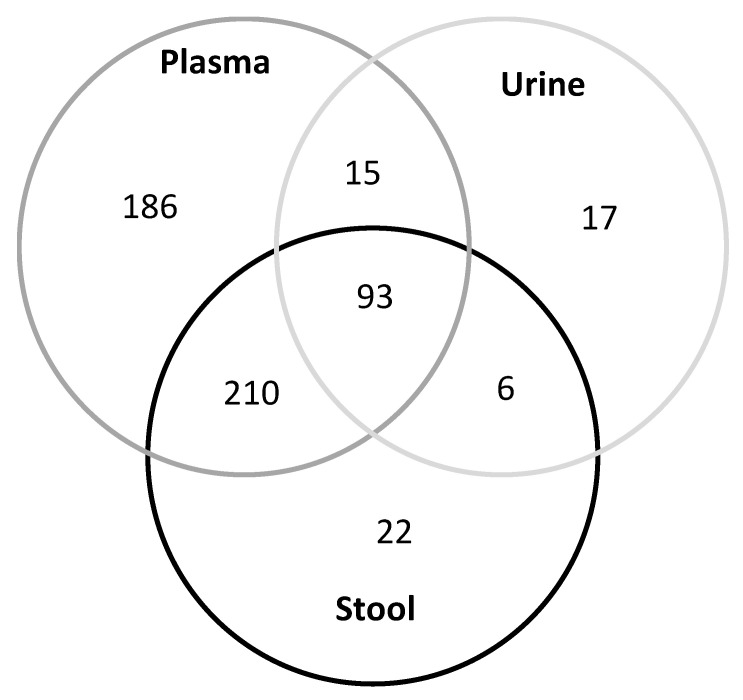
Venn diagram of metabolites measurable in plasma, feces, and urine.

We were able to detect a broad range of different metabolites in each bio-fluid. The metabolites with the mean greater than the LOD among the participants without advanced neoplasms were regarded as present in the specific bio-fluid (before normalization to the tissue factor in stool and to creatinine in urine). We detected 504 metabolites in plasma, 331 in stool and 131 in urinary samples (Table S2). Amino acids were present in all the investigated biosamples. In total, 93 metabolites were present in all three bio-fluids (many amino acids and amino acid related metabolites), 210 were present in plasma and stool only, 15 in plasma and urine only and 6 in stool and urine only (Figure 2.). Some other metabolites were only present in one of the investigated bio-fluids (186 in plasma, 22 in stool and 17 in urine). For 81 metabolites the mean concentrations were below the LOD in any of the bio-fluids, such as some of the acylcarnitines, diacylglycerols, or nitro-tyrosine.

### 3.3. Correlation of Metabolites in Liquid Biopsies

**Figure 3:**
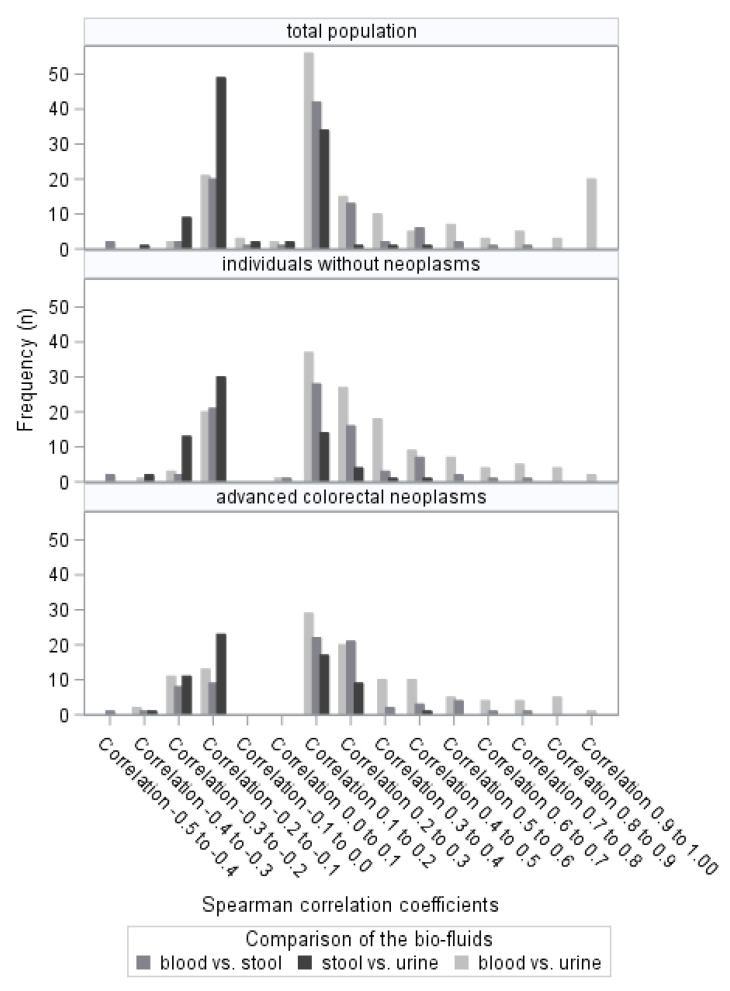
Distribution of the Spearman correlation coefficients (only significant correlations are displayed).

We calculated the Spearman rank correlation coefficient to assess correlations of the metabolites in the respective bio-fluids (Table 2). We found fecal and urinary metabolites to be more frequently negatively correlated, whereas urinary and blood as well as stool and blood metabolites were more frequently positively correlated. A total of 68 metabolites were significantly positively correlated between blood and stool samples, 126 metabolites were positively correlated between blood and urine samples, and 63 metabolites were negatively correlated between stool and urine samples. A similar picture was seen for the subgroups of individuals with and without advanced colorectal neoplasms (Figure 3). We assessed for the total study population the chemical subclasses of the metabolites that were significantly correlated. Most positive correlations were seen in the blood vs. stool comparisons for acylcarnitines and amino acid related metabolites and in the blood vs. urine comparisons for amino acids and amino acid related metabolites as well as bile acids, acylcarnitines and glycerophospholipids. Most negative correlations for the stool vs. urine comparisons were seen for acylcarnitines, cholesteryl esters and triglycerides. 

### 3.4. Differences in Metabolite Concentrations between Participants with and without Advanced Colorectal Neoplasms

We found significant differences in metabolite concentration in the different bio-fluids when participants with advanced colorectal neoplasms were compared to those without any finding at colonoscopy (Table S3). No metabolite showed significantly different levels in all investigated bio-fluids. We found 133, 98, and 80 metabolites in plasma, stool and urine, respectively, which were significantly different between participants with advanced colorectal neoplasms and individuals without any finding at colonoscopy. Most prominent changes were diacylglycerols and triacalglycerols in stool samples, glycerophospholipids, and nucleobase related metabolites in blood and amino acids (especially Ala) and hexoses in urine samples.

## 4. Discussion

A variety of metabolites could be measured in human different human bio-fluids (blood, stool, and urine) from participants of screening colonoscopy with the MxP^®^ Quant 500 kit (Biocrates, Innsbruck, Austria). Metabolite concentrations vary between different human bio-fluids and between study participants with advanced colorectal neoplasms and without neoplasms or hyperplastic polyps. We found predominantly positive correlations when comparing blood and urine as well as blood and stool metabolite concentrations and predominantly negative correlations for stool vs. urine comparisons.

Metabolomics studies on different samples types or different liquid biopsies in early detection of CRC are sparse. A study from Lin et al. investigated CRC tissue and fecal samples and found different metabolic changes between CRC tissues and corresponding fecal samples. The fecal metabolite profile might reflect the tumor microenvironment in the gut [16]. Another study from the USA had similar findings and showed overlapping but as well a range of distinct metabolites from CRC tissue and feces concluding that these metabolites are not directly associated [17]. Similarly, studies on various cancer types did not find clear associations of the cell metabolome with metabolomics findings in urine or blood samples [18]. A German study investigating adipose tissue and blood samples from CRC patients found only low correlations between serum and adipose tissue metabolites, however moderate correlations for triglycerides of adipose tissue and sphingomyelins of serum were detected [19]. To our knowledge, ours is the first study to directly compare different liquid biopsies taken from the same participant at one timepoint and to get a broad look on the metabolic profile in different human bio-fluids within and amongst participants of a study conducted in a real life screening setting.

The approach of combining the analysis of different bio-fluids for metabolomics research in order to provide a broad look at the metabolic profile specific for a disease has already been proposed a few years ago [20], but, to our knowledge, has not been performed systematically so far. The combination of different bio-fluids or other -omics approaches might improve diagnostic performance and should come into focus in future research.

Additionally, we found significant differences in metabolite concentrations between the three bio-fluids. A look at the role of each of these fluids in the human body might explain some of the observed differences. Blood passes every part in the body, is transport medium of various molecules, and metabolite concentrations are tightly regulated while giving important information on the physiological status [18]. In contrary, urine and stool are excreted from the body, yet they cannot be regarded as simple “waste products” as it was done earlier. Urine contains a lot of water-soluble and metabolic by-products which can be used for diagnostic or prognostic purposes [21,22]. In human feces small compounds and metabolites can be found in the dry mass that can be used for metabolomics studies. Stool may directly reflect the tumor microenvironment through its transit in the gut and the direct contact to the tumor and might therefore be a potential source for biomarkers for early detection [23]. A study investigating the metabolome of stool and tissue found that a biomarker combination for stool and as well for tissue was able to differentiate between CRC cases and healthy controls. The authors concluded from the overlapping markers metabolic pathways perturbations that are characteristic for CRC such as glucose and glycolytic activity, tricarboxylic acid cycle, glutaminolysis, and metabolism of short chain fatty acids [16]. Another study showed differences of metabolisms of short chain fatty acids and the glycolytic/gluconeogenic pathway when investigating tissue and fecal samples [17].

We found predominantly positive correlations for blood vs. stool and vs. urine and predominantly negative correlations for urine vs. stool. Blood is an extra-cellular fluid that passes every organ in the body and reflects the metabolic phenotype of the organism [18]. Water-soluble compounds are filtered in the kidneys and excreted with the urine. Human feces in contrast contains endogenous and exogenous components and, besides variable amounts of water, solid material from bacteria or undigested food and many other components contributing to the stool metabolome [24]. The predominantly positive correlations between blood and stool and blood and urine concentrations appear plausible given that specific metabolites from blood are either excreted via urine or stool. The predominantly negative correlations between urine and stool concentrations on the other hand may reflect the fact that specific metabolites are either excreted in urine or in stool and might therefore not be present in both body excretions.

Moreover, we detected significant differences in metabolite concentrations between study participants with advanced colorectal neoplasms and individuals with no neoplasms in each of the different bio-fluids. Most significant differences were seen among the metabolite concentrations in blood. There exist already a range of metabolomics studies in different liquid biopsies for CRC detection, diagnosis, and prognosis but, to our knowledge, no study so far has investigated all three bio-fluids (blood, urine, stool) in a prospective screening study. Metabolites are useful for distinguishing CRC cases from people without advanced neoplasms in various bio-fluids but there is no consensus which metabolite or metabolite panels are the most suitable biomarkers [7].

The vast majority of our study participants with advanced colorectal neoplasms had advanced adenomas but no CRC yet. Even for such a case group mostly consisting of carriers of cancer precursors, which represents a major target group of CRC screening, we were able to detect metabolic differences from participants without colorectal neoplasms. One could postulate that not only the tumor itself is responsible for metabolic changes, but that metabolic perturbations might be responsible for carcinogenic growth [25]. This so called-metabolic reprogramming can be caused by inactivation of tumor-suppressor genes or activation of proto-oncogenes as a consequence of mutations [26]. One study has found that this occurs already in the adenoma stage before a tumor is manifested [27]. On the other hand metabolites can introduce oncogenic effects by themselves [28]. Some metabolic changes are advantageous for cell proliferation which is one hallmark of cancer such as providing building blocks [5]. Finally, metabolic changes could simple be markers correlated with cancer promoting dietary risk behaviors without a causal role in oncogenesis in which case they could still be useful as biomarkers for risk stratification of early detection.

Regarding the heterogeneous result of metabolomics studies, one should think about pre-analytics and other influencing factors. On the one hand, there exist no standard operating procedure and uniform protocols for pre-analytics in order to make metabolomics results from different studies comparable [29]. Storage temperature and processing delay can effect metabolite concentration in blood samples [30]. Results of different studies are not conclusive in their findings if metabolites are stable for a specific number of freeze-and-thaw cycles or if freezing and thawing should be avoided whenever possible. Another study found that metabolomics analyses are better reproducible when using fasting blood samples compared to non-fasting samples [31]. Storage time and temperature also have influence on the urinary metabolome and freeze-and-thaw cycles should be avoided with regard to metabolite coverage [32]. In both blood and urine samples, amino acids are one of the most sensitive and least stable classes of metabolites and freezing as soon as possible is recommended [32,33]. To account for different hydration in urine sample, various correction methods are available such as normalization to creatinine as we used it in this analysis [34]. Moreover, stool is very heterogeneous itself and water content can vary. Therefore, there is urgent need for a uniform sample preparation protocol. We have shown in previous analysis that metabolite classes and concentrations are highly dependent on the extraction method used [35]. On the other hand, the metabolome is highly dependent on other factors such as age, lifestyle, diet, or antibiotics use which cannot (easily) be standardized.

Our study has several strengths. To our knowledge, it is the first study investigating three kinds of liquid biopsies (plasma, stool, and urine) from the same study participants for metabolomics biomarkers under controlled (study standard operating procedures) and reproducible conditions (MxP Quant 500 kit, Biocrates, Innsbruck Austria). Furthermore, we included participants of a prospective screening cohort which is more appropriate for biomarker development for early detection than the widely used case-control setting in which biospecimen are taken from CRC patients after diagnosis. Samples were processed according to a standard protocol within 4 h which ensures best possible sample quality. We avoided freeze–thawing cycles by taking wet stool from the frozen total samples but other researchers prefer homogenizing or drying the stool samples before doing metabolomics analysis to decrease variability [36].

This study has also limitations. We used a cross-sectional design and samples were taken at one time point. Despite the overall large size of the screening population from which our study sample was drawn, the number of patients with CRC was rather low (*n* = 12). While the low prevalence of CRC reflects the situation encountered in true screening settings, the small number of CRC cases prohibited meaningful separate analyses for this subgroup of participants with advanced neoplasms. Although we processed samples according to standardized procedures, urinary samples were taken from “spontaneous” urine and blood samples were not taken under fasting conditions. In addition, participants did not get a standardized diet before sample collection which might introduce bias. Not only diet but also consumption of beverages or antibiotics can have an influence on metabolomics.

In conclusion, we have provided a holistic look at the metabolic profile of individuals without neoplasm and those with advanced colorectal neoplasms in a prospective screening cohort. We found a great number of metabolites in all investigated bio-fluids. Metabolites from plasma samples compared with urine or feces were more predominantly positively correlated whereas metabolites from stool compared to urine were predominantly negatively correlated. We found a range of metabolites to be differentially expressed in bio-fluids (plasma, feces, and urine) from participants with advanced colorectal neoplasms and participants without neoplasms. Further research should aim for deriving and validating metabolomic algorithms from various body fluids for risk stratification in CRC screening and development of biomarkers for noninvasive early detection of advanced colorectal neoplasia. The results of our study may provide important background data to inform and design such studies. 

## Figures and Tables

**Table 1:** Characteristics of the study participants.

Characteristics	No Neoplasms	AA/CRC	*p* Value ^1^
	*n* = 229	*n* = 159/12	
Sex, *n* (%)			
Female	106 (46%)	68 (40%)	0.19
Male	123 (54%)	103 (60%)	
Age, *n* (%)			
50–59 years	122 (53%)	61 (36%)	0.0006
60–69 years	65 (28%)	55 (32%)	
70–79 years	42 (18%)	55 (32%)	
Mean, (SD)	60.9 (±8.0)	64.1 (±8.6)	0.0002
Smoking status, *n* (%)			
Current	23 (10%)	32 (19%)	0.0031
Former	79 (34%)	71 (42%)	
Never	127 (55%)	68 (40%)	
BMI (kg/m^2^), mean	26.1 (±4.2)	26.9 (±4.6)	0.06
Alcohol consumption (g/day), mean			
Women	6.1 (±10.2)	8.8 (±34.7)	0.17
Men	9.0 (±12.1)	13.9 (±14.5)	0.007
Leisure time physical activity MET-h/week, mean (SD)	42.7 (±57.6)	37.3 (±41.4)	0.08
Dietary quality score, mean ^2^	31.0 (±6.7)	28.7 (±6.7)	0.0005
Healthy Lifestyle score ^2^			
4 or 5 points	99 (43%)	50 (29%)	0.0005
3 points	96 (41%)	66 (39%)	
0 or 1 or 2 points	34 (15%)	55 (32%)	

Abbreviations: AA, advanced adenomas; BMI, body mass index; CRC, colorectal cancer; MET, metabolic equivalent of task; SD, standard deviance; ^1^ *p*-values were calculated with Chi-square test (categorical variables) or Mann–Whitney U test (continuous variables); ^2^ BMI *n* = 11 are missing, Dietary quality score *n* = 3 are missing. Missing values are ranked 0 points for the Healthy Lifestyle score.

**Table 2:** Spearman Correlation Coefficients between metabolites in different bio-fluids.

	Total	Blood vs. Stool		Blood vs. Urine		Stool vs. Urine	
		Pos. *n* (%)	Neg. *n* (%)	Pos. *n* (%)	Neg. *n* (%)	Pos. *n* (%)	Neg. *n* (%)
Correlation −0.5 to ≤−0.4			1 (0.16)		0		0
Correlation −0.4 to ≤−0.3			1 (0.16)		2 (0.32)		1 (0.16)
Correlation −0.3 to ≤−0.2			8 (1.27)		11 (1.77)		11 (1.77)
Correlation −0.2 to ≤−0.1			38 (6.04)		52 (8.36)		88 (14.13)
Correlation −0.1 to ≤0.0			201 (31.96)		183 (29.42)		266 (42.70)
Correlation 0.0 to ≤0.1		268 (42.61)		233 (37.46)		188 (30.18)	
Correlation 0.1 to ≤0.2		80 (12.72)		82 (13.18)		59 (9.47)	
Correlation 0.2 to ≤0.3		21 (3.34)		20 (3.22)		9 (1.44)	
Correlation 0.3 to ≤0.4		2 (0.32)		10 (1.61)		0	
Correlation 0.4 to ≤0.5		3 (0.48)		10 (1.61)		1 (0.16)	
Correlation 0.5 to ≤0.6		4 (0.64)		5 (0.80)		0	
Correlation 0.6 to ≤0.7		1 (0.16)		4 (0.64)		0	
Correlation 0.7 to ≤0.8		1 (0.16)		4 (0.64)		0	
Correlation 0.8 to ≤0.9		0		5 (0.80)		0	
Correlation 0.9 to ≤1.00		0		1 (0.16)		0	
Significant correlations							
Total study population	630	68	25	126	28	39	63
Participants without neoplasms	630	59	25	114	28	20	49
Participants with advanced colorectal neoplasms	630	54	20	88	34	27	42
Total study population, significant correlations							
Alkaloids	1	1	0	1	0	1	0
Amine Oxides	1	0	0	1	0	1	0
Amino Acids	20	1	0	17	0	5	0
Amino acid related	30	11	1	26	1	10	3
Bile Acids	14	3	1	13	0	2	1
Biogenic Amines	9	1	0	3	0	1	1
Carbohydrates and related	1	0	0	0	0	0	0
Carboxylic Acids	7	1	0	3	0	0	0
Cresols	1	0	0	1	0	0	0
Fatty Acids	12	6	2	1	5	0	2
Hormones and related	4	2	0	4	0	0	0
Indoles and Derivatives	4	2	0	3	0	1	0
Nucleobases and related	2	0	0	2	0	0	0
Vitamins and Cofactors	1	0	0	1	0	0	0
Acylcarnitines	40	17	6	15	8	4	19
Glycerophospholipids (Lysophosphatidylcholines and Phosphatidylcholines)	90	5	3	18	3	1	2
Sphingomyelins	15	0	1	0	1	0	0
Cholesteryl Esters	22	3	1	2	2	0	11
Ceramides	28	5	1	2	1	1	0
Dihydroceramides	8	0	0	1	1	0	1
Glycosylceramides (Mono-, Di-, and Trihexosylceramides)	34	0	0	0	0	1	0
Diglycerides	44	3	5	7	2	0	9
Triglycerides	242	7	4	5	4	11	14

## Data Availability

The data presented in this study are available in this article “Metabolomics profiles in various types of liquid biopsies among screening colonoscopy participants with and without advanced colorectal neoplasms”.

## Supplementary Materials

The following are available online at https://www.mdpi.com/2075-4418/11/3/561/s1 [37,38,39,40,41].
