# Supplementary material for: Comparing Metabolomics Profiles in Various Types of Liquid Biopsies among Screening Participants with and without Advanced Colorectal Neoplasms
Source: Diagnostics (Basel). 2021 Mar 20;11(3):561. doi: 10.3390/diagnostics11030561 (PMC8003917; doi:10.3390/diagnostics11030561)
Supplement: Supplementary file 1 [file diagnostics-11-00561-s001.pdf]

Table S1. Description and prevalence of the lifestyle factors included in the healthy lifestyle score.

Lifestyle Factor	Points	Description	No Neoplasms	ACN
**Smoking** [1]	0	Current smoker & former smoker (≥30 pack years)	23 (10.0)	133 (77.8)
	1	Never smoker & former smoker (<30 pack years)	206 (90.0)	38 (22.2)
**Alcohol intake** [2]	0	Did not meet recommendations on alcoholic drinks	31 (13.5)	29 (17.0)
	1	Met recommendation on alcoholic drinks	198 (86.5)	142 (83.0)
**Diet** [3]	0	Unhealthy diet quality: diet score <34	140 (61.1)	133 (77.8)
	1	Healthy diet quality: diet score ≥34	89 (38.9)	38 (22)
**Physical activity** [4]	0	Did not meet physical activity guidelines	28 (12.2)	39 (22.8)
	1	Met physical activity guidelines	201 (87.8)	132 (77.2)
**BMI** [5]	0	Overweight or obese ≥25kg/m^2	140 (61.1)	114 (66.7)
	1	Healthy weight >18.5 – <25kg/m^2	89 (38.9)	57 (33.3)

[1] Pack years (defined as 20 cigarettes /day in one year) were calculated and never smokers or former smokers with <30 pack years received 1 point in the score [37]; [2] The amount of pure alcohol per week (a standard glass of 0.33L beer, 0.25L wine and 0.02L spirits contains 4, 8.6 and 33 g/100mL of pure ethanol respectively) were calculated. If the recommendations for men (<24 g/day) or women (<12 g/day) of the World Cancer Research Fund/American Institute for Cancer Research (WCRF/AICR) (2007) were met [38], participants got 1 point; [3] Reported average frequency consumption of dietary components (ranging from never to several times per day) were summarized for rating of a healthy diet. Risk factors (red meat, processed meat) were rated negatively, protective factors (whole-grains, fruits, vegetables/salads) were rated positively and an overall healthy diet (≥34 out of a maximum of 50 possible points) was worth 1 point [14,15]; [4] Metabolic equivalents of tasks (METs) for leisure time activity (vigorous and light activity) were calculated [39]. One point was given for fulfilling the physical activity recommendations of at least 500 MET min/week as given by the World Health Organization [40] (The WHO Global Recommendations on Physical Activity for Health (2010) recommend adults to engage in at least 150 minutes of moderate-intensity or 75 min of vigorous-intensity aerobic physical activity throughout the week or an equivalent combination of moderate and vigorous intensity physical activity [40]); [5] Body mass index (BMI) was calculated as a measure of obesity [41]. One point was given for a healthy weight (≥18.5 kg/m^2 - <25 kg/m^2); Abbreviations: ACN, advanced colorectal neoplasm; BMI, body mass index.

Table S2. Metabolites sorted by chemical class and their LODs and mean concentrations for the different bio-fluids among the individuals without colorectal neoplasms; means > LOD are marked in green.

Metabolite	Class	Stool LOD	Stool Mean	Plasma LOD	Plasma Mean	Urine LOD	Urine Mean
C0	Acylcarnitines	1.71	4.38	1.6586	37.79	4.167	31.13
C2	Acylcarnitines	0.2016	0.2	0.3704	5.8	0.262	21.99
C3	Acylcarnitines	0.2526	0.12	0.3134	0.42	0.164	1.23
C3:1	Acylcarnitines	0.1692	0.07	0.0826	0.03	0.103	0.18
C3-DC (C4-OH)	Acylcarnitines	0.203	0.15	0.0298	0.06	0.223	0.09
C3-OH	Acylcarnitines	0.3058	0.12	0.1182	0.04	0.136	0.05
C4	Acylcarnitines	0.1682	0.08	0.0904	0.19	0.119	5.85
C4:1	Acylcarnitines	0.1686	0.06	0.0456	0.02	0.114	0.14
C5	Acylcarnitines	0.6042	0.2	0.3064	0.17	0.277	2.44
C5:1	Acylcarnitines	0.3552	0.09	0.0932	0.04	0.194	0.55
C5:1-DC	Acylcarnitines	0.222	0.07	0.0392	0.01	0.145	0.13
C5-DC (C6-OH)	Acylcarnitines	0.3008	0.12	0.0992	0.05	0.174	0.55
C5-M-DC	Acylcarnitines	0.1706	0.06	0.0306	0.01	0.072	0.14
C5-OH (C3-DC-M)	Acylcarnitines	0.3556	0.17	0.1096	0.05	0.180	1.42
C6 (C4:1-DC)	Acylcarnitines	0.2262	0.11	0.1404	0.11	0.150	0.68
C6:1	Acylcarnitines	0.1466	0.06	0.07	0.04	0.080	0.07
C7-DC	Acylcarnitines	0.1834	0.09	0.153	0.06	0.081	0.16
C8	Acylcarnitines	0.4984	0.21	0.2694	0.2	0.401	0.68
C9	Acylcarnitines	0.1282	0.05	0.0566	0.07	0.076	1.59
C10	Acylcarnitines	0.4704	0.22	0.1714	0.29	0.433	0.43
C10:1	Acylcarnitines	0.2742	0.11	0.33	0.15	0.436	0.8
C10:2	Acylcarnitines	0.4476	0.2	0.588	0.21	0.394	3.25
C12	Acylcarnitines	0.305	0.14	0.1336	0.11	0.264	0.3
C12:1	Acylcarnitines	0.163	0.16	0.161	0.09	0.880	0.29
C12-DC	Acylcarnitines	0.934	0.42	1.2108	0.39	0.947	0.37
C14	Acylcarnitines	0.3198	0.17	0.1044	0.06	0.183	0.1
C14:1	Acylcarnitines	0.0952	0.05	0.0366	0.06	0.064	0.04
C14:1-OH	Acylcarnitines	0.1156	0.04	0.0314	0.01	0.062	0.03
C14:2	Acylcarnitines	0.1556	0.06	0.1442	0.05	0.149	0.06
C14:2-OH	Acylcarnitines	0.0928	0.04	0.0232	0.02	0.058	0.03
C16	Acylcarnitines	0.291	0.23	0.0544	0.13	0.159	0.18
C16:1	Acylcarnitines	0.1134	0.06	0.0434	0.03	0.140	0.05
C16:1-OH	Acylcarnitines	0.0852	0.04	0.0176	0.01	0.053	0.02
C16:2	Acylcarnitines	0.1126	0.06	0.041	0.01	0.095	0.05
C16:2-OH	Acylcarnitines	0.0998	0.05	0.0636	0.02	0.116	0.04
C16-OH	Acylcarnitines	0.141	0.07	0.0418	0.02	0.089	0.08
C18	Acylcarnitines	0.1514	0.17	0.047	0.05	0.087	0.05
C18:1	Acylcarnitines	0.1404	0.13	0.0848	0.14	0.092	0.04
C18:1-OH	Acylcarnitines	0.1288	0.07	0.048	0.01	0.087	0.04
C18:2	Acylcarnitines	0.1236	0.06	0.0396	0.06	0.100	0.05
Trigonelline	Alkaloids	0.4678	2.02	0.1626	2.74	0.163	144.34
TMAO	Amine Oxides	0.0634	0.26	0.0826	5.15	0.190	451.07
Ala	Aminoacids	9	458.47	9	383.3	26.182	257.67
Arg	Aminoacids	2	120.94	2	54.34	3.526	22.22
Asn	Aminoacids	1.3012	18.57	1.516	44.91	43.000	55.67
Asp	Aminoacids	1.5	310.02	1.117	2.91	4.163	3.01
Cys	Aminoacids	0.0996	11.37	0.0872	72.88	0.315	186.99
Gln	Aminoacids	4.922	58.7	3.1068	561.56	23.640	370.85

Metabolite	Class	Stool LOD	Stool Mean	Plasma LOD	Plasma Mean	Urine LOD	Urine Mean
Glu	Aminoacids	2.4098	1139.23	2.2254	47.6	11.634	17.14
Gly	Aminoacids	6.452	161.43	3.1282	215.87	26.378	795.36
His	Aminoacids	1.1	17.79	1.1	75.76	4.977	390.25
Ile	Aminoacids	1.7596	103.43	1.0326	73.25	0.808	8.95
Leu	Aminoacids	1.6	204.9	1.4194	127.33	1.629	22.48
Lys	Aminoacids	1.4	258.06	1.4	175.77	8.270	91.18
Met	Aminoacids	1.9	77.44	1.1216	22.62	2.137	7.85
Phe	Aminoacids	1.355	84.36	1.4032	58.55	2.245	39.63
Pro	Aminoacids	3.542	104.45	4	211.05	6.092	13.16
Ser	Aminoacids	1.3656	98.45	1.6278	95.3	6.075	214.04
Thr	Aminoacids	1.6692	63.88	1.2414	111.3	5.281	81.13
Trp	Aminoacids	1.8	14.47	1.8	55.11	7.713	57.05
Tyr	Aminoacids	0.3	94.26	0.5552	67.59	5.928	72.52
Val	Aminoacids	4.4	150.05	3.926	204.78	13.857	38.55
1-Met-His	Aminoacids Related	0.0094	1.1	0.0074	5.04	0.089	145.18
3-Met-His	Aminoacids Related	0.0074	3.72	0.0122	5.72	0.527	127.8
5-AVA	Aminoacids Related	0.022	126.15	0.0206	0.04	0.023	1.21
AABA	Aminoacids Related	0.4646	15.84	0.4644	15.42	6.378	7.44
Ac-Orn	Aminoacids Related	0.08	1.76	0.08	0	0.717	0.52
ADMA	Aminoacids Related	0.0758	0.37	0.055	0.44	0.327	26.95
alpha-AAA	Aminoacids Related	0.2756	1.72	0.3	1.02	1.024	26.48
Anserine	Aminoacids Related	0.0076	1.36	0.0102	0.01	0.082	13.82
BABA	Aminoacids Related	0.0086	0.59	0.0318	0.05	0.032	5.65
Betaine	Aminoacids Related	3.346	0.95	1.082	33.52	0.755	131.25
c4-OH-Pro	Aminoacids Related	0.3604	0.28	0.2042	0.08	1.080	0.17
Carnosine	Aminoacids Related	0.3194	1.7	0.2056	0.04	0.445	20.27
Cit	Aminoacids Related	1.4338	90.41	0.8748	31.55	5.612	3.5
Creatinine	Aminoacids Related	3.332	20.06	2.8864	70.05		
Cystine	Aminoacids Related	0.0132	0.54	0.1082	71.89	0.246	155.04
DOPA	Aminoacids Related	0.1092	0.11	0.2032	0.01	0.722	0.35
HArg	Aminoacids Related	0.0114	0.08	0.016	2.15	0.016	1.44
HCys	Aminoacids Related	0.6884	4.2	0.6786	6.93	0.637	44.6
Kynurenine	Aminoacids Related	0.5206	0.13	0.6062	2.09	0.983	2.57
Met-SO	Aminoacids Related	0.4918	7.92	0.14	0.75	0.837	1.52
Nitro-Tyr	Aminoacids Related	0.3416	0.12	0.1822	0.02	1.078	0.08
Orn	Aminoacids Related	1.048	16.1	0.9004	102.3	6.253	11.02
PAG	Aminoacids Related	0.012	0.02	0.0068	0.01	0.053	1.75
PheAlaBetaine	Aminoacids Related	0.0024	0.06	0.0042	0.01	0.006	0.03
ProBetaine	Aminoacids Related	0.353	4.38	0.3746	7.54	0.443	189.24
Sarcosine	Aminoacids Related	0.3296	3.77	0.2354	2.56	1.025	1.78
SDMA	Aminoacids Related	0.0414	0.48	0.0178	0.54	1.036	33.46
t4-OH-Pro	Aminoacids Related	0.3914	3.61	0.3484	9.99	1.039	2.74
Taurine	Aminoacids Related	0.7	55.89	0.5868	43.79	3.241	232.36
TrpBetaine	Aminoacids Related	0.012	0.13	0.0176	0.46	0.035	0.9
CA	Bile Acids	0.0144	24.6	0.013	0.22	0.015	0.08
CDCA	Bile Acids	0.0426	19.34	0.0716	0.41	0.037	0.01
DCA	Bile Acids	0.0654	55.78	0.0866	0.33	0.017	0.42
GCA	Bile Acids	0.0042	0.62	0.0074	0.31	0.006	0.06
GCDCA	Bile Acids	0.0034	2.27	0.0032	1.2	0.009	0.01
GDCA	Bile Acids	0.0028	2.7	0.004	0.52	0.004	0.01
GLCA	Bile Acids	0.0044	0.1	0.0046	0.03	0.004	0

Metabolite	Class	Stool LOD	Mean	Plasma LOD	Mean	Urine LOD	Mean
GLCAS	Bile Acids	0.0024	0.49	0.002	0.28	0.002	0.68
GUDCA	Bile Acids	0.002	0.08	0.0034	0.11	0.004	0.02
TCA	Bile Acids	0.0104	0.78	0.0106	0.07	0.016	0.01
TCDCA	Bile Acids	0.0126	0.55	0.0112	0.15	0.016	0.01
TDCA	Bile Acids	0.0018	1.11	0.0008	0.07	0.002	0
TLCA	Bile Acids	0.0004	0.21	0.0008	0.01	0.001	0
TMCA	Bile Acids	0.0008	0.03	0.001	0.01	0.001	0.02
beta-Ala	Biogenic Amines	0.0878	16.53	0.0858	3.96	0.105	17.45
Dopamine	Biogenic Amines	0.2638	0.26	0.516	0.03	1.735	2.33
GABA	Biogenic Amines	0.0632	14.92	0.0686	0.16	0.055	1.45
Histamine	Biogenic Amines	0.7	5.22	0.439	0.12	1.111	0.28
PEA	Biogenic Amines	0.0034	0.5	0.001	0	0.112	0.05
Putrescine	Biogenic Amines	0.0458	11.35	0.0718	0.1	0.191	0.76
Serotonin	Biogenic Amines	0.0502	0.79	0.032	0.04	0.105	0.42
Spermidine	Biogenic Amines	0.18	14.54	0.226	0.18	0.283	0.22
Spermine	Biogenic Amines	0.3992	0.31	0.3722	0.14	0.336	1.09
AconAcid	Carboxylic Acids	0.1738	0.61	0.1934	7.79	0.465	447.25
DiCA(12:0)	Carboxylic Acids	0.4794	1.11	0.224	0.12	0.534	0.2
DiCA(14:0)	Carboxylic Acids	0.0572	0.06	0.0526	0.06	0.053	0.03
HipAcid	Carboxylic Acids	0.0186	4.36	0.018	9.76	0.460	1666.51
Lac	Carboxylic Acids	126.8	98.46	141	3868.24	142.283	234.41
OH-GlutAcid	Carboxylic Acids	1.728	2.98	1.7	0.79	2.188	16.75
Suc	Carboxylic Acids	2.852	223.55	3.676	3.55	6.657	17.68
Cer(d16:1/18:0)	Ceramides	0.0722	0.1	0.05	0.06	0.05	0
Cer(d16:1/20:0)	Ceramides	0.0232	0.03	0.01	0.05	0.01	0
Cer(d16:1/22:0)	Ceramides	0.01	0.31	0.01	0.24	0.01	0
Cer(d16:1/23:0)	Ceramides	0.0226	0.17	0.01	0.09	0.01	0
Cer(d16:1/24:0)	Ceramides	0.01	0.13	0.01	0.25	0.01	0
Cer(d18:1/14:0)	Ceramides	0.042	0.11	0.028	0.05	0.028	0
Cer(d18:1/16:0)	Ceramides	0.04	0.68	0.04	0.41	0.04	0.01
Cer(d18:1/18:0)	Ceramides	0.0226	0.66	0.01	0.18	0.01	0
Cer(d18:1/18:0(OH))	Ceramides	0.01	0.12	0.01	0	0.01	0
Cer(d18:1/18:1)	Ceramides	0.0348	0.26	0.0346	0.02	0.031	0.01
Cer(d18:1/20:0)	Ceramides	0.01	0.08	0.01	0.13	0.01	0
Cer(d18:1/20:0(OH))	Ceramides	0.21	1.67	0.21	0.76	0.21	0.15
Cer(d18:1/22:0)	Ceramides	0.0278	0.46	0.02	0.8	0.02	0
Cer(d18:1/23:0)	Ceramides	0.02	0.45	0.02	1.04	0.02	0
Cer(d18:1/24:0)	Ceramides	0.03	0.5	0.0498	2.53	0.03	0.01
Cer(d18:1/24:1)	Ceramides	0.01	0.7	0.01	1.25	0.01	0.01
Cer(d18:1/25:0)	Ceramides	0.04	0.35	0.04	0.37	0.04	0.01
Cer(d18:1/26:0)	Ceramides	0.01	0.09	0.01	0.05	0.01	0
Cer(d18:1/26:1)	Ceramides	0.01	0.02	0.01	0.01	0.01	0
Cer(d18:2/14:0)	Ceramides	0.0116	0	0.01	0	0.01	0
Cer(d18:2/16:0)	Ceramides	0.01	0.09	0.01	0.08	0.01	0
Cer(d18:2/18:0)	Ceramides	0.01	0.06	0.01	0.04	0.01	0
Cer(d18:2/18:1)	Ceramides	0.01	0.02	0.01	0	0.01	0
Cer(d18:2/20:0)	Ceramides	0.01	0.02	0.01	0.03	0.01	0
Cer(d18:2/22:0)	Ceramides	0.01	0.06	0.01	0.24	0.01	0
Cer(d18:2/23:0)	Ceramides	0.01	0.03	0.01	0.12	0.01	0
Cer(d18:2/24:0)	Ceramides	0.01	0.05	0.01	0.53	0.01	0
Cer(d18:2/24:1)	Ceramides	0.01	0.08	0.01	0.25	0.01	0

Metabolite	Class	Stool LOD	Mean	Plasma LOD	Mean	Urine LOD	Mean
CE(14:0)	Cholesterol Esters	1.2556	0.52	0.7932	31.93	0.678	0.2
CE(14:1)	Cholesterol Esters	0.2764	0.13	0.06	1.41	0.060	0.03
CE(15:0)	Cholesterol Esters	1.252	0.44	0.7714	10.86	0.823	0.17
CE(15:1)	Cholesterol Esters	1.1316	0.31	0.956	0.75	0.925	0.39
CE(16:0)	Cholesterol Esters	1.208	1.54	2.798	256.2	8.095	4.01
CE(16:1)	Cholesterol Esters	0.7826	0.26	2.631	93.52	0.761	0.12
CE(17:0)	Cholesterol Esters	1.1214	0.5	0.832	7.98	0.743	0.19
CE(17:1)	Cholesterol Esters	0.67	0.11	1.5414	8.06	0.350	0.05
CE(18:0)	Cholesterol Esters	0.6156	0.52	0.5258	20.6	4.484	2.3
CE(18:1)	Cholesterol Esters	1.83	3.01	17.284	508.39	3.926	0.35
CE(18:2)	Cholesterol Esters	2.94	3.11	69.738	1507.09	7.715	1.51
CE(18:3)	Cholesterol Esters	0.6782	0.28	1.5134	86.18	0.680	0.06
CE(20:0)	Cholesterol Esters	6.092	0.9	6.72	1.39	5.185	0.68
CE(20:1)	Cholesterol Esters	1.15	0.25	0.9	0.87	0.653	0.07
CE(20:3)	Cholesterol Esters	0.3904	0.2	1.3338	33.33	0.237	0.04
CE(20:4)	Cholesterol Esters	1.0312	0.47	9.902	291.26	1.627	0.27
CE(20:5)	Cholesterol Esters	0.924	0.31	3.0158	89.15	0.567	0.07
CE(22:0)	Cholesterol Esters	0.796	0.15	0.4512	0.29	0.390	0.11
CE(22:1)	Cholesterol Esters	0.6884	0.09	0.818	0.22	0.650	0.17
CE(22:2)	Cholesterol Esters	0.3392	0.07	0.1966	0.08	0.190	0.02
CE(22:5)	Cholesterol Esters	0.459	0.18	0.3366	3.08	0.361	0.07
CE(22:6)	Cholesterol Esters	0.4952	0.58	1.6254	43.47	0.275	0.05
p-Cresol-SO4	Cresols	0.01	0.7	0.0474	21.76	0.118	105.73
DG(14:0_14:0)	Diacylglycerols	0.1274	0.02	0.09	0.07	0.340	0.01
DG(14:0_18:1)	Diacylglycerols	1.0284	0.31	0.9116	0.55	1.020	0.06
DG(14:0_18:2)	Diacylglycerols	0.6428	0.32	0.58	0.29	0.532	0.06
DG(14:0_20:0)	Diacylglycerols	0.1818	0.1	0.5536	0.23	0.584	0.38
DG(14:1_18:1)	Diacylglycerols	0.347	0.19	0.13	0.1	0.130	0.02
DG(14:1_20:2)	Diacylglycerols	0.3278	0.1	0.288	0.01	0.253	0.01
DG(16:0_16:0)	Diacylglycerols	26.92	10.17	31.84	9.23	576.383	293.35
DG(16:0_16:1)	Diacylglycerols	1.203	0.77	0.4892	0.66	0.462	0.12
DG(16:0_18:1)	Diacylglycerols	1.0168	11.2	1.1128	5.14	0.807	0.46
DG(16:0_18:2)	Diacylglycerols	0.806	12.34	0.1782	2.03	0.218	0.05
DG(16:0_20:0)	Diacylglycerols	1.0846	0.54	0.728	0.6	8.675	4.08
DG(16:0_20:3)	Diacylglycerols	0.189	0.07	0.13	0.01	0.130	0
DG(16:0_20:4)	Diacylglycerols	0.3946	0.16	0.522	0.12	0.390	0.28
DG(16:1_18:0)	Diacylglycerols	0.4942	0.26	0.34	0.06	0.297	0.05
DG(16:1_18:1)	Diacylglycerols	1.5604	1.56	6.45	3.37	1.202	1.09
DG(16:1_18:2)	Diacylglycerols	0.3922	0.64	0.04	0.55	0.040	0.03
DG(16:1_20:0)	Diacylglycerols	0.6174	0.17	0.3154	0.05	0.290	0.03
DG(17:0_17:1)	Diacylglycerols	0.5348	0.13	0.2856	0.02	0.277	0.14
DG(17:0_18:1)	Diacylglycerols	0.9922	0.69	0.2504	0.61	0.255	0.12
DG(18:0_20:0)	Diacylglycerols	0.5184	0.22	1.026	0.34	4.202	2.26
DG(18:0_20:4)	Diacylglycerols	0.1564	0.14	0.1064	0.02	0.095	0.01
DG(18:1_18:1)	Diacylglycerols	0.759	27.22	0.3242	4.99	0.154	0.03
DG(18:1_18:2)	Diacylglycerols	1.404	52.08	0.39	7.72	0.390	0.04
DG(18:1_18:3)	Diacylglycerols	0.63	3.64	0.5286	0.73	0.441	0.05
DG(18:1_18:4)	Diacylglycerols	0.4854	0.1	0.5	0.01	0.500	0.01
DG(18:1_20:0)	Diacylglycerols	0.6286	0.77	0.2312	0.35	0.220	0.04
DG(18:1_20:1)	Diacylglycerols	0.1246	0.2	0.1032	0.12	0.110	0
DG(18:1_20:2)	Diacylglycerols	0.2862	0.05	0.28	0.03	0.280	0.01

Metabolite	Class	Stool LOD	Stool Mean	Plasma LOD	Plasma Mean	Urine LOD	Urine Mean
DG(18:1_20:3)	Diacylglycerols	0.3214	0.08	0.3024	0.07	0.290	0.01
DG(18:1_20:4)	Diacylglycerols	0.3816	0.6	0.29	0.25	0.186	0.09
DG(18:1_22:5)	Diacylglycerols	0.054	0.02	0.047	0.01	0.054	0
DG(18:1_22:6)	Diacylglycerols	0.7612	0.68	1.3988	0.65	0.254	0.19
DG(18:2_18:2)	Diacylglycerols	1.352	78.55	0.28	3.21	0.28	0.05
DG(18:2_18:3)	Diacylglycerols	0.608	2.97	0.11	0.23	0.11	0.03
DG(18:2_18:4)	Diacylglycerols	0.1632	0.05	0.13	0	0.13	0
DG(18:2_20:0)	Diacylglycerols	0.1798	0.22	0.01	0.03	0.01	0.01
DG(18:2_20:4)	Diacylglycerols	0.4624	0.26	0.18	0.1	0.18	0.02
DG(18:3_18:3)	Diacylglycerols	0.245	1.19	0.19	0.01	0.19	0.01
DG(18:3_20:2)	Diacylglycerols	0.2478	0.05	0.2	0.01	0.2	0.01
DG(21:0_22:6)	Diacylglycerols	0.4552	0.04	0.43	0.01	0.43	0.01
DG(22:1_22:2)	Diacylglycerols	0.2358	0.01	0.31	0	0.31	0
DG-O(14:0_18:2)	Diacylglycerols	0.935	0.19	0.7848	0.17	0.064	0.07
DG-O(16:0_18:1)	Diacylglycerols	0.4886	0.15	0.11	0.03	0.174	0.09
DG-O(16:0_20:4)	Diacylglycerols	0.1096	0.01	0.12	0	0.12	0
Cer(d18:0/18:0)	Dihydroceramides	0.0848	0.03	0.1374	0.01	0.174	0.08
Cer(d18:0/18:0(OH))	Dihydroceramides	1.064	2.49	1.502	0.23	0.844	0.34
Cer(d18:0/20:0)	Dihydroceramides	0.0932	0.09	0.0908	0.03	0.080	0.03
Cer(d18:0/22:0)	Dihydroceramides	0.1258	0.13	0.1484	0.1	0.098	0.01
Cer(d18:0/24:0)	Dihydroceramides	0.1	0.21	0.1	0.17	0.100	0
Cer(d18:0/24:1)	Dihydroceramides	0.1	0.18	0.1172	0.17	0.097	0.01
Cer(d18:0/26:1)	Dihydroceramides	0.07	0.02	0.07	0	0.080	0
Cer(d18:0/26:1(OH))	Dihydroceramides	1.25	0.18	0.9218	0.15	1.069	0.16
AA	Fatty Acids	0.0728	6.86	0.3484	3.05	0.189	0.05
DHA	Fatty Acids	0.0172	3.81	0.2178	4.33	0.027	0.05
EPA	Fatty Acids	0.0156	0.99	0.163	0.55	0.222	0.04
FA(12:0)	Fatty Acids	18.78	99.38	13.28	9.57	10.682	21.04
FA(14:0)	Fatty Acids	114.9	508.22	60.9	28.49	23.417	9
FA(16:0)	Fatty Acids	1333	1363.04	915.2	323.73	1620.000	435.24
FA(18:0)	Fatty Acids	1356.4	1223.9	847.4	247.69	1765.667	478.19
FA(18:1)	Fatty Acids	52.34	1869.99	24.02	157.02	76.783	12.63
FA(18:2)	Fatty Acids	77.1	673.75	13.736	106.86	48.017	7.33
FA(20:1)	Fatty Acids	2.868	81.03	0.9178	3.19	4.467	0.95
FA(20:2)	Fatty Acids	4.444	38.71	0.675	1.79	0.830	0.25
FA(20:3)	Fatty Acids	0.6944	13.08	0.2374	0.87	0.519	0.21
lysoPC a C14:0	Glycerophospholipids	11.7	3.19	11.02	4.91	14.733	5.01
lysoPC a C16:0	Glycerophospholipids	0.162	4.41	0.4664	91.91	0.164	0.26
lysoPC a C16:1	Glycerophospholipids	0.0724	0.15	0.0764	3	0.082	0.03
lysoPC a C17:0	Glycerophospholipids	0.0872	0.12	0.046	1.75	0.045	0.01
lysoPC a C18:0	Glycerophospholipids	0.268	1.31	0.183	26.73	0.241	0.15
lysoPC a C18:1	Glycerophospholipids	0.1662	1.75	0.2214	21.95	0.262	0.08
lysoPC a C18:2	Glycerophospholipids	0.1442	2.01	0.1554	30.68	0.126	0.08
lysoPC a C20:3	Glycerophospholipids	0.2018	0.06	0.1946	2.27	0.185	0.05
lysoPC a C20:4	Glycerophospholipids	0.07	0.03	0.0888	6.46	0.051	0.02
lysoPC a C24:0	Glycerophospholipids	0.2246	0.04	0.1444	0.18	0.297	0.04
lysoPC a C26:0	Glycerophospholipids	0.1436	0.03	0.242	0.33	0.171	0.01
lysoPC a C26:1	Glycerophospholipids	0.056	0.01	0.0618	0.16	0.056	0
lysoPC a C28:0	Glycerophospholipids	0.2704	0.07	0.3664	0.34	0.923	0.26
lysoPC a C28:1	Glycerophospholipids	0.209	0.03	0.289	0.41	0.289	0.01
PC aa C24:0	Glycerophospholipids	0.0656	0.02	0.101	0.13	0.078	0.03

Metabolite	Class	Stool LOD	Mean	Plasma LOD	Mean	Urine LOD	Mean
PC aa C26:0	Glycerophospholipids	1.0432	0.39	1.132	0.82	1.082	0.35
PC aa C28:1	Glycerophospholipids	0.351	0.04	0.3544	3.91	0.229	0.03
PC aa C30:0	Glycerophospholipids	0.142	0.37	0.144	4.75	0.127	0.05
PC aa C30:2	Glycerophospholipids	0.0118	0	0.013	0.04	0.013	0
PC aa C32:0	Glycerophospholipids	0.0782	0.54	0.1198	16.63	0.060	0.04
PC aa C32:1	Glycerophospholipids	0.0454	0.16	0.0602	21.75	0.065	0.02
PC aa C32:2	Glycerophospholipids	0.011	0.12	0.0132	5.24	0.011	0
PC aa C32:3	Glycerophospholipids	0.011	0.02	0.011	0.63	0.011	0
PC aa C34:1	Glycerophospholipids	0.0908	1.63	0.6848	251.88	0.072	0.29
PC aa C34:2	Glycerophospholipids	0.0884	1.79	0.9818	405.41	0.090	0.47
PC aa C34:3	Glycerophospholipids	0.0412	0.21	0.0566	19.07	0.041	0.01
PC aa C34:4	Glycerophospholipids	0.006	0.02	0.0126	2.25	0.035	0.01
PC aa C36:0	Glycerophospholipids	0.2728	0.12	0.2888	1.56	0.251	0.09
PC aa C36:1	Glycerophospholipids	0.1006	0.38	0.4952	56.63	0.041	0.1
PC aa C36:2	Glycerophospholipids	0.0864	1.09	0.587	236.65	0.090	0.3
PC aa C36:3	Glycerophospholipids	0.0328	1.26	0.2818	139.8	0.036	0.14
PC aa C36:4	Glycerophospholipids	0.0522	1.7	0.4368	204.98	0.038	0.19
PC aa C36:5	Glycerophospholipids	0.0334	0.28	0.0982	31.3	0.786	0.22
PC aa C36:6	Glycerophospholipids	0.0182	0.05	0.029	1.09	0.017	0
PC aa C38:0	Glycerophospholipids	0.0792	0.05	0.1114	2.5	0.067	0.01
PC aa C38:1	Glycerophospholipids	0.027	0.02	0.0396	0.87	0.027	0.01
PC aa C38:3	Glycerophospholipids	0.0488	0.06	0.1512	51.48	0.050	0.06
PC aa C38:4	Glycerophospholipids	0.0452	0.07	0.1974	110.45	0.044	0.12
PC aa C38:5	Glycerophospholipids	0.0502	0.04	0.1342	56.99	0.048	0.05
PC aa C38:6	Glycerophospholipids	0.0278	0.06	0.1108	79.58	0.028	0.06
PC aa C40:1	Glycerophospholipids	0.4536	0.14	0.4546	0.33	0.414	0.14
PC aa C40:2	Glycerophospholipids	0.027	0.01	0.0342	0.26	0.025	0
PC aa C40:3	Glycerophospholipids	0.01	0.01	0.0158	0.47	0.012	0
PC aa C40:4	Glycerophospholipids	0.015	0.01	0.0252	3.05	0.016	0.01
PC aa C40:5	Glycerophospholipids	0.067	0.01	0.023	8.53	0.067	0.01
PC aa C40:6	Glycerophospholipids	0.2886	0.12	0.3402	23.71	0.277	0.11
PC aa C42:0	Glycerophospholipids	0.0622	0.02	0.0762	0.47	0.066	0.02
PC aa C42:1	Glycerophospholipids	0.0124	0.01	0.0256	0.24	0.012	0.01
PC aa C42:2	Glycerophospholipids	0.0722	0.03	0.0956	0.19	0.144	0.03
PC aa C42:4	Glycerophospholipids	0.0186	0	0.019	0.16	0.019	0
PC aa C42:5	Glycerophospholipids	0.004	0.01	0.019	0.3	0.009	0
PC aa C42:6	Glycerophospholipids	0.1452	0.05	0.133	0.41	0.119	0.04
PC ae C30:0	Glycerophospholipids	0.1768	0.1	0.14	0.4	0.110	0.04
PC ae C30:1	Glycerophospholipids	0.028	0.02	0.0182	0.16	0.028	0
PC ae C30:2	Glycerophospholipids	0.0146	0.01	0.0194	0.11	0.011	0
PC ae C32:1	Glycerophospholipids	0.01	0.07	0.029	3.2	0.009	0.01
PC ae C32:2	Glycerophospholipids	0.077	0.03	0.0612	0.85	0.077	0
PC ae C34:0	Glycerophospholipids	0.036	0.18	0.0388	1.72	0.027	0.01
PC ae C34:1	Glycerophospholipids	0.028	0.26	0.0586	12.49	0.030	0.03
PC ae C34:2	Glycerophospholipids	0.02	0.21	0.0392	13.67	0.020	0.02
PC ae C34:3	Glycerophospholipids	0.0374	0.08	0.052	8.65	0.039	0.01
PC ae C36:0	Glycerophospholipids	0.1882	0.09	0.211	0.77	0.161	0.06
PC ae C36:1	Glycerophospholipids	0.0648	0.11	0.1442	9.37	0.046	0.03
PC ae C36:2	Glycerophospholipids	0.045	0.12	0.0956	15.26	0.042	0.04
PC ae C36:3	Glycerophospholipids	0.0182	0.08	0.0292	8.26	0.014	0.01
PC ae C36:4	Glycerophospholipids	0.0448	0.07	0.0996	18.7	0.051	0.03

Metabolite	Class	Stool LOD	Mean	Plasma LOD	Mean	Urine LOD	Mean
PC ae C36:5	Glycerophospholipids	0.0266	0.05	0.0484	12.27	0.024	0.02
PC ae C38:0	Glycerophospholipids	0.1642	0.07	0.1928	2.12	0.155	0.06
PC ae C38:1	Glycerophospholipids	0.0172	0.02	0.0602	0.21	0.018	0.01
PC ae C38:2	Glycerophospholipids	0.025	0.03	0.0544	1.82	0.026	0.01
PC ae C38:3	Glycerophospholipids	0.0178	0.04	0.0294	4.26	0.023	0.02
PC ae C38:4	Glycerophospholipids	0.0678	0.06	0.1094	13.69	0.094	0.04
PC ae C38:5	Glycerophospholipids	0.0434	0.06	0.0864	18.02	0.587	0.09
PC ae C38:6	Glycerophospholipids	0.0178	0.03	0.0342	7.23	0.018	0.06
PC ae C40:1	Glycerophospholipids	0.037	0.02	0.039	1.17	0.054	0.01
PC ae C40:2	Glycerophospholipids	0.0206	0.02	0.0358	1.89	0.025	0.01
PC ae C40:3	Glycerophospholipids	0.0142	0.01	0.0236	1.04	0.017	0.01
PC ae C40:4	Glycerophospholipids	0.0874	0.04	0.1086	2.15	0.088	0.03
PC ae C40:5	Glycerophospholipids	0.017	0.01	0.0298	3.16	0.017	0.01
PC ae C40:6	Glycerophospholipids	0.0252	0.02	0.0364	4.12	0.022	0.01
PC ae C42:0	Glycerophospholipids	1.012	0.36	0.9662	0.62	0.895	0.31
PC ae C42:1	Glycerophospholipids	0.0854	0.04	0.1068	0.33	0.079	0.02
PC ae C42:2	Glycerophospholipids	0.018	0.01	0.0252	0.51	0.018	0
PC ae C42:3	Glycerophospholipids	0.035	0.01	0.035	0.71	0.031	0.01
PC ae C42:4	Glycerophospholipids	0.167	0	0.167	0.74	0.167	0
PC ae C42:5	Glycerophospholipids	0.71	0.24	0.7152	1.74	0.684	0.24
PC ae C44:3	Glycerophospholipids	0.0492	0.02	0.0508	0.14	0.045	0.02
PC ae C44:4	Glycerophospholipids	0.1046	0.04	0.1226	0.34	0.103	0.04
PC ae C44:5	Glycerophospholipids	0.0692	0.02	0.0604	1.38	0.075	0.03
PC ae C44:6	Glycerophospholipids	0.0554	0.03	0.074	0.92	0.062	0.02
Hex2Cer(d18:1/14:0)	Glycosylceramides	0.05	0.04	0.05	0.18	0.05	0
Hex2Cer(d18:1/16:0)	Glycosylceramides	0.05	0.59	0.05	2.42	0.05	0.05
Hex2Cer(d18:1/18:0)	Glycosylceramides	0.0172	0.36	0.01	0.17	0.01	0
Hex2Cer(d18:1/20:0)	Glycosylceramides	0.023	0.06	0.01	0.06	0.01	0
Hex2Cer(d18:1/22:0)	Glycosylceramides	0.01	0.31	0.01	0.16	0.01	0
Hex2Cer(d18:1/24:0)	Glycosylceramides	0.01	0.27	0.01	0.16	0.01	0.01
Hex2Cer(d18:1/24:1)	Glycosylceramides	0.01	0.22	0.0234	0.38	0.01	0.02
Hex2Cer(d18:1/26:0)	Glycosylceramides	0.01	0.02	0.01	0	0.01	0
Hex2Cer(d18:1/26:1)	Glycosylceramides	0.01	0.01	0.01	0	0.01	0
Hex3Cer(d18:1/16:0)	Glycosylceramides	0.01	0.07	0.01	1.03	0.01	0.01
Hex3Cer(d18:1/18:0)	Glycosylceramides	0.02	0.04	0.02	0.1	0.02	0
Hex3Cer(d18:1_20:0)	Glycosylceramides	0.04	0.03	0.04	0.04	0.04	0
Hex3Cer(d18:1_22:0)	Glycosylceramides	0.02	0.03	0.02	0.22	0.02	0
Hex3Cer(d18:1/24:1)	Glycosylceramides	0.01	0.04	0.01	0.38	0.01	0
Hex3Cer(d18:1/26:1)	Glycosylceramides	0.02	0.03	0.02	0.03	0.02	0
HexCer(d16:1/22:0)	Glycosylceramides	0.0254	0.04	0.01	0.16	0.01	0
HexCer(d16:1/24:0)	Glycosylceramides	0.02	0.03	0.02	0.07	0.02	0
HexCer(d18:1/14:0)	Glycosylceramides	0.02	0.08	0.02	0.01	0.02	0
HexCer(d18:1/16:0)	Glycosylceramides	0.03	0.74	0.03	1.03	0.03	0.01
HexCer(d18:1/18:0)	Glycosylceramides	0.01	0.21	0.01	0.14	0.01	0
HexCer(d18:1/18:1)	Glycosylceramides	0.02	0.07	0.0624	0.04	0.02	0.01
HexCer(d18:1/20:0)	Glycosylceramides	0.03	0.13	0.03	0.26	0.03	0.02
HexCer(d18:1/22:0)	Glycosylceramides	0.06	0.88	0.06	2.91	0.06	0.02
HexCer(d18:1/23:0)	Glycosylceramides	0.09	0.63	0.09	1.63	0.09	0.01
HexCer(d18:1/24:0)	Glycosylceramides	0.03	0.33	0.03	1.66	0.03	0.01
HexCer(d18:1/24:1)	Glycosylceramides	0.18	1.94	0.18	3.35	0.177	0.04
HexCer(d18:1/26:0)	Glycosylceramides	0.06	0.12	0.06	0.06	0.06	0.01

Metabolite	Class	Stool LOD	Mean	Plasma LOD	Mean	Urine LOD	Mean
HexCer(d18:1/26:1)	Glycosylceramides	0.1366	0.12	0.07	0.03	0.07	0.01
HexCer(d18:2/16:0)	Glycosylceramides	0.01	0.11	0.01	0.02	0.01	0
HexCer(d18:2/18:0)	Glycosylceramides	0.0412	0.02	0.01	0.02	0.02	0.01
HexCer(d18:2/20:0)	Glycosylceramides	0.02	0.01	0.02	0.02	0.02	0
HexCer(d18:2/22:0)	Glycosylceramides	0.05	0.05	0.05	0.48	0.05	0
HexCer(d18:2/23:0)	Glycosylceramides	0.07	0.04	0.07	0.23	0.07	0
HexCer(d18:2/24:0)	Glycosylceramides	0.08	0.07	0.08	0.79	0.08	0
AbsAcid	Hormones	0.0418	0.12	0.2988	0.1	0.038	2.79
Cortisol	Hormones	0.0416	0.03	0.049	0.26	0.041	0.11
Cortisone	Hormones	0.0696	0.03	0.074	0.07	0.073	0.23
DHEAS	Hormones	0.0406	0.76	0.0396	2.91	0.698	0.52
3-IAA	Indoles Derivatives	0.0886	7.01	0.0768	2.34	0.131	16.67
3-IPA	Indoles Derivatives	0.021	1.56	0.0188	1	0.092	0.03
Indole	Indoles Derivatives	0.0312	0.29	0.0378	4.8	0.062	112.84
Ind-SO4	Indoles Derivatives	34.52	55.46	40.9	57.98	123.333	138.32
Hypoxanthine	Nucleobases Related	0.7292	59.89	0.5934	4.74	1.215	345.4
Xanthine	Nucleobases Related	0.2474	77	0.2638	0.75	0.701	395.01
SM (OH) C14:1	Sphingolipids	0.0576	0.05	0.054	7.19	0.066	0.02
SM (OH) C16:1	Sphingolipids	0.0072	0.05	0.0202	3.61	0.004	0.01
SM (OH) C22:1	Sphingolipids	0.1146	0.06	0.5384	10.75	0.025	0.04
SM (OH) C22:2	Sphingolipids	0.0132	0.02	0.0746	9.48	0.009	0.02
SM (OH) C24:1	Sphingolipids	0.0126	0.02	0.0928	1	0.015	0.01
SM C16:0	Sphingolipids	0.1976	1.09	0.4284	108.23	0.104	0.36
SM C16:1	Sphingolipids	0.033	0.04	0.0682	16.01	0.030	0.04
SM C18:0	Sphingolipids	0.0908	0.26	0.1962	22.02	0.055	0.06
SM C18:1	Sphingolipids	0.013	0.02	0.0348	10.3	0.008	0.02
SM C20:2	Sphingolipids	0.008	0.01	0.008	0.32	0.020	0
SM C22:3	Sphingolipids	0.0054	0.01	0.004	0.14	0.004	0
SM C24:0	Sphingolipids	0.1956	0.13	1.1296	15.08	0.075	0.1
SM C24:1	Sphingolipids	0.0894	0.12	0.373	37.93	0.040	0.12
SM C26:0	Sphingolipids	0.0094	0.01	0.036	0.13	0.007	0.01
SM C26:1	Sphingolipids	0.0102	0.01	0.01	0.34	0.012	0
H1	Sugars	522.4	407.71	316.4	4444.09	498.500	3007.29
TG(14:0_32:2)	Triacylglycerols	0.811	0.09	0.6792	3.85	0.900	0.02
TG(14:0_34:0)	Triacylglycerols	0.3404	0.16	0.4828	7.29	0.788	0.29
TG(14:0_34:1)	Triacylglycerols	0.1956	0.5	1.224	44.06	0.232	0.09
TG(14:0_34:2)	Triacylglycerols	0.2372	0.26	0.2774	22.27	0.185	0.03
TG(14:0_34:3)	Triacylglycerols	0.231	0.03	0.288	4.69	0.231	0
TG(14:0_35:1)	Triacylglycerols	0.1896	0.04	0.2042	1.18	0.219	0.05
TG(14:0_35:2)	Triacylglycerols	0.2174	0.02	0.221	1.21	0.191	0.01
TG(14:0_36:1)	Triacylglycerols	0.2228	0.13	0.2316	9.21	0.187	0.02
TG(14:0_36:2)	Triacylglycerols	0.2006	0.46	1.112	37.67	0.17	0.02
TG(14:0_36:3)	Triacylglycerols	0.208	0.4	0.6688	23.94	0.208	0.01
TG(14:0_36:4)	Triacylglycerols	0.135	0.36	0.2024	7.05	0.135	0
TG(14:0_38:4)	Triacylglycerols	0.1	0.01	0.1	0.81	0.1	0
TG(14:0_38:5)	Triacylglycerols	0.1	0	0.1	0.7	0.1	0.01
TG(14:0_39:3)	Triacylglycerols	0.0926	0	0.0978	0.01	0.1	0
TG(16:0_28:1)	Triacylglycerols	0.3306	0.29	0.3392	6.24	0.323	0.03
TG(16:0_28:2)	Triacylglycerols	0.2744	0.1	0.1	1.61	0.100	0.11
TG(16:0_30:2)	Triacylglycerols	0.239	0.16	0.2806	4.86	0.185	0.02
TG(16:0_32:0)	Triacylglycerols	5.424	4.01	4.134	53.56	9.502	4.85

Metabolite	Class	Stool LOD	Mean	Plasma LOD	Mean	Urine LOD	Mean
TG(16:0_32:1)	Triacylglycerols	0.6672	0.66	1.4866	77.82	0.489	0.23
TG(16:0_32:2)	Triacylglycerols	0.3286	0.36	0.8978	30.28	0.303	0.05
TG(16:0_32:3)	Triacylglycerols	0.117	0.04	0.2068	4.93	0.117	0
TG(16:0_33:1)	Triacylglycerols	0.3694	0.18	0.5186	12.08	0.229	0.14
TG(16:0_33:2)	Triacylglycerols	0.203	0.19	0.2634	4.18	0.203	0.01
TG(16:0_34:0)	Triacylglycerols	4.028	1.72	2.34	36.4	11.395	5.68
TG(16:0_34:1)	Triacylglycerols	1.0876	9.2	6.154	235.59	1.289	0.66
TG(16:0_34:2)	Triacylglycerols	0.8174	10.72	4.644	160.87	0.456	0.27
TG(16:0_34:3)	Triacylglycerols	0.341	1.23	0.9268	38.57	0.341	0.03
TG(16:0_34:4)	Triacylglycerols	0.1	0.07	0.1	4.4	0.100	0
TG(16:0_35:1)	Triacylglycerols	0.2458	0.25	0.4192	7.8	0.354	0.09
TG(16:0_35:2)	Triacylglycerols	0.219	0.23	0.345	9.1	0.219	0.01
TG(16:0_35:3)	Triacylglycerols	0.1	0.1	0.1458	3.04	0.100	0
TG(16:0_36:2)	Triacylglycerols	1.3268	30.89	9.382	360.63	0.490	0.32
TG(16:0_36:3)	Triacylglycerols	1.0162	26.86	6.1188	243.51	0.404	0.26
TG(16:0_36:4)	Triacylglycerols	0.6194	23.8	1.6884	70.83	0.425	0.08
TG(16:0_36:5)	Triacylglycerols	0.149	3.33	0.3432	10.57	0.149	0.02
TG(16:0_36:6)	Triacylglycerols	0.179	3.08	0.1456	1.4	0.229	0.06
TG(16:0_37:3)	Triacylglycerols	0.1	0.4	0.1	0.92	0.100	0
TG(16:0_38:1)	Triacylglycerols	0.1	0.16	0.1358	2.07	0.100	0.01
TG(16:0_38:2)	Triacylglycerols	0.1136	0.36	0.1312	4.57	0.100	0
TG(16:0_38:3)	Triacylglycerols	0.1064	0.22	0.2358	5.51	0.100	0
TG(16:0_38:4)	Triacylglycerols	0.1	0.05	0.1	7.37	0.131	0.02
TG(16:0_38:5)	Triacylglycerols	0.1	0.03	0.26	7.72	0.462	0.17
TG(16:0_38:6)	Triacylglycerols	0.1	0.01	0.1	4.53	0.181	0.06
TG(16:0_38:7)	Triacylglycerols	0.1	0.03	0.1	1.14	0.225	0.07
TG(16:0_40:6)	Triacylglycerols	0.1	0.01	0.1	4.74	0.181	0.06
TG(16:0_40:7)	Triacylglycerols	0.66	0.01	0.66	3.98	0.660	0.01
TG(16:0_40:8)	Triacylglycerols	0.1466	0.01	0.1136	1.39	0.100	0.01
TG(16:1_28:0)	Triacylglycerols	0.1552	0.07	0.2522	2.15	0.148	0.07
TG(16:1_30:1)	Triacylglycerols	0.3886	0.13	0.582	3.48	0.333	0.11
TG(16:1_32:0)	Triacylglycerols	0.5224	0.18	1.0404	14.37	0.337	0.13
TG(16:1_32:1)	Triacylglycerols	0.5464	0.25	1.241	19.94	0.442	0.19
TG(16:1_32:2)	Triacylglycerols	0.2526	0.26	0.45	5.08	0.236	0.05
TG(16:1_33:1)	Triacylglycerols	0.3618	0.11	0.5098	2.63	0.254	0.06
TG(16:1_34:0)	Triacylglycerols	0.2426	0.12	0.3578	10.54	0.241	0.02
TG(16:1_34:1)	Triacylglycerols	0.5744	0.73	2.3676	75.76	0.454	0.11
TG(16:1_34:2)	Triacylglycerols	0.3634	0.79	1.1828	37.63	0.350	0.06
TG(16:1_34:3)	Triacylglycerols	0.1	0.09	0.1566	6.6	0.100	0.01
TG(16:1_36:1)	Triacylglycerols	0.1578	0.33	0.3818	9.58	0.150	0.01
TG(16:1_36:2)	Triacylglycerols	0.2626	1.67	1.3716	43.82	0.235	0.02
TG(16:1_36:3)	Triacylglycerols	0.1558	1.08	0.652	27.11	0.110	0.02
TG(16:1_36:4)	Triacylglycerols	0.1	0.7	0.2726	8.26	0.100	0
TG(16:1_36:5)	Triacylglycerols	0.1124	0.08	0.1	1.51	0.100	0
TG(16:1_38:3)	Triacylglycerols	0.1	0.09	0.1	0.78	0.100	0
TG(16:1_38:4)	Triacylglycerols	0.1	0.13	0.1	1.31	0.100	0
TG(16:1_38:5)	Triacylglycerols	0.1	0.1	0.1	1.29	0.100	0.01
TG(17:0_32:1)	Triacylglycerols	0.2072	0.06	0.2434	2.03	0.161	0.07
TG(17:0_34:1)	Triacylglycerols	0.1776	0.13	0.2506	6.12	0.184	0.02
TG(17:0_34:2)	Triacylglycerols	0.171	0.13	0.13	3.62	0.130	0.01
TG(17:0_34:3)	Triacylglycerols	0.1278	0.02	0.103	0.75	0.103	0

Metabolite	Class	Stool LOD	Stool Mean	Plasma LOD	Plasma Mean	Urine LOD	Urine Mean
TG(17:0_36:3)	Triacylglycerols	0.1	0.26	0.1	4.52	0.100	0
TG(17:0_36:4)	Triacylglycerols	0.1	0.23	0.114	1.31	0.100	0
TG(17:1_32:1)	Triacylglycerols	0.1824	0.03	0.167	1.97	0.231	0.27
TG(17:1_34:1)	Triacylglycerols	0.167	0.12	0.3678	7.24	0.161	0.01
TG(17:1_34:2)	Triacylglycerols	0.118	0.07	0.2322	3.43	0.118	0.01
TG(17:1_34:3)	Triacylglycerols	0.1	0.02	0.1	0.64	0.1	0
TG(17:1_36:3)	Triacylglycerols	0.1	0.19	0.1	2.22	0.1	0
TG(17:1_36:4)	Triacylglycerols	0.1	0.15	0.1	0.75	0.1	0
TG(17:1_36:5)	Triacylglycerols	0.1	0.03	0.1	0.12	0.1	0.01
TG(17:1_38:5)	Triacylglycerols	0.1	0.01	0.1	0.12	0.1	0
TG(17:1_38:6)	Triacylglycerols	0.1	0.01	0.1	0.07	0.1	0.01
TG(17:1_38:7)	Triacylglycerols	0.1	0	0.1	0	0.1	0
TG(17:2_34:2)	Triacylglycerols	0.104	0.03	0.104	0.24	0.104	0
TG(17:2_34:3)	Triacylglycerols	0.1	0.04	0.1	0.19	0.1	0
TG(17:2_36:2)	Triacylglycerols	0.1	0.11	0.1	0.37	0.1	0
TG(17:2_36:3)	Triacylglycerols	0.1096	0.11	0.1	0.24	0.1	0
TG(17:2_36:4)	Triacylglycerols	0.1	0.21	0.1	0.44	0.1	0.02
TG(17:2_38:5)	Triacylglycerols	0.1	0.03	0.1	0.12	0.1	0
TG(17:2_38:6)	Triacylglycerols	0.1	0.01	0.1	0.09	0.1	0
TG(17:2_38:7)	Triacylglycerols	0.1	0.01	0.1	0.03	0.168	0.01
TG(18:0_30:0)	Triacylglycerols	0.4896	0.26	0.4576	4.88	0.743	0.29
TG(18:0_30:1)	Triacylglycerols	0.2022	0.14	0.153	3.15	0.145	0.01
TG(18:0_32:0)	Triacylglycerols	1.806	0.63	1.1086	9.6	5.749	2.9
TG(18:0_32:1)	Triacylglycerols	0.187	0.32	0.4988	10.95	0.191	0.06
TG(18:0_32:2)	Triacylglycerols	0.1	0.19	0.1354	3.15	0.100	0.01
TG(18:0_34:2)	Triacylglycerols	0.2756	2.66	0.7188	17.94	0.252	0.02
TG(18:0_34:3)	Triacylglycerols	0.1154	0.33	0.1602	3.81	0.100	0
TG(18:0_36:1)	Triacylglycerols	0.4062	1.76	0.4686	7.77	0.486	0.14
TG(18:0_36:2)	Triacylglycerols	0.6656	10.27	1.0036	27.6	0.347	0.03
TG(18:0_36:3)	Triacylglycerols	0.3942	7.54	0.8134	23.09	0.271	0.02
TG(18:0_36:4)	Triacylglycerols	0.1984	6.55	0.274	7.9	0.100	0.01
TG(18:0_36:5)	Triacylglycerols	0.1	0.94	0.1	1.38	0.100	0.03
TG(18:0_38:6)	Triacylglycerols	0.1452	0.01	0.1	1.36	0.146	0.13
TG(18:0_38:7)	Triacylglycerols	0.1	0.02	0.1	0.28	0.160	0.03
TG(18:1_26:0)	Triacylglycerols	0.222	2.19	0.264	6.23	0.222	0.02
TG(18:1_28:1)	Triacylglycerols	0.298	0.37	0.3838	6.59	0.271	0.01
TG(18:1_30:0)	Triacylglycerols	0.441	0.58	1.3878	56.53	0.573	0.19
TG(18:1_30:1)	Triacylglycerols	0.3156	0.59	0.7602	32.66	0.317	0.05
TG(18:1_30:2)	Triacylglycerols	0.208	0.17	0.2178	5.76	0.165	0.01
TG(18:1_31:0)	Triacylglycerols	1.3036	0.79	1.5708	7.47	0.921	0.6
TG(18:1_32:0)	Triacylglycerols	0.9922	3.61	4.364	146.45	10.395	3.69
TG(18:1_32:1)	Triacylglycerols	0.5494	1.36	4.39	158.91	0.361	0.12
TG(18:1_32:2)	Triacylglycerols	0.295	0.65	1.0416	36.32	0.274	0.02
TG(18:1_32:3)	Triacylglycerols	0.127	0.1	0.1734	4.55	0.127	0
TG(18:1_33:0)	Triacylglycerols	0.187	0.18	0.3234	8.21	0.265	0.05
TG(18:1_33:1)	Triacylglycerols	0.2144	0.31	0.5764	20	0.186	0.03
TG(18:1_33:2)	Triacylglycerols	0.134	0.27	0.1656	6.38	0.133	0.01
TG(18:1_33:3)	Triacylglycerols	0.1498	0.08	0.101	0.85	0.101	0
TG(18:1_34:1)	Triacylglycerols	2.1024	49	16.854	604.84	0.725	0.51
TG(18:1_34:2)	Triacylglycerols	1.1928	26.12	6.6706	285.9	0.288	0.27
TG(18:1_34:3)	Triacylglycerols	0.2946	3	1.148	45.84	0.291	0.03

Metabolite	Class	Stool LOD	Mean	Plasma LOD	Mean	Urine LOD	Mean
TG(18:1_34:4)	Triacylglycerols	0.1	0.21	0.1522	4.79	0.100	0
TG(18:1_35:2)	Triacylglycerols	0.115	0.89	0.3512	9.43	0.124	0.01
TG(18:1_35:3)	Triacylglycerols	0.1272	0.23	0.16	2.51	0.100	0
TG(18:1_36:0)	Triacylglycerols	0.5542	2.33	0.5716	8.53	3.730	1.38
TG(18:1_36:1)	Triacylglycerols	1.5556	35.23	1.1782	66.16	0.368	0.09
TG(18:1_36:2)	Triacylglycerols	5.934	173.42	4.868	209.3	0.748	0.2
TG(18:1_36:3)	Triacylglycerols	2.6	85.44	3.2462	129.23	0.349	0.14
TG(18:1_36:4)	Triacylglycerols	1.2162	48.59	0.6438	43.76	0.322	0.07
TG(18:1_36:5)	Triacylglycerols	0.1806	5.32	0.1476	8.66	0.100	0.11
TG(18:1_36:6)	Triacylglycerols	0.1	3.03	0.1	1.28	0.189	0.29
TG(18:1_38:5)	Triacylglycerols	0.1	0.02	0.1734	8.8	0.108	0
TG(18:1_38:6)	Triacylglycerols	0.1	0.02	0.1	4.64	0.1	0
TG(18:1_38:7)	Triacylglycerols	0.1	0.11	0.1	0.95	0.1	0
TG(18:2_28:0)	Triacylglycerols	0.113	0.18	0.127	5.8	0.1	0
TG(18:2_30:0)	Triacylglycerols	0.2676	0.25	0.5886	18.71	0.265	0.02
TG(18:2_30:1)	Triacylglycerols	0.2206	0.16	0.3682	10	0.211	0.01
TG(18:2_31:0)	Triacylglycerols	2.012	0.35	2.588	2.71	2.643	0.59
TG(18:2_32:0)	Triacylglycerols	0.4746	4.01	1.4372	50.39	1.245	0.41
TG(18:2_32:1)	Triacylglycerols	0.3988	0.69	1.2636	51.31	0.416	0.05
TG(18:2_32:2)	Triacylglycerols	0.2242	0.67	0.3386	11.71	0.212	0.01
TG(18:2_33:0)	Triacylglycerols	0.1	0.1	0.1	3.08	0.100	0
TG(18:2_33:1)	Triacylglycerols	0.138	0.17	0.2248	6.88	0.138	0.01
TG(18:2_33:2)	Triacylglycerols	0.1	0.29	0.1	2.15	0.100	0
TG(18:2_34:0)	Triacylglycerols	0.4626	3.77	0.9182	31.82	1.763	0.58
TG(18:2_34:1)	Triacylglycerols	0.9844	23.78	5.0596	228.49	0.955	0.23
TG(18:2_34:2)	Triacylglycerols	0.8368	35.76	2.391	101.09	0.158	0.13
TG(18:2_34:3)	Triacylglycerols	0.2168	3.36	0.2118	15.14	0.207	0.01
TG(18:2_34:4)	Triacylglycerols	0.1	0.34	0.1	1.62	0.100	0
TG(18:2_35:1)	Triacylglycerols	0.131	0.7	0.1784	5.06	0.135	0
TG(18:2_35:2)	Triacylglycerols	0.1	0.62	0.1	3.19	0.100	0
TG(18:2_35:3)	Triacylglycerols	0.1476	0.28	0.1	0.91	0.100	0
TG(18:2_36:0)	Triacylglycerols	0.1878	1.16	0.1294	3.37	0.488	0.15
TG(18:2_36:1)	Triacylglycerols	0.5002	10.8	0.8668	26.65	0.323	0.03
TG(18:2_36:2)	Triacylglycerols	1.3998	54.2	2.0208	68.94	0.444	0.09
TG(18:2_36:3)	Triacylglycerols	1.7528	81.86	0.636	42.01	0.428	0.07
TG(18:2_36:4)	Triacylglycerols	1.1404	75.46	0.7992	15.98	0.629	0.03
TG(18:2_36:5)	Triacylglycerols	0.1	9.18	0.1666	3.28	0.1	0
TG(18:2_38:4)	Triacylglycerols	0.1	0.14	0.1	2.66	0.1	0
TG(18:2_38:5)	Triacylglycerols	0.1	0.02	0.1	3.43	0.1	0
TG(18:2_38:6)	Triacylglycerols	0.1	0.01	0.1	1.88	0.1	0
TG(18:3_30:0)	Triacylglycerols	0.123	0.01	0.123	2.86	0.123	0
TG(18:3_32:0)	Triacylglycerols	0.1354	0.32	0.2718	6.58	0.13	0.02
TG(18:3_32:1)	Triacylglycerols	0.1	0.06	0.1	7.25	0.1	0.02
TG(18:3_33:2)	Triacylglycerols	0.1	0.04	0.1	0.24	0.1	0
TG(18:3_34:0)	Triacylglycerols	0.2006	0.35	0.5436	3.93	0.108	0.06
TG(18:3_34:1)	Triacylglycerols	0.223	2.75	0.3754	29.43	0.203	0.03
TG(18:3_34:2)	Triacylglycerols	0.1752	3.34	0.3552	13.88	0.118	0.01
TG(18:3_34:3)	Triacylglycerols	0.1784	4.68	0.1	2.28	0.1	0
TG(18:3_35:2)	Triacylglycerols	0.1252	0.09	0.1	0.55	0.1	0
TG(18:3_36:1)	Triacylglycerols	0.1	1.2	0.1446	3.69	0.1	0
TG(18:3_36:2)	Triacylglycerols	0.1258	5.07	0.2852	12.96	0.1	0

Metabolite	Class	Stool LOD	Mean	Plasma LOD	Mean	Urine LOD	Mean
TG(18:3_36:3)	Triacylglycerols	0.1284	7.51	0.1	7.39	0.1	0.01
TG(18:3_36:4)	Triacylglycerols	0.1	10.97	0.1716	2.91	0.1	0
TG(18:3_38:5)	Triacylglycerols	0.135	0.03	0.141	0.51	0.1	0.01
TG(18:3_38:6)	Triacylglycerols	0.1	0.02	0.1096	0.27	0.1	0
TG(20:0_32:3)	Triacylglycerols	0.1	0.06	0.1	0.59	0.1	0
TG(20:0_32:4)	Triacylglycerols	0.1	0.19	0.1	0.51	0.1	0
TG(20:0_34:1)	Triacylglycerols	0.3894	0.33	2.1416	2.13	0.308	0.04
TG(20:1_24:3)	Triacylglycerols	1.996	0.01	1.324	0.3	2.439	0.01
TG(20:1_26:1)	Triacylglycerols	0.277	0.01	0.3554	0.08	0.368	0
TG(20:1_30:1)	Triacylglycerols	0.125	0.01	0.1956	0.23	0.104	0
TG(20:1_31:0)	Triacylglycerols	6.432	0.53	6.93	2.14	1.843	0.86
TG(20:1_32:1)	Triacylglycerols	0.1422	0.01	0.106	1.55	0.138	0.11
TG(20:1_32:2)	Triacylglycerols	0.1174	0.02	0.1272	0.49	0.112	0
TG(20:1_32:3)	Triacylglycerols	0.1	0.02	0.1	0.14	0.100	0
TG(20:1_34:0)	Triacylglycerols	0.1098	0.03	0.105	0.7	0.287	0.07
TG(20:1_34:1)	Triacylglycerols	0.1412	0.26	0.6372	4.05	0.118	0
TG(20:1_34:2)	Triacylglycerols	0.1	0.25	0.1	2.32	0.1	0
TG(20:1_34:3)	Triacylglycerols	0.1	0.07	0.1	0.45	0.1	0
TG(20:2_32:0)	Triacylglycerols	0.45	0.02	0.45	0.87	0.45	0.01
TG(20:2_32:1)	Triacylglycerols	0.1	0.02	0.1	1.41	0.1	0.01
TG(20:2_34:1)	Triacylglycerols	0.1	0.03	0.1148	3.88	0.1	0
TG(20:2_34:2)	Triacylglycerols	0.1	0.11	0.1	1.98	0.1	0
TG(20:2_34:3)	Triacylglycerols	0.138	0.12	0.1	0.39	0.1	0
TG(20:2_34:4)	Triacylglycerols	0.226	0.1	0.1	0.06	0.1	0
TG(20:2_36:5)	Triacylglycerols	0.115	0.01	0.1	0.05	0.1	0
TG(20:3_32:0)	Triacylglycerols	0.1478	0.01	0.136	2.21	0.136	0
TG(20:3_32:1)	Triacylglycerols	0.1122	0.04	0.1	2.19	0.1	0
TG(20:3_32:2)	Triacylglycerols	0.1384	0.06	0.1	0.55	0.1	0
TG(20:3_34:0)	Triacylglycerols	0.145	0.01	0.2376	1.02	0.1	0.01
TG(20:3_34:1)	Triacylglycerols	0.1	0.03	0.192	8.14	0.1	0.01
TG(20:3_34:2)	Triacylglycerols	0.1264	0.1	0.1	4.2	0.1	0
TG(20:3_34:3)	Triacylglycerols	0.175	0.11	0.1	0.84	0.1	0
TG(20:3_36:3)	Triacylglycerols	0.1	0.02	0.1	1.96	0.1	0
TG(20:3_36:4)	Triacylglycerols	0.1	0.02	0.1	0.83	0.1	0
TG(20:3_36:5)	Triacylglycerols	0.1	0.01	0.1	0.16	0.1	0
TG(20:4_30:0)	Triacylglycerols	0.1	0.01	0.1	1.83	0.1	0
TG(20:4_32:0)	Triacylglycerols	0.1174	0.01	0.1	5.04	0.1	0
TG(20:4_32:1)	Triacylglycerols	0.1	0.02	0.1	4.65	0.1	0
TG(20:4_32:2)	Triacylglycerols	0.1	0.03	0.1	1.18	0.1	0
TG(20:4_33:2)	Triacylglycerols	0.1398	0.02	0.1	0.13	0.1	0
TG(20:4_34:0)	Triacylglycerols	0.1	0.01	0.1	3.21	0.1	0
TG(20:4_34:1)	Triacylglycerols	0.1	0.03	0.1446	19.33	0.1	0.01
TG(20:4_34:2)	Triacylglycerols	0.1796	0.03	0.1	9.56	0.1	0.01
TG(20:4_34:3)	Triacylglycerols	0.1	0.03	0.1	1.55	0.1	0
TG(20:4_35:3)	Triacylglycerols	0.1168	0.01	0.1	0.04	0.1	0
TG(20:4_36:2)	Triacylglycerols	0.1	0.03	0.3418	10.2	0.1	0.01
TG(20:4_36:3)	Triacylglycerols	0.1	0.02	0.1	5.44	0.1	0
TG(20:4_36:4)	Triacylglycerols	0.1	0.01	0.1	2.09	0.1	0
TG(20:4_36:5)	Triacylglycerols	0.1	0.01	0.1	0.58	0.1	0
TG(20:5_34:0)	Triacylglycerols	0.1	0.01	0.1	0.78	0.123	0.01
TG(20:5_34:1)	Triacylglycerols	0.121	0.04	0.1228	4.87	0.1	0.02

Metabolite	Class	Stool LOD	Stool Mean	Plasma LOD	Plasma Mean	Urine LOD	Urine Mean
TG(20:5_34:2)	Triacylglycerols	0.1	0.04	0.1404	2.63	0.167	0.04
TG(20:5_36:2)	Triacylglycerols	0.1	0.07	0.1	2.77	0.1	0.01
TG(20:5_36:3)	Triacylglycerols	0.1	0.11	0.1	1.77	0.1	0
TG(22:0_32:4)	Triacylglycerols	0.1	0.01	0.1	0.02	0.1	0
TG(22:1_32:5)	Triacylglycerols	0.1014	0.04	0.0944	0.03	0.1	0
TG(22:2_32:4)	Triacylglycerols	0.1258	0.24	0.1524	0.09	0.1	0.03
TG(22:3_30:2)	Triacylglycerols	0.1	0.01	0.1	0.02	0.1	0
TG(22:4_32:0)	Triacylglycerols	0.1	0	0.1	0.53	0.1	0
TG(22:4_32:2)	Triacylglycerols	0.1	0.01	0.1	0.05	0.1	0
TG(22:4_34:2)	Triacylglycerols	0.1	0.02	0.1	1.18	0.1	0
TG(22:5_32:0)	Triacylglycerols	0.1	0.02	0.126	1.48	0.313	0.12
TG(22:5_32:1)	Triacylglycerols	0.1	0.01	0.1	1.45	0.1	0.02
TG(22:5_34:1)	Triacylglycerols	0.1	0.04	0.2802	7.56	0.1	0.03
TG(22:5_34:2)	Triacylglycerols	0.1	0.03	0.1	3.51	0.1	0
TG(22:5_34:3)	Triacylglycerols	0.1	0.01	0.1	0.61	0.1	0
TG(22:6_32:0)	Triacylglycerols	0.1	0.01	0.1	2.71	0.157	0.06
TG(22:6_32:1)	Triacylglycerols	0.1	0.01	0.1	2.76	0.153	0.08
TG(22:6_34:1)	Triacylglycerols	0.1136	0.02	0.4024	11.57	0.1	0
TG(22:6_34:2)	Triacylglycerols	0.1	0.02	0.1054	6.54	0.1	0
TG(22:6_34:3)	Triacylglycerols	0.1	0.01	0.1	0.92	0.1	0
Choline	Vitamins & Cofactors	0.1028	7.15	0.0994	6.77	0.583	13.63

Metabolites reliably detected in individuals without advanced colorectal neoplasms >LOD are marked in green; Abbreviations: 3-IAA, 3-Indoleacetic acid; 3-IPA, 3-Indolepropionic acid; 5-AVA, 5-Aminovaleric acid; AABA, alpha-Aminobutyric acid; Abs Acid, Abscisic acid; AconAcid, Aconitic acid; Ac-Orn, Acetylornithine; ADMA, Asymmetric dimethylarginine; Ala, Alanine; alpha-AAA, alpha Aminoadipic acid; Arg, Arginine; Asn, asparagine; Asp, aspartate; BABA, beta-Aminobutyric acid; betaAla, beta-Alanine; C0, Carnitine; C7-DC, Pimeloylcarnitine; C8, Octanoylcarnitine; C9, Nonaylcarnitine; C10, Decanoylcarnitine; C10:1, Decenoylcarnitine; C10:2, Decadienoylcarnitine; C12, Dodecanoylcarnitine; C16, Hexadecanoylcarnitine; C16-1OH, Hydroxyhexadecanoylcarnitine; C16:2, Hexadecadienoylcarnitine; C18, Octadecanoylcarnitine; CA, Cholic acid; CDCA, Chenodeoxycholic acid; CE, Cholesteryl ester; Cer, Ceramide; Cit, Citrulline; Cys, Cysteine; DCA, Deoxycholic acid; DG, Diglycerides; DHA, Docosahexaenoic acid, DHEAS, Dehydroepiandrosteron sulfate; DiCA(12:0), Dodecanedioic acid; DOPA, Dihydroxyphenylalanine; EPA, Eicosapentaenoic acid; FA, Fatty acid; Fa(12:0), Lauric acid; FA(14:0), Myristic acid; FA(18:1), Octadecenoic acid; FA(18:2), Octdecadienoic acid; FA(20:1), Eicosenoic acid; FA(20:2), Eicosadienoic acid; FA(20:3), Eicosatrienoic acid; H1, Hexoses (including glucose); GABA, gamma-Aminobutyric acid; GCA, Glycocholic acid; GDCA, Glycodeoxycholic acid; GLCA, Glycolithocholic acid; GLCAS, Glycolithocholic acid sulfate; Gln, Glutamine; Glu, Glutamate; Gly, Glycine; GUDCAS, Glycoursodeoxycholic aicd; HArg, Homoarginine; HCys, Homocysteine; HexCer, Hexosylceramide; Hex2Cer, Dihexosylceramide; Hex3Cer, Trihexosylceramide; His, Histidine; Ile, Isoleucine; Leu, Leucine; lysoPC, Lysophosphatidylcholine; Met, Methionine; Met-So, Methionine sulfoxide; OH-GlutAcid, 3-Hydroxyglutaric acid; Orn, Ornithine; PC, Phosphatidylcholine; p-cresol SO4, p-Cresol sulfate; PEA, Phenylethylamine; Phe, Phenylalanine; Pro, Proline; SDMA, Symmetric dimethylarginine; Ser, Serine; SM, Sphingomyelin; Suc, Succinic acid; t4-OH-Pro, trans-4-Hydroxyproline; TCA, Taurocholic acid; TCDCA, Taurochenodeoxycholic acid; TDCA, Taurodeoxycholic acid; TG, Triglyceride; Thr, Threonine; TLCA, Taurolithocholic acid; TMAO, Trimethylamine N-oxide; TMCA, Tauromurocholic acid; Trp, Tryptophan; Tyr, Tyrosine; Val, Valine.

Table S3. Mean Concentrations (+SD) of metabolites for participants with and without advanced colorectal neoplasms for each of the bio-fluids (stool, plasma, urine).

Metabolite	Metabolite Class	Stool (no neo.) Mean (±SD)	Stool (ACN) Mean (±SD)	P Value	Blood (no neo.) Mean (±SD)	Blood (ACN) Mean (±SD)	P Value	Urine (no neo.) Mean (±SD)	Urine (ACN) Mean (±SD)	P Value
C0	Acylcarnitines	13.14 (±16.59)	11.77 (±11.37)	0.17	37.79 (±14.77)	36.3 (±15.46)	0.17	4.6 (±4.2)	4.87 (±4.6)	0.94
C2	Acylcarnitines	0.6 (±2.02)	0.53 (±0.61)	0.52	5.8 (±2.83)	6.56 (±3.15)	0.01	2.89 (±6.7)	3.52 (±4.83)	0.05
C3	Acylcarnitines	0.37 (±0.16)	0.4 (±0.22)	0.28	0.42 (±0.14)	0.4 (±0.13)	0.20	0.18 (±0.14)	0.17 (±0.14)	0.66
C3:1	Acylcarnitines	0.22 (±0.11)	0.23(±0.12)	0.51	0.03(±0.01)	0.03(±0.01)	0.67	0.01(±0.01)	0.01(±0.01)	0.27
C3-DC (C4-OH)	Acylcarnitines	0.44 (±0.36)	0.53(±0.53)	0.29	0.06(±0.04)	0.06(±0.05)	0.54	0.02(±0.09)	0.02(±0.03)	0.01
C3-OH	Acylcarnitines	0.37 (±0.27)	0.37(±0.28)	0.69	0.04(±0.01)	0.04(±0.01)	0.83	0.02(±0.01)	0.02(±0.01)	0.28
C4	Acylcarnitines	0.25 (±0.18)	0.25(±0.17)	0.71	0.19(±0.07)	0.19(±0.08)	0.64	0.88(±0.46)	0.72(±0.39)	0.00
C4:1	Acylcarnitines	0.19 (±0.14)	0.19(±0.15)	0.89	0.02(±0.01)	0.02(±0.01)	0.39	0.02(±0.02)	0.03(±0.01)	0.048
C5	Acylcarnitines	0.59 (±0.46)	0.59(±0.43)	0.76	0.17(±0.09)	0.18(±0.09)	0.95	0.37(±0.23)	0.33(±0.23)	0.02
C5:1	Acylcarnitines	0.27 (±0.17)	0.29(±0.18)	0.19	0.04(±0.01)	0.04(±0.01)	0.48	0.08(±0.03)	0.08(±0.03)	0.03
C5:1-DC	Acylcarnitines	0.21 (±0.2)	0.21(±0.19)	0.57	0.01(±0.01)	0.01(±0.01)	0.78	0.02(±0.02)	0.02(±0.02)	0.95
C5-DC (C6-OH)	Acylcarnitines	0.35 (±0.26)	0.35(±0.26)	0.51	0.05(±0.01)	0.05(±0.02)	0.49	0.08(±0.03)	0.07(±0.03)	0.62
C5-M-DC	Acylcarnitines	0.19 (±0.17)	0.18(±0.17)	0.55	0.01(±0)	0.01(±0.01)	0.91	0.02(±0.02)	0.03(±0.07)	0.15
C5-OH (C3-DC-M)	Acylcarnitines	0.51 (±0.67)	0.49(±0.46)	0.68	0.05(±0.02)	0.06(±0.02)	0.33	0.21(±0.06)	0.19(±0.06)	0.01
C6 (C4:1-DC)	Acylcarnitines	0.33 (±0.16)	0.35(±0.18)	0.47	0.11(±0.03)	0.12(±0.05)	0.56	0.1(±0.12)	0.09(±0.12)	0.02
C6:1	Acylcarnitines	0.18 (±0.12)	0.19(±0.13)	0.32	0.04(±0.01)	0.04(±0.02)	0.18	0.01(±0.01)	0.01(±0.01)	0.43
C7-DC	Acylcarnitines	0.26 (±0.2)	0.25(±0.21)	0.61	0.06(±0.04)	0.06(±0.03)	0.91	0.02(±0.02)	0.03(±0.02)	0.58
C8	Acylcarnitines	0.63 (±0.42)	0.65(±0.46)	0.88	0.2(±0.09)	0.22(±0.12)	0.02	0.1(±0.04)	0.1(±0.04)	0.47
C9	Acylcarnitines	0.15 (±0.13)	0.15(±0.15)	0.80	0.07(±0.03)	0.07(±0.03)	0.37	0.25(±0.19)	0.22(±0.2)	0.003
C10	Acylcarnitines	0.66 (±0.44)	0.69(±0.49)	0.58	0.29(±0.15)	0.33(±0.17)	0.01	0.07(±0.11)	0.06(±0.03)	0.10
C10:1	Acylcarnitines	0.33 (±0.16)	0.35(±0.24)	0.62	0.15(±0.04)	0.16(±0.05)	0.06	0.13(±0.11)	0.13(±0.11)	0.45
C10:2	Acylcarnitines	0.59 (±0.22)	0.6(±0.26)	0.71	0.21(±0.09)	0.21(±0.09)	0.52	0.42(±0.59)	0.48(±0.87)	0.17
C12	Acylcarnitines	0.41 (±0.27)	0.41(±0.3)	0.68	0.11(±0.04)	0.12(±0.05)	0.01	0.05(±0.03)	0.05(±0.03)	0.95
C12:1	Acylcarnitines	0.49 (±1.51)	0.41(±0.35)	0.89	0.09(±0.03)	0.1(±0.04)	0.01	0.06(±0.06)	0.05(±0.06)	0.31
C12-DC	Acylcarnitines	1.27 (±0.56)	1.25(±0.7)	0.38	0.39(±0.08)	0.37(±0.08)	0.09	0.08(±0.07)	0.07(±0.07)	0.08
C14	Acylcarnitines	0.51 (±0.32)	0.46(±0.27)	0.13	0.06(±0.02)	0.06(±0.02)	0.16	0.02(±0.02)	0.02(±0.02)	0.52
C14:1	Acylcarnitines	0.15 (±0.09)	0.14(±0.09)	0.58	0.06(±0.04)	0.07(±0.05)	0.03	0.01(±0.01)	0.01(±0.01)	0.55
C14:1-OH	Acylcarnitines	0.13 (±0.08)	0.14(±0.09)	0.90	0.01(±0.01)	0.02(±0.01)	0.02	0.01(±0.01)	0.01(±0.01)	0.42
C14:2	Acylcarnitines	0.19 (±0.07)	0.19(±0.08)	0.63	0.05(±0.01)	0.06(±0.02)	0.03	0.01(±0.01)	0.01(±0.01)	0.30
C14:2-OH	Acylcarnitines	0.12 (±0.06)	0.13(±0.07)	0.63	0.02(±0.01)	0.02(±0.01)	0.98	0.01(±0.01)	0.01(±0.01)	0.08

Metabolite	Metabolite Class	Stool (no neo.) Mean (±SD)	Stool (ACN) Mean (±SD)	P Value	Blood (no neo.) Mean (±SD)	Blood (ACN) Mean (±SD)	P Value	Urine (no neo.) Mean (±SD)	Urine (ACN) Mean (±SD)	P Value
C14:2-OH	Acylcarnitines	0.12 (±0.06)	0.13(±0.07)	0.63	0.02(±0.01)	0.02(±0.01)	0.98	0.01(±0.01)	0.01(±0.01)	0.08
C16	Acylcarnitines	0.69 (±0.99)	0.71(±0.73)	0.71	0.13(±0.03)	0.13(±0.03)	0.02	0.02(±0.04)	0.02(±0.06)	0.14
C16-OH	Acylcarnitines	0.22 (±0.3)	0.2(±0.13)	0.98	0.02(±0)	0.02(±0.01)	0.11	0.01(±0.02)	0.01(±0.02)	0.34
C16:1	Acylcarnitines	0.19 (±0.16)	0.18(±0.11)	0.72	0.03(±0.01)	0.04(±0.02)	0.002	0.01(±0.01)	0.01(±0.01)	0.14
C16:1-OH	Acylcarnitines	0.13 (±0.11)	0.12(±0.08)	0.84	0.01(±0.01)	0.01(±0.01)	0.84	0.01(±0.01)	0(±0.01)	0.37
C16:2	Acylcarnitines	0.18 (±0.09)	0.18(±0.1)	0.42	0.01(±0)	0.02(±0.01)	0.10	0.01(±0.01)	0.01(±0.01)	0.28
C16:2-OH	Acylcarnitines	0.14 (±0.06)	0.15(±0.06)	0.57	0.02(±0)	0.02(±0.01)	0.85	0.01(±0.01)	0.01(±0.01)	0.08
C18	Acylcarnitines	0.51 (±0.42)	0.53(±0.39)	0.32	0.05(±0.01)	0.05(±0.01)	0.17	0.01(±0.03)	0.01(±0.01)	0.11
C18:1	Acylcarnitines	0.38 (±0.44)	0.45(±0.56)	0.45	0.14(±0.05)	0.16(±0.05)	0.07	0.01(±0.01)	0.01(±0.01)	0.02
C18:1-OH	Acylcarnitines	0.21 (±0.36)	0.2(±0.17)	0.81	0.01(±0)	0.01(±0)	0.17	0.01(±0.01)	0.01(±0.01)	0.04
C18:2	Acylcarnitines	0.19 (±0.19)	0.2(±0.16)	0.69	0.06(±0.02)	0.06(±0.02)	0.74	0.01(±0.06)	0.01(±0.03)	0.04
Trigonelline	Alkaloids	6.06 (±14.44)	4.15(±4.03)	0.52	2.74(±2.71)	2.48(±2.4)	0.38	18.73(±17.61)	15.84(±15.87)	0.07
TMAO	Amine Oxides	0.79 (±3.36)	1.28(±9.26)	0.83	5.15(±6.58)	4.82(±4.31)	0.49	50.37(±71.95)	43.93(±41.19)	0.30
Ala	Aminoacids	1375.45 (±899.66)	1348(±1091.08)	0.41	383.3(±81.37)	373.47(±90.51)	0.20	27.19(±13.09)	23.36(±14.6)	<.0001
Arg	Aminoacids	362.88 (±245.9)	340.44(±246.03)	0.34	54.34(±18.22)	54.17(±18.02)	0.65	2.5(±1.46)	2.23(±1.65)	0.004
Asn	Aminoacids	55.71 (±74.95)	50.73(±77.13)	0.11	44.91(±10.84)	42.54(±8.14)	0.03	6.55(±6.12)	7.43(±14.52)	0.41
Asp	Aminoacids	930.09 (±485.57)	909.53(±459.68)	0.64	2.91(±1.21)	3.11(±1.17)	0.049	0.38(±0.31)	0.38(±0.48)	0.10
Cys	Aminoacids	34.1 (±25.34)	38.22(±51.74)	0.60	72.88(±13.48)	75.7(±14.27)	0.03	21.29(±11.82)	20.14(±8.02)	0.36
Gln	Aminoacids	176.1 (±133.51)	169.24(±138.55)	0.27	561.56(±69.51)	551.37(±75.2)	0.16	40.19(±15.33)	34.81(±14.85)	0.0002
Glu	Aminoacids	3417.62 (±1713.85)	3450.77(±1657.5)	0.82	47.6(±21.69)	53.19(±23.7)	0.01	1.99(±1.63)	1.83(±1.31)	0.05
Gly	Aminoacids	484.32 (±447.02)	471.23(±524.93)	0.25	215.87(±67.95)	209.82(±66.4)	0.24	90.2(±54.93)	86.76(±102.03)	0.005
His	Aminoacids	53.38 (±157.68)	48.34(±79.5)	0.97	75.76(±12.42)	72.85(±10.85)	0.06	40.76(±21.6)	34.22(±18.01)	0.003
Ile	Aminoacids	310.29 (±344.3)	257.62(±281.59)	0.04	73.25(±25.77)	70.55(±22.67)	0.54	0.97(±0.38)	0.92(±0.42)	0.06
Leu	Aminoacids	614.74 (±496.54)	550.03(±453.26)	0.11	127.33(±39.39)	124.35(±34.26)	0.73	2.47(±0.95)	2.33(±0.87)	0.06
Lys	Aminoacids	774.13 (±310.79)	787.28(±373.83)	0.83	175.77(±21.63)	175.64(±23.82)	0.85	11.25(±7.85)	9.69(±5.69)	0.04
Met	Aminoacids	232.32 (±161.58)	216.74(±161.77)	0.17	22.62(±6.68)	21.6(±5.72)	0.24	0.85(±0.36)	0.84(±0.72)	0.01
Phe	Aminoacids	253.1 (±268.39)	228.22(±241.22)	0.14	58.55(±12.07)	58.03(±10.64)	0.97	4.16(±1.56)	3.85(±1.58)	0.02
Pro	Aminoacids	313.34 (±167.35)	298.26(±131.46)	0.75	211.05(±62.8)	203.3(±85.82)	0.046	2.09(±12.7)	2.48(±13.31)	0.15
Ser	Aminoacids	295.39 (±157.4)	300.06(±175.68)	0.88	95.3(±23.35)	92.27(±20.44)	0.30	23.03(±8.7)	20.38(±8.23)	0.001
Thr	Aminoacids	191.64 (±130.7)	187.9(±185.38)	0.12	111.3(±25.47)	107.08(±27.39)	0.04	8.55(±4.45)	7.28(±4.51)	0.0002
Trp	Aminoacids	43.39 (±41.2)	36.95(±31.46)	0.20	55.11(±9.91)	53.24(±10.21)	0.07	6.14(±2.26)	5.56(±2.19)	0.01
Tyr	Aminoacids	282.83 (±226.97)	257.98(±220.33)	0.12	67.59(±18)	65.01(±17.67)	0.17	7.64(±3.39)	6.7(±3.13)	0.002

Metabolite	Metabolite Class	Stool (no neo.) Mean (±SD)	Stool (ACN) Mean (±SD)	P Value	Blood (no neo.) Mean (±SD)	Blood (ACN) Mean (±SD)	P Value	Urine (no neo.) Mean (±SD)	Urine (ACN) Mean (±SD)	P Value
Val	Aminoacids	450.1 (±417.36)	411.32(±369.19)	0.27	204.78(±39.78)	200.27(±40.26)	0.29	4.2(±1.48)	3.92(±1.69)	0.01
1-Met-His	Aminoacids Related	3.31 (±7.8)	2.93(±5.81)	0.48	5.04(±1.69)	5.52(±2.48)	0.06	16.33(±4.26)	15.84(±5.21)	0.14
3-Met-His	Aminoacids Related	11.15 (±26.32)	7.83(±15.02)	0.52	5.72(±9.92)	5.09(±7.88)	0.20	16.13(±16.83)	14.94(±17.43)	0.61
5-AVA	Aminoacids Related	378.36 (±759.71)	317.67(±540.49)	0.90	0.04(±0.03)	0.04(±0.02)	0.48	0.13(±0.11)	0.12(±0.1)	0.03
AABA	Aminoacids Related	47.51 (±115.49)	45.78(±87.44)	0.39	15.42(±5.34)	16.65(±6.29)	0.15	0.94(±0.45)	0.87(±0.47)	0.02
Ac-Orn	Aminoacids Related	1.82 (±1.73)	2.87(±9.79)	0.72	7.79(±1.7)	8.08(±2.23)	0.80	0.06(±0.04)	0.06(±0.07)	0.47
ADMA	Aminoacids Related	1.11 (±1.03)	0.97(±0.71)	0.77	0.44(±0.07)	0.45(±0.08)	0.54	2.9(±0.73)	2.75(±0.66)	0.0049
alpha-AAA	Aminoacids Related	5.17 (±5.75)	6.01(±7.01)	0.10	1.02(±0.55)	1.04(±0.56)	0.57	2.94(±1.52)	2.51(±1.66)	0.001
Anserine	Aminoacids Related	4.07 (±11.02)	2.34(±4.37)	0.14	0.01(±0.03)	0.02(±0.07)	0.07	1.49(±4.95)	0.76(±1.94)	0.14
BABA	Aminoacids Related	1.77 (±1.81)	2.39(±6)	0.06	0.05(±0.02)	0.06(±0.04)	0.04	0.6(±0.42)	0.66(±0.58)	0.97
Betaine	Aminoacids Related	2.84 (±8.9)	7.64(±66.03)	0.46	33.52(±9.51)	33.59(±10.87)	0.91	12.76(±11.23)	17.78(±36.53)	0.70
c4-OH-Pro	Aminoacids Related	0.83 (±0.97)	0.8(±0.69)	0.89	0.08(±0.06)	0.08(±0.06)	0.42	0.02(±0.03)	0.03(±0.08)	0.13
Carnosine	Aminoacids Related	5.08 (±30.79)	4.61(±22.18)	0.52	0.04(±0.05)	0.05(±0.06)	0.27	1.86(±3.63)	1.55(±2.29)	0.44
Cit	Aminoacids Related	271.19 (±170.22)	286.15(±188.58)	0.74	31.55(±9.27)	31.03(±10.92)	0.15	0.39(±0.3)	0.38(±0.47)	0.22
Creatinine	Aminoacids Related	60.19 (±149.67)	60.26(±193.19)	0.53	70.05(±14.63)	73.76(±17.3)	0.04	n.a.(±n.a.)	n.a.(±n.a.)	n.a.
Cystine	Aminoacids Related	1.61 (±3.02)	1.44(±3.48)	0.38	71.89(±18.95)	79.45(±21.79)	0.0002	15.19(±8.93)	15.73(±8.61)	0.32
DOPA	Aminoacids Related	0.32 (±0.59)	0.28(±0.37)	0.05	0.01(±0.09)	0(±0.01)	0.06	0.02(±0.11)	0.02(±0.06)	0.15
HArg	Aminoacids Related	0.24 (±0.48)	0.2(±0.25)	0.83	2.15(±0.73)	2.07(±0.81)	0.25	0.15(±0.18)	0.11(±0.12)	0.04
HCys	Aminoacids Related	12.6 (±8.94)	11.75(±7.25)	0.53	6.93(±2.02)	7.13(±2.25)	0.61	4.8(±2.12)	4.98(±6.5)	0.06
Kynurenine	Aminoacids Related	0.39 (±0.31)	0.42(±0.32)	0.43	2.09(±0.52)	2.17(±0.71)	0.29	0.25(±0.18)	0.27(±0.19)	0.79
Met-SO	Aminoacids Related	23.75 (±17.96)	21.45(±14.34)	0.28	0.75(±0.43)	0.65(±0.25)	0.04	0.17(±0.15)	0.16(±0.26)	0.01
Nitro-Tyr	Aminoacids Related	0.36 (±0.16)	0.36(±0.14)	0.59	0.02(±0.02)	0.02(±0.02)	0.90	0.01(±0.03)	0.02(±0.08)	0.06
Orn	Aminoacids Related	48.31 (±61.69)	49.09(±61.65)	0.39	102.3(±27.19)	101.83(±32.02)	0.56	1.25(±0.6)	1.21(±0.92)	0.15
PAG	Aminoacids Related	0.06 (±0.2)	0.04(±0.09)	0.75	0.01(±0.01)	0.01(±0.01)	0.20	0.23(±0.24)	0.18(±0.21)	0.01
PheAlaBetaine	Aminoacids Related	0.19 (±0.55)	0.26(±0.83)	0.12	0.01(±0.01)	0.01(±0.01)	0.42	0(±0)	0(±0.01)	0.11
ProBetaine	Aminoacids Related	13.14 (±19.31)	14.11(±20.6)	0.66	7.54(±10.84)	6.64(±10.03)	0.03	20.22(±38.34)	16.38(±24.72)	0.03
Sarcosine	Aminoacids Related	11.3 (±28.7)	9.46(±9.75)	0.91	2.56(±0.64)	2.53(±0.77)	0.24	0.19(±0.12)	0.2(±0.18)	0.87
SDMA	Aminoacids Related	1.44 (±1.96)	1.29(±1.9)	0.68	0.54(±0.11)	0.57(±0.14)	0.03	3.52(±0.78)	3.44(±0.63)	0.45
t4-OH-Pro	Aminoacids Related	10.83 (±16.34)	9.6(±9.71)	0.76	9.99(±6.11)	9.84(±6)	0.76	0.29(±0.36)	0.66(±3.68)	0.25
Taurine	Aminoacids Related	167.67 (±312.36)	163.21(±329.03)	0.71	43.79(±10.32)	44.92(±12.1)	0.47	28.73(±19.22)	26.72(±22.2)	0.14
TrpBetaine	Aminoacids Related	0.4 (±0.97)	0.4(±0.81)	0.53	0.46(±0.6)	0.36(±0.52)	0.002	0.1(±0.13)	0.07(±0.1)	0.0002
CA	Bile Acids	73.8(±209.84)	119.59(±406.77)	0.34	0.22(±0.56)	0.17(±0.39)	0.10	0.01(±0.02)	0(±0.01)	0.28

Metabolite	Metabolite Class	Stool (no neo.) Mean (±SD)	Stool (ACN) Mean (±SD)	P Value	Blood (no neo.) Mean (±SD)	Blood (ACN) Mean (±SD)	P Value	Urine (no neo.) Mean (±SD)	Urine (ACN) Mean (±SD)	P Value
CDCA	Bile Acids	58.05(±145.68)	74.73(±213.7)	0.55	0.41(±1)	0.32(±0.62)	0.39	0(±0)	0(±0)	0.33
DCA	Bile Acids	167.34(±91.91)	185.07(±82.91)	0.03	0.33(±0.34)	0.34(±0.43)	0.59	0.04(±0.03)	0.04(±0.02)	0.03
GCA	Bile Acids	1.87(±3.95)	2.15(±5.61)	0.11	0.31(±0.45)	0.31(±0.38)	0.82	0.01(±0.01)	0.01(±0.01)	0.24
GCDCA	Bile Acids	6.82(±17.87)	7.63(±19.51)	0.63	1.2(±1.01)	1.14(±0.91)	0.46	0(±0)	0(±0)	0.31
GDCA	Bile Acids	8.11(±31.04)	8.58(±20.19)	0.02	0.52(±0.55)	0.55(±0.54)	0.81	0(±0)	0(±0)	0.13
GLCA	Bile Acids	0.31(±0.34)	0.37(±0.42)	0.12	0.03(±0.03)	0.03(±0.03)	0.05	0(±0)	0(±0)	0.25
GLCAS	Bile Acids	1.48(±4.34)	2.01(±5.12)	0.08	0.28(±0.25)	0.29(±0.24)	0.34	0.06(±0.07)	0.08(±0.08)	0.07
GUDCA	Bile Acids	0.24(±0.5)	0.18(±0.38)	0.04	0.11(±0.19)	0.09(±0.12)	0.09	0(±0)	0(±0)	0.94
TCA	Bile Acids	2.35(±7.65)	2.98(±8.66)	0.59	0.07(±0.15)	0.07(±0.13)	0.48	0(±0)	0(±0)	0.23
TCDCA	Bile Acids	1.66(±4.5)	1.9(±5.08)	0.53	0.15(±0.2)	0.15(±0.18)	0.88	0(±0)	0(±0)	0.41
TDCA	Bile Acids	3.33(±18)	3.68(±12.33)	0.48	0.07(±0.1)	0.07(±0.11)	0.61	0(±0)	0(±0)	0.21
TLCA	Bile Acids	0.63(±1.51)	0.54(±0.98)	0.30	0.01(±0.01)	0.01(±0.01)	0.09	0(±0)	0(±0)	0.48
TMCA	Bile Acids	0.1(±0.3)	0.3(±1.92)	0.47	0.01(±0.01)	0.01(±0.01)	0.26	0(±0)	0(±0.01)	0.66
beta-Ala	Biogenic Amines	49.58(±105.73)	64.91(±162.66)	0.18	3.96(±4.58)	3.68(±3.37)	0.22	1.71(±3.77)	1.3(±3.27)	0.41
Dopamine	Biogenic Amines	0.79(±1.6)	0.58(±0.58)	0.33	0.03(±0.05)	0.03(±0.05)	0.93	0.18(±0.52)	0.14(±0.12)	0.29
GABA	Biogenic Amines	44.77(±106.71)	32.37(±55.46)	0.60	0.16(±0.03)	0.16(±0.03)	0.52	0.16(±0.11)	0.14(±0.07)	0.02
Histamine	Biogenic Amines	15.66(±53.85)	17.92(±59.64)	0.52	0.12(±0.07)	0.11(±0.07)	0.44	0.03(±0.04)	0.04(±0.08)	0.06
PEA	Biogenic Amines	1.44(±7.14)	0.86(±2.19)	0.16	0(±0)	0(±0)	1.00	0.01(±0.01)	0.01(±0.01)	0.73
Putrescine	Biogenic Amines	34.04(±43.3)	35.99(±40.91)	0.24	0.1(±0.03)	0.11(±0.04)	0.004	0.1(±0.25)	0.09(±0.17)	0.70
Serotonin	Biogenic Amines	2.24(±3.27)	2.03(±3.11)	0.33	0.04(±0.04)	0.05(±0.07)	0.28	0.04(±0.02)	0.05(±0.03)	0.18
Spermidine	Biogenic Amines	43.64(±28.01)	47.56(±30.76)	0.25	0.18(±0.04)	0.18(±0.05)	0.28	0.02(±0.02)	0.03(±0.03)	0.14
Spermine	Biogenic Amines	0.94(±0.55)	1.1(±1.41)	0.54	0.14(±0.03)	0.14(±0.03)	0.32	0.11(±0.21)	0.11(±0.23)	0.61
AconAcid	Carboxylic Acids	5.28(±4.84)	4.96(±4.16)	0.20	0(±0)	0(±0)	0.54	40.65(±23.11)	39.61(±21.51)	0.84
DiCA(12:0)	Carboxylic Acids	3.34(±6.27)	4.07(±8.3)	0.24	0.12(±0.07)	0.13(±0.1)	0.82	0.04(±0.04)	0.04(±0.05)	0.16
DiCA(14:0)	Carboxylic Acids	0.18(±0.11)	0.18(±0.14)	0.78	0.06(±0.06)	0.07(±0.08)	0.32	0(±0.01)	0(±0.01)	0.11
HipAcid	Carboxylic Acids	13.09(±47.33)	11.96(±50.97)	0.77	9.76(±8.93)	8.27(±10.14)	0.001	216.94(±162.4)	173.08(±140.47)	0.001
Lac	Carboxylic Acids	295.34(±1005.43)	208.16(±318.39)	0.97	3868.24 (±1264.95)	4011.62 (±1170.57)	0.11	30.52(±39.8)	28.04(±33.3)	0.07
OH-GlutAcid	Carboxylic Acids	8.94(±13.26)	9.23(±14.19)	0.96	0.79(±0.2)	0.82(±0.21)	0.47	1.67(±0.69)	1.61(±0.8)	0.17
Suc	Carboxylic Acids	670.65(±1747.86)	569.72(±1512.8)	0.98	3.55(±3.28)	3.4(±2.98)	0.58	1.88(±1.93)	1.63(±0.89)	0.04
Cer(d16:1/18:0)	Ceramides	0.3(±0.31)	0.31(±0.24)	0.23	0.06(±0.05)	0.07(±0.05)	0.35	0(±0)	0(±0)	0.62
Cer(d16:1/20:0)	Ceramides	0.1(±0.1)	0.1(±0.11)	0.92	0.05(±0.04)	0.04(±0.04)	0.14	0(±0)	0(±0)	0.76

Metabolite	Metabolite Class	Stool (no neo.) Mean (±SD)	Stool (ACN) Mean (±SD)	P Value	Blood (no neo.) Mean (±SD)	Blood (ACN) Mean (±SD)	P Value	Urine (no neo.) Mean (±SD)	Urine (ACN) Mean (±SD)	P Value
Cer(d16:1/22:0)	Ceramides	0.92(±0.87)	0.89(±1.05)	0.47	0.24(±0.1)	0.23(±0.11)	0.11	0(±0)	0(±0)	0.56
Cer(d16:1/23:0)	Ceramides	0.52(±0.57)	0.51(±0.7)	0.53	0.09(±0.05)	0.08(±0.05)	0.03	0(±0)	0(±0)	0.67
Cer(d16:1/24:0)	Ceramides	0.4(±0.4)	0.4(±0.44)	0.87	0.25(±0.08)	0.24(±0.09)	0.18	0(±0)	0(±0)	0.12
Cer(d18:1/14:0)	Ceramides	0.34(±0.33)	0.34(±0.5)	0.51	0.05(±0.03)	0.05(±0.03)	0.07	0(±0)	0(±0)	0.20
Cer(d18:1/16:0)	Ceramides	2.05(±1.97)	2.29(±2.27)	0.43	0.41(±0.08)	0.42(±0.1)	0.27	0(±0.01)	0(±0)	0.34
Cer(d18:1/18:0)	Ceramides	1.97(±2.67)	1.94(±2.08)	0.18	0.18(±0.05)	0.19(±0.05)	0.13	0(±0)	0(±0)	0.86
Cer(d18:1/18:0(OH))	Ceramides	0.36(±0.37)	0.35(±0.35)	0.75	0(±0.01)	0(±0.01)	0.42	0(±0)	0(±0)	0.13
Cer(d18:1/18:1)	Ceramides	0.79(±1.34)	0.82(±1.05)	0.40	0.02(±0.01)	0.02(±0.01)	0.86	0(±0)	0(±0)	0.02
Cer(d18:1/20:0)	Ceramides	0.24(±0.25)	0.22(±0.21)	0.57	0.13(±0.04)	0.14(±0.06)	0.70	0(±0)	0(±0)	0.02
Cer(d18:1/20:0(OH))	Ceramides	5.02(±4.3)	4.88(±4.16)	0.94	0.76(±0.8)	0.7(±0.81)	0.40	0.02(±0.06)	0.02(±0.05)	0.80
Cer(d18:1/22:0)	Ceramides	1.37(±1.28)	1.42(±1.35)	1.00	0.8(±0.18)	0.83(±0.21)	0.33	0(±0)	0(±0)	0.07
Cer(d18:1/23:0)	Ceramides	1.36(±1.34)	1.36(±1.41)	0.66	1.04(±0.26)	1.05(±0.31)	0.87	0(±0)	0(±0)	0.08
Cer(d18:1/24:0)	Ceramides	1.5(±1.47)	1.62(±1.54)	0.52	2.53(±0.59)	2.6(±0.68)	0.42	0(±0.01)	0(±0.01)	0.69
Cer(d18:1/24:1)	Ceramides	2.09(±1.86)	2.29(±2.36)	0.80	1.25(±0.24)	1.31(±0.34)	0.14	0(±0.02)	0(±0)	0.50
Cer(d18:1/25:0)	Ceramides	1.06(±0.91)	1.04(±0.87)	0.66	0.37(±0.09)	0.36(±0.1)	0.44	0(±0)	0(±0)	0.53
Cer(d18:1/26:0)	Ceramides	0.27(±0.22)	0.26(±0.19)	0.62	0.05(±0.04)	0.05(±0.04)	0.56	0(±0)	0(±0)	0.26
Cer(d18:1/26:1)	Ceramides	0.07(±0.08)	0.08(±0.08)	0.50	0.01(±0.02)	0.01(±0.02)	0.64	0(±0)	0(±0)	0.42
Cer(d18:2/14:0)	Ceramides	0.01(±0.01)	0.01(±0.01)	0.09	0(±0)	0(±0)	0.12	0(±0)	0(±0)	0.13
Cer(d18:2/16:0)	Ceramides	0.28(±0.19)	0.3(±0.19)	0.12	0.08(±0.03)	0.08(±0.03)	0.97	0(±0)	0(±0)	1.00
Cer(d18:2/18:0)	Ceramides	0.19(±0.22)	0.2(±0.18)	0.07	0.04(±0.03)	0.04(±0.03)	0.96	0(±0)	0(±0)	0.10
Cer(d18:2/18:1)	Ceramides	0.05(±0.05)	0.05(±0.04)	0.31	0(±0)	0(±0)	0.61	0(±0)	0(±0)	0.24
Cer(d18:2/20:0)	Ceramides	0.05(±0.08)	0.05(±0.06)	0.18	0.03(±0.03)	0.03(±0.03)	0.75	0(±0)	0(±0)	0.91
Cer(d18:2/22:0)	Ceramides	0.17(±0.18)	0.18(±0.19)	0.58	0.24(±0.06)	0.23(±0.08)	0.13	0(±0)	0(±0)	0.13
Cer(d18:2/23:0)	Ceramides	0.09(±0.1)	0.08(±0.11)	0.58	0.12(±0.05)	0.12(±0.05)	0.10	0(±0)	0(±0)	0.22
Cer(d18:2/24:0)	Ceramides	0.15(±0.17)	0.16(±0.18)	0.49	0.53(±0.14)	0.52(±0.15)	0.52	0(±0)	0(±0)	0.12
Cer(d18:2/24:1)	Ceramides	0.23(±0.18)	0.27(±0.22)	0.22	0.25(±0.05)	0.25(±0.07)	0.40	0(±0)	0(±0)	0.73
CE(14:0)	Cholesterol Esters	1.57(±1.7)	1.93(±3.83)	0.41	31.93(±11.75)	28.89(±12.35)	0.003	0.04(±0.07)	0.03(±0.07)	0.06
CE(14:1)	Cholesterol Esters	0.38(±0.71)	0.41(±0.83)	0.80	1.41(±0.74)	1.22(±0.65)	0.01	0.01(±0.02)	0.01(±0.02)	0.92
CE(15:0)	Cholesterol Esters	1.31(±1.14)	1.49(±1.35)	0.23	10.86(±4.06)	9.88(±4.38)	0.003	0.03(±0.05)	0.03(±0.07)	0.82
CE(15:1)	Cholesterol Esters	0.93(±0.89)	0.85(±0.94)	0.32	0.75(±0.37)	0.67(±0.34)	0.06	0.09(±0.23)	0.09(±0.21)	0.94
CE(16:0)	Cholesterol Esters	4.62(±5.14)	4.9(±7.45)	0.79	256.2(±66.64)	254.64(±83.14)	0.41	0.91(±2.37)	0.9(±2.57)	0.25
CE(16:1)	Cholesterol Esters	0.77(±0.94)	0.9(±1.17)	0.36	93.52(±42.91)	93.59(±44.78)	0.92	0.02(±0.04)	0.03(±0.15)	0.57

Metabolite	Metabolite Class	Stool (no neo.) Mean (±SD)	Stool (ACN) Mean (±SD)	P Value	Blood (no neo.) Mean (±SD)	Blood (ACN) Mean (±SD)	P Value	Urine (no neo.) Mean (±SD)	Urine (ACN) Mean (±SD)	P Value
CE(17:0)	Cholesterol Esters	1.5(±2.14)	1.63(±2.11)	0.43	7.98(±2.71)	7.33(±2.7)	0.01	0.04(±0.09)	0.05(±0.09)	0.97
CE(17:1)	Cholesterol Esters	0.32(±0.47)	0.39(±0.57)	0.30	8.06(±3.3)	7.81(±3.49)	0.20	0.01(±0.04)	0.01(±0.04)	0.43
CE(18:0)	Cholesterol Esters	1.57(±1.58)	1.74(±2.17)	0.73	20.6(±5.91)	20.57(±6.62)	0.81	0.52(±1.4)	0.52(±1.39)	0.72
CE(18:1)	Cholesterol Esters	9.01(±10.18)	11.94(±15.98)	0.11	508.39(±141.61)	502.99(±155.71)	0.61	0.07(±0.15)	0.11(±0.92)	0.14
CE(18:2)	Cholesterol Esters	9.32(±18.99)	9.92(±13.97)	0.04	1507.09(±380.59)	1460.2(±444.07)	0.06	0.28(±1.08)	0.58(±5.51)	0.60
CE(18:3)	Cholesterol Esters	0.83(±1.58)	1.13(±1.72)	0.001	86.18(±32.87)	78.57(±31.84)	0.01	0.01(±0.04)	0.02(±0.19)	0.58
CE(20:0)	Cholesterol Esters	2.7(±2.99)	2.91(±2.96)	0.41	1.39(±0.92)	1.26(±0.93)	0.31	0.14(±0.31)	0.17(±0.42)	0.25
CE(20:1)	Cholesterol Esters	0.76(±1.37)	0.94(±1.6)	0.37	0.87(±0.52)	0.83(±0.55)	0.17	0.01(±0.05)	0.02(±0.07)	0.18
CE(20:3)	Cholesterol Esters	0.61(±1.12)	0.87(±1.36)	0.01	33.33(±10.8)	33.18(±11.43)	0.80	0.01(±0.02)	0.01(±0.09)	0.82
CE(20:4)	Cholesterol Esters	1.4(±2.81)	1.71(±2.66)	0.01	291.26(±84.15)	288.43(±89.57)	0.84	0.05(±0.15)	0.09(±0.89)	0.11
CE(20:5)	Cholesterol Esters	0.94(±1.86)	1.01(±1.6)	0.20	89.15(±42.04)	77.16(±44.85)	0.001	0.01(±0.03)	0.02(±0.11)	0.93
CE(22:0)	Cholesterol Esters	0.44(±0.87)	0.46(±0.8)	0.99	0.29(±0.32)	0.23(±0.31)	0.06	0.03(±0.06)	0.03(±0.06)	0.39
CE(22:1)	Cholesterol Esters	0.28(±0.38)	0.29(±0.41)	0.86	0.22(±0.23)	0.22(±0.23)	0.89	0.04(±0.08)	0.04(±0.07)	0.21
CE(22:2)	Cholesterol Esters	0.21(±0.32)	0.23(±0.34)	0.64	0.08(±0.11)	0.08(±0.11)	0.94	0(±0.01)	0(±0.02)	0.65
CE(22:5)	Cholesterol Esters	0.55(±0.77)	0.66(±0.77)	0.21	3.08(±0.86)	3(±0.99)	0.29	0.02(±0.04)	0.01(±0.04)	0.40
CE(22:6)	Cholesterol Esters	1.73(±8.52)	1.25(±1.84)	0.45	43.47(±16.88)	40.67(±15.7)	0.07	0.01(±0.02)	0.01(±0.09)	0.42
p-Cresol-SO4	Cresols	2.1(±5.62)	2.23(±6.6)	0.93	21.76(±13.25)	22.83(±17.64)	0.97	13.4(±8.39)	13.36(±9.68)	0.54
DG(14:0_14:0)	Diacylglycerols	0.06(±0.14)	0.08(±0.3)	0.88	0.07(±0.03)	0.06(±0.03)	0.10	0(±0)	0(±0)	0.54
DG(14:0_18:1)	Diacylglycerols	0.94(±1.24)	1(±2.37)	0.09	0.55(±0.25)	0.53(±0.33)	0.14	0.01(±0.02)	0.01(±0.02)	0.56
DG(14:0_18:2)	Diacylglycerols	0.96(±0.95)	0.84(±1)	0.07	0.29(±0.11)	0.27(±0.12)	0.04	0.01(±0.02)	0.01(±0.02)	0.40
DG(14:0_20:0)	Diacylglycerols	0.3(±0.18)	0.35(±0.25)	0.18	0.23(±0.27)	0.22(±0.27)	0.37	0.08(±0.18)	0.08(±0.16)	0.99
DG(14:1_18:1)	Diacylglycerols	0.58(±0.41)	0.53(±0.39)	0.22	0.1(±0.11)	0.08(±0.12)	0.048	0(±0.01)	0(±0.01)	0.41
DG(14:1_20:2)	Diacylglycerols	0.29(±0.92)	0.34(±1.11)	0.84	0.01(±0.03)	0.01(±0.02)	0.33	0(±0.01)	0(±0.01)	0.14
DG(16:0_16:0)	Diacylglycerols	30.52(±7.49)	30.15(±8.65)	0.53	9.23(±1.95)	9.31(±2.08)	0.82	65.6(±178.69)	63.63(±169.96)	0.47
DG(16:0_16:1)	Diacylglycerols	2.31(±2.43)	4.55(±9.03)	0.01	0.66(±0.65)	0.72(±0.62)	0.37	0.03(±0.05)	0.03(±0.05)	0.81
DG(16:0_18:1)	Diacylglycerols	33.6(±51.5)	29.79(±70.08)	0.02	5.14(±2.77)	5.44(±3.75)	0.66	0.1(±0.15)	0.1(±0.15)	0.63
DG(16:0_18:2)	Diacylglycerols	37.03(±60.29)	22.46(±36.94)	0.0004	2.03(±1.02)	2.02(±1.2)	0.78	0.01(±0.03)	0.01(±0.03)	0.58
DG(16:0_20:0)	Diacylglycerols	1.62(±0.69)	1.63(±1.14)	0.53	0.6(±0.21)	0.55(±0.25)	0.003	0.92(±2.5)	0.89(±2.44)	0.31
DG(16:0_20:3)	Diacylglycerols	0.22(±0.21)	0.17(±0.2)	0.03	0.01(±0.02)	0.01(±0.03)	0.72	0(±0.01)	0(±0)	0.73
DG(16:0_20:4)	Diacylglycerols	0.49(±0.47)	0.51(±0.53)	0.91	0.12(±0.2)	0.12(±0.19)	0.64	0.06(±0.12)	0.07(±0.11)	0.12
DG(16:1_18:0)	Diacylglycerols	0.78(±0.92)	1.42(±2.82)	0.15	0.06(±0.09)	0.07(±0.09)	0.85	0.01(±0.02)	0.01(±0.02)	0.69
DG(16:1_18:1)	Diacylglycerols	4.67(±4.43)	5.98(±13.12)	0.31	3.37(±3.21)	3.43(±3.47)	0.40	0.23(±0.34)	0.24(±0.35)	0.29

Metabolite	Metabolite Class	Stool (no neo.) Mean (±SD)	Stool (ACN) Mean (±SD)	P Value	Blood (no neo.) Mean (±SD)	Blood (ACN) Mean (±SD)	P Value	Urine (no neo.) Mean (±SD)	Urine (ACN) Mean (±SD)	P Value
DG(16:1_18:2)	Diacylglycerols	1.91(±2.35)	1.76(±4.18)	0.02	0.55(±0.3)	0.53(±0.35)	0.44	0.01(±0.02)	0(±0.01)	0.82
DG(16:1_20:0)	Diacylglycerols	0.51(±0.52)	0.59(±0.67)	0.39	0.05(±0.23)	0.05(±0.1)	0.14	0.01(±0.03)	0.01(±0.02)	1.00
DG(17:0_17:1)	Diacylglycerols	0.4(±0.46)	0.42(±0.49)	1.00	0.02(±0.06)	0.02(±0.06)	0.95	0.03(±0.12)	0.03(±0.09)	0.12
DG(17:0_18:1)	Diacylglycerols	2.08(±1.56)	1.8(±1.51)	0.06	0.61(±0.35)	0.6(±0.38)	0.25	0.02(±0.03)	0.02(±0.04)	0.58
DG(18:0_20:0)	Diacylglycerols	0.66(±0.38)	0.64(±0.44)	0.29	0.34(±0.4)	0.37(±0.45)	0.97	0.51(±1.36)	0.51(±1.32)	0.63
DG(18:0_20:4)	Diacylglycerols	0.42(±1.14)	0.44(±0.51)	0.03	0.02(±0.04)	0.02(±0.03)	0.17	0(±0.01)	0(±0.01)	0.37
DG(18:1_18:1)	Diacylglycerols	81.65(±166.44)	59.35(±161.58)	0.004	4.99(±2.51)	5.28(±3.32)	0.56	0.01(±0.02)	0.01(±0.01)	0.58
DG(18:1_18:2)	Diacylglycerols	156.25(±306.08)	83.1(±185.96)	0.0001	7.72(±3.73)	7.6(±4.06)	0.73	0.01(±0.02)	0.01(±0.04)	0.21
DG(18:1_18:3)	Diacylglycerols	10.93(±26.94)	7.91(±19.6)	0.004	0.73(±0.48)	0.65(±0.47)	0.08	0.01(±0.03)	0.01(±0.03)	0.86
DG(18:1_18:4)	Diacylglycerols	0.3(±0.34)	0.29(±0.37)	0.56	0.01(±0.03)	0.01(±0.03)	0.88	0(±0.01)	0(±0.01)	0.87
DG(18:1_20:0)	Diacylglycerols	2.32(±3.63)	2.15(±3.68)	0.10	0.35(±0.24)	0.32(±0.27)	0.09	0.01(±0.02)	0.01(±0.02)	0.44
DG(18:1_20:1)	Diacylglycerols	0.59(±1.19)	0.53(±1.09)	0.01	0.12(±0.08)	0.12(±0.06)	0.29	0(±0)	0(±0)	0.57
DG(18:1_20:2)	Diacylglycerols	0.14(±0.15)	0.15(±0.17)	0.55	0.03(±0.04)	0.03(±0.04)	0.25	0(±0)	0(±0)	0.45
DG(18:1_20:3)	Diacylglycerols	0.23(±0.21)	0.23(±0.23)	0.72	0.07(±0.07)	0.08(±0.07)	0.06	0(±0.01)	0(±0)	0.93
DG(18:1_20:4)	Diacylglycerols	1.81(±1.65)	2.05(±1.66)	0.15	0.25(±0.21)	0.28(±0.2)	0.22	0.02(±0.03)	0.01(±0.03)	0.12
DG(18:1_22:5)	Diacylglycerols	0.07(±0.08)	0.08(±0.08)	0.23	0.01(±0.01)	0.01(±0.01)	0.63	0(±0)	0(±0)	0.93
DG(18:1_22:6)	Diacylglycerols	2.04(±1.82)	2.13(±2.58)	0.66	0.65(±0.53)	0.66(±0.59)	0.55	0.04(±0.06)	0.04(±0.06)	0.80
DG(18:2_18:2)	Diacylglycerols	235.66(±561.51)	100.19(±212.86)	0.001	3.21(±1.91)	2.93(±1.7)	0.14	0.01(±0.04)	0.01(±0.04)	0.47
DG(18:2_18:3)	Diacylglycerols	8.91(±25.22)	7.27(±23.16)	0.18	0.23(±0.26)	0.18(±0.24)	0.04	0.01(±0.02)	0(±0.02)	0.85
DG(18:2_18:4)	Diacylglycerols	0.14(±0.28)	0.16(±0.26)	0.96	0(±0.01)	0(±0.01)	0.40	0(±0)	0(±0)	0.56
DG(18:2_20:0)	Diacylglycerols	0.65(±0.86)	0.43(±0.45)	0.002	0.03(±0.06)	0.03(±0.05)	0.82	0(±0.01)	0(±0.01)	0.69
DG(18:2_20:4)	Diacylglycerols	0.78(±0.68)	0.83(±0.67)	0.51	0.1(±0.12)	0.1(±0.11)	0.42	0(±0.01)	0(±0.01)	0.58
DG(18:3_18:3)	Diacylglycerols	3.56(±19.38)	3.06(±13.16)	0.91	0.01(±0.02)	0.01(±0.03)	0.58	0(±0.01)	0(±0.01)	0.51
DG(18:3_20:2)	Diacylglycerols	0.14(±0.24)	0.14(±0.23)	0.95	0.01(±0.02)	0.01(±0.02)	0.78	0(±0.01)	0(±0.01)	0.85
DG(21:0_22:6)	Diacylglycerols	0.13(±0.22)	0.12(±0.22)	0.57	0.01(±0.03)	0.01(±0.03)	0.36	0(±0.01)	0(±0)	0.47
DG(22:1_22:2)	Diacylglycerols	0.04(±0.06)	0.03(±0.06)	0.14	0(±0)	0(±0)	1.00	0(±0)	0(±0)	0.61
DG-O(14:0_18:2)	Diacylglycerols	0.56(±0.48)	0.6(±0.55)	0.85	0.17(±0.11)	0.17(±0.12)	0.76	0.01(±0.02)	0.01(±0.02)	0.58
DG-O(16:0_18:1)	Diacylglycerols	0.46(±0.36)	0.44(±0.41)	0.21	0.03(±0.07)	0.03(±0.06)	0.84	0.02(±0.04)	0.02(±0.05)	0.91
DG-O(16:0_20:4)	Diacylglycerols	0.02(±0.03)	0.02(±0.03)	0.55	0(±0)	0(±0)	0.87	0(±0)	0(±0)	0.90
Cer(d18:0/18:0)	Dihydroceramides	0.1(±0.08)	0.11(±0.11)	0.67	0.01(±0.01)	0.01(±0.01)	0.69	0.02(±0.04)	0.01(±0.03)	0.19
Cer(d18:0/18:0(OH))	Dihydroceramides	7.46(±11.21)	7.64(±10.97)	0.65	0.23(±0.45)	0.19(±0.35)	0.70	0.07(±0.12)	0.07(±0.1)	0.39
Cer(d18:0/20:0)	Dihydroceramides	0.28(±0.25)	0.26(±0.19)	0.84	0.03(±0.04)	0.03(±0.04)	0.70	0.01(±0.01)	0.01(±0.01)	0.25

Metabolite	Metabolite Class	Stool (no neo.) Mean (±SD)	Stool (ACN) Mean (±SD)	P Value	Blood (no neo.) Mean (±SD)	Blood (ACN) Mean (±SD)	P Value	Urine (no neo.) Mean (±SD)	Urine (ACN) Mean (±SD)	P Value
Cer(d18:0/22:0)	Dihydroceramides	0.38(±0.3)	0.35(±0.26)	0.53	0.1(±0.08)	0.1(±0.08)	0.21	0(±0.01)	0(±0.01)	0.20
Cer(d18:0/24:0)	Dihydroceramides	0.62(±0.64)	0.61(±0.46)	0.50	0.17(±0.13)	0.17(±0.12)	0.51	0(±0)	0(±0)	0.02
Cer(d18:0/24:1)	Dihydroceramides	0.54(±0.47)	0.55(±0.32)	0.37	0.17(±0.09)	0.17(±0.1)	0.59	0(±0.01)	0(±0)	0.99
Cer(d18:0/26:1)	Dihydroceramides	0.06(±0.07)	0.07(±0.07)	0.61	0(±0)	0(±0.01)	0.11	0(±0)	0(±0)	0.33
Cer(d18:0/26:1(OH))	Dihydroceramides	0.55(±0.62)	0.57(±0.64)	0.93	0.15(±0.29)	0.12(±0.22)	0.80	0.03(±0.06)	0.04(±0.06)	0.51
AA	Fatty Acids	20.56(±20.94)	27.96(±35.36)	0.09	3.05(±1.71)	3.42(±2.14)	0.07	0.01(±0.02)	0.01(±0.05)	0.62
DHA	Fatty Acids	11.43(±17.06)	20.77(±56.1)	0.77	4.33(±3.15)	4.37(±2.89)	0.61	0.01(±0.03)	0.01(±0.03)	0.56
EPA	Fatty Acids	2.98(±3.42)	5.35(±21.48)	0.79	0.55(±0.38)	0.54(±0.37)	0.73	0.01(±0.01)	0.01(±0.01)	0.37
FA(12:0)	Fatty Acids	298.12(±758.93)	257.14(±491.98)	0.65	9.57(±4.45)	9.38(±4)	0.96	1.81(±16.16)	0.37(±0.52)	0.20
FA(14:0)	Fatty Acids	1524.68(±1093.04)	1585.8(±1243.66)	0.69	28.49(±11)	30(±13.75)	0.49	1.49(±2.78)	1.12(±2.45)	0.04
FA(16:0)	Fatty Acids	4089.18(±1987.76)	4310.67(±2259.02)	0.36	323.73(±62.77)	337.67(±74.81)	0.14	72.16(±64.66)	67.09(±64.18)	0.08
FA(18:0)	Fatty Acids	3671.69(±2485.8)	4083.25(±3065.6)	0.34	247.69(±52.7)	252.65(±56.05)	0.32	79.23(±73.68)	72.95(±72.07)	0.04
FA(18:1)	Fatty Acids	5610.03(±4556.37)	5469.07(±5065.28)	0.34	157.02(±96.43)	188.19(±113.26)	0.01	2(±2.15)	1.89(±2.48)	0.12
FA(18:2)	Fatty Acids	2021.26(±1338.32)	1796.18(±1396.74)	0.03	106.86(±64.54)	118.08(±71.23)	0.12	1.13(±1.08)	1.18(±2.17)	0.03
FA(20:1)	Fatty Acids	243.11(±223.12)	319.66(±394.39)	0.17	3.19(±2.2)	4.05(±2.97)	0.003	0.17(±0.22)	0.16(±0.21)	0.14
FA(20:2)	Fatty Acids	116.14(±155.35)	124.45(±139.69)	0.58	1.79(±1.26)	2.19(±1.54)	0.003	0.04(±0.04)	0.04(±0.04)	0.05
FA(20:3)	Fatty Acids	39.24(±85.05)	51.75(±75.74)	0.04	0.87(±0.6)	1(±0.64)	0.01	0.03(±0.05)	0.03(±0.05)	0.15
lysoPC a C14:0	Glycerophospholipids	9.57(±0.75)	9.45(±0.6)	0.06	4.91(±0.63)	4.74(±0.65)	0.0024	1.15(±1.05)	1.03(±1.01)	0.04
lysoPC a C16:0	Glycerophospholipids	13.23(±18.08)	15.26(±20.14)	0.35	91.91(±19.71)	84.05(±21.15)	<.0001	0.04(±0.17)	0.07(±0.56)	0.09
lysoPC a C16:1	Glycerophospholipids	0.44(±0.36)	0.52(±0.53)	0.59	3(±1.06)	2.75(±1.02)	0.00	0.01(±0.01)	0.01(±0.01)	0.17
lysoPC a C17:0	Glycerophospholipids	0.36(±0.28)	0.47(±0.51)	0.16	1.75(±0.54)	1.49(±0.51)	<.0001	0(±0.01)	0(±0.01)	0.54
lysoPC a C18:0	Glycerophospholipids	3.93(±3.89)	5.23(±6.96)	0.15	26.73(±6.61)	24.22(±7.06)	0.0001	0.03(±0.06)	0.04(±0.2)	0.06
lysoPC a C18:1	Glycerophospholipids	5.25(±9.38)	5.52(±11.33)	0.73	21.95(±6.47)	19.55(±6.44)	0.0002	0.01(±0.03)	0.02(±0.09)	0.10
lysoPC a C18:2	Glycerophospholipids	6.02(±24.31)	5.99(±22.51)	0.45	30.68(±11.53)	25.43(±11.2)	<.0001	0.01(±0.04)	0.02(±0.14)	0.22
lysoPC a C20:3	Glycerophospholipids	0.18(±0.08)	0.2(±0.1)	0.02	2.27(±0.75)	2.08(±0.71)	0.003	0.01(±0.01)	0.01(±0.02)	0.54
lysoPC a C20:4	Glycerophospholipids	0.1(±0.1)	0.12(±0.15)	0.20	6.46(±1.95)	5.98(±1.94)	0.01	0(±0.01)	0.01(±0.04)	0.27
lysoPC a C24:0	Glycerophospholipids	0.12(±0.21)	0.16(±0.33)	0.21	0.18(±0.05)	0.18(±0.05)	0.46	0.01(±0.01)	0.01(±0.01)	0.23
lysoPC a C26:0	Glycerophospholipids	0.1(±0.09)	0.1(±0.09)	0.87	0.33(±0.16)	0.34(±0.21)	0.92	0(±0.01)	0(±0.01)	0.44
lysoPC a C26:1	Glycerophospholipids	0.04(±0.05)	0.04(±0.05)	0.15	0.16(±0.06)	0.17(±0.13)	0.59	0(±0)	0(±0)	0.44
lysoPC a C28:0	Glycerophospholipids	0.21(±0.14)	0.21(±0.12)	0.19	0.34(±0.13)	0.33(±0.17)	0.25	0.06(±0.05)	0.05(±0.05)	0.02
lysoPC a C28:1	Glycerophospholipids	0.09(±0.12)	0.08(±0.11)	0.80	0.41(±0.13)	0.39(±0.17)	0.03	0(±0)	0(±0)	0.81
PC aa C24:0	Glycerophospholipids	0.06(±0.07)	0.05(±0.06)	0.46	0.13(±0.08)	0.14(±0.09)	0.69	0.01(±0.02)	0.01(±0.08)	0.19

Metabolite	Metabolite Class	Stool (no neo.) Mean (±SD)	Stool (ACN) Mean (±SD)	P Value	Blood (no neo.) Mean (±SD)	Blood (ACN) Mean (±SD)	P Value	Urine (no neo.) Mean (±SD)	Urine (ACN) Mean (±SD)	P Value
PC aa C26:0	Glycerophospholipids	1.16(±0.08)	1.15(±0.07)	0.21	0.82(±0.32)	0.85(±0.7)	0.69	0.08(±0.07)	0.07(±0.06)	0.06
PC aa C28:1	Glycerophospholipids	0.12(±0.12)	0.13(±0.15)	0.85	3.91(±1.02)	3.68(±1.07)	0.02	0.01(±0)	0.01(±0.01)	0.51
PC aa C30:0	Glycerophospholipids	1.1(±1.1)	1.18(±1.28)	0.91	4.75(±1.62)	4.49(±1.51)	0.11	0.01(±0.01)	0.01(±0.01)	0.04
PC aa C30:2	Glycerophospholipids	0.01(±0.02)	0.01(±0.01)	0.17	0.04(±0.06)	0.04(±0.07)	0.81	0(±0)	0(±0)	0.41
PC aa C32:0	Glycerophospholipids	1.63(±2.66)	1.35(±1.32)	0.94	16.63(±3.44)	16.77(±3.84)	0.93	0.01(±0.02)	0.01(±0.04)	0.10
PC aa C32:1	Glycerophospholipids	0.49(±0.7)	0.48(±0.64)	0.64	21.75(±10.62)	22.59(±11.81)	0.75	0(±0.01)	0.01(±0.03)	0.38
PC aa C32:2	Glycerophospholipids	0.36(±2.25)	0.23(±0.5)	0.29	5.24(±2.19)	4.62(±1.95)	0.01	0(±0)	0(±0.01)	0.59
PC aa C32:3	Glycerophospholipids	0.06(±0.15)	0.05(±0.07)	0.74	0.63(±0.17)	0.57(±0.16)	0.00	0(±0)	0(±0)	0.84
PC aa C34:1	Glycerophospholipids	4.88(±9.56)	4.17(±5.32)	0.41	251.88(±60.24)	258.64(±65.15)	0.37	0.05(±0.14)	0.07(±0.56)	0.06
PC aa C34:2	Glycerophospholipids	5.37(±36.07)	2.35(±3.17)	0.13	405.41(±82.38)	395.86(±81.72)	0.27	0.07(±0.32)	0.16(±1.4)	0.15
PC aa C34:3	Glycerophospholipids	0.62(±2.99)	0.39(±1.03)	0.52	19.07(±6.19)	17.31(±5.4)	0.01	0(±0.01)	0(±0.03)	0.53
PC aa C34:4	Glycerophospholipids	0.05(±0.06)	0.05(±0.06)	0.32	2.25(±0.79)	2.01(±0.75)	0.001	0(±0)	0(±0.01)	0.12
PC aa C36:0	Glycerophospholipids	0.35(±0.09)	0.35(±0.11)	0.94	1.56(±0.73)	1.42(±0.68)	0.03	0.02(±0.02)	0.02(±0.02)	0.03
PC aa C36:1	Glycerophospholipids	1.15(±1.54)	1.31(±2.49)	0.48	56.63(±15.49)	56.01(±16.01)	0.73	0.02(±0.04)	0.02(±0.14)	0.04
PC aa C36:2	Glycerophospholipids	3.26(±10.53)	2.21(±3.43)	0.53	236.65(±53.72)	231.95(±53.68)	0.40	0.05(±0.19)	0.09(±0.75)	0.13
PC aa C36:3	Glycerophospholipids	3.78(±18.58)	1.54(±3.26)	0.14	139.8(±31.02)	138.08(±33.42)	0.24	0.02(±0.09)	0.04(±0.36)	0.65
PC aa C36:4	Glycerophospholipids	5.09(±40.33)	1.5(±3.35)	0.38	204.98(±44.1)	207.58(±48.33)	0.77	0.03(±0.14)	0.06(±0.58)	0.59
PC aa C36:5	Glycerophospholipids	0.85(±5.81)	0.37(±1.05)	0.72	31.3(±12.72)	27.52(±11.36)	0.003	0.05(±0.04)	0.04(±0.05)	0.04
PC aa C36:6	Glycerophospholipids	0.16(±0.62)	0.15(±0.58)	0.45	1.09(±0.41)	0.92(±0.36)	<.0001	0(±0)	0(±0)	0.32
PC aa C38:0	Glycerophospholipids	0.15(±0.09)	0.16(±0.13)	0.90	2.5(±0.76)	2.45(±0.82)	0.30	0(±0)	0(±0.01)	0.86
PC aa C38:1	Glycerophospholipids	0.06(±0.08)	0.08(±0.14)	0.12	0.87(±0.43)	0.83(±0.41)	0.34	0(±0)	0(±0)	0.06
PC aa C38:3	Glycerophospholipids	0.19(±0.43)	0.2(±0.25)	0.32	51.48(±12.48)	52.15(±13.47)	0.76	0.01(±0.03)	0.02(±0.14)	0.35
PC aa C38:4	Glycerophospholipids	0.21(±0.32)	0.24(±0.5)	0.65	110.45(±25.7)	113.98(±26.56)	0.16	0.02(±0.08)	0.04(±0.34)	0.27
PC aa C38:5	Glycerophospholipids	0.13(±0.14)	0.18(±0.35)	0.24	56.99(±12.37)	54.55(±12.71)	0.03	0.01(±0.03)	0.02(±0.13)	0.85
PC aa C38:6	Glycerophospholipids	0.19(±0.22)	0.29(±0.95)	0.99	79.58(±24.23)	76.57(±21.9)	0.31	0.01(±0.03)	0.02(±0.14)	0.25
PC aa C40:1	Glycerophospholipids	0.42(±0.04)	0.43(±0.06)	0.96	0.33(±0.07)	0.33(±0.06)	0.16	0.03(±0.03)	0.03(±0.03)	0.08
PC aa C40:2	Glycerophospholipids	0.03(±0.04)	0.03(±0.04)	0.58	0.26(±0.15)	0.24(±0.07)	0.06	0(±0)	0(±0)	0.39
PC aa C40:3	Glycerophospholipids	0.02(±0.03)	0.02(±0.03)	0.68	0.47(±0.2)	0.44(±0.11)	0.07	0(±0)	0(±0)	0.76
PC aa C40:4	Glycerophospholipids	0.03(±0.03)	0.04(±0.04)	0.003	3.05(±0.76)	3.17(±0.83)	0.23	0(±0)	0(±0.01)	0.35
PC aa C40:5	Glycerophospholipids	0.02(±0.04)	0.02(±0.06)	0.54	8.53(±2.14)	8.57(±2.35)	0.87	0(±0.01)	0(±0.02)	0.10
PC aa C40:6	Glycerophospholipids	0.36(±0.08)	0.39(±0.29)	0.46	23.71(±7.46)	23.17(±6.74)	0.52	0.02(±0.02)	0.02(±0.05)	0.03
PC aa C42:0	Glycerophospholipids	0.05(±0.03)	0.06(±0.03)	0.28	0.47(±0.13)	0.46(±0.14)	0.15	0(±0)	0(±0.01)	0.02

Metabolite	Metabolite Class	Stool (no neo.) Mean (±SD)	Stool (ACN) Mean (±SD)	P Value	Blood (no neo.) Mean (±SD)	Blood (ACN) Mean (±SD)	P Value	Urine (no neo.) Mean (±SD)	Urine (ACN) Mean (±SD)	P Value
PC aa C42:1	Glycerophospholipids	0.04(±0.03)	0.04(±0.03)	0.37	0.24(±0.06)	0.23(±0.06)	0.21	0(±0)	0(±0)	0.46
PC aa C42:2	Glycerophospholipids	0.1(±0.04)	0.1(±0.04)	0.76	0.19(±0.05)	0.18(±0.05)	0.002	0.01(±0.01)	0.01(±0.01)	0.10
PC aa C42:4	Glycerophospholipids	0.01(±0.02)	0.01(±0.02)	0.60	0.16(±0.03)	0.15(±0.03)	0.21	0(±0)	0(±0)	0.77
PC aa C42:5	Glycerophospholipids	0.03(±0.03)	0.03(±0.03)	0.97	0.3(±0.09)	0.3(±0.08)	0.45	0(±0)	0(±0)	0.99
PC aa C42:6	Glycerophospholipids	0.15(±0.03)	0.15(±0.04)	0.13	0.41(±0.1)	0.38(±0.1)	0.004	0.01(±0.01)	0.01(±0.01)	0.09
PC ae C30:0	Glycerophospholipids	0.31(±0.38)	0.35(±0.46)	0.88	0.4(±0.14)	0.37(±0.12)	0.04	0.01(±0.01)	0.01(±0.01)	0.23
PC ae C30:1	Glycerophospholipids	0.05(±0.12)	0.06(±0.1)	0.22	0.16(±0.11)	0.15(±0.18)	0.01	0(±0)	0(±0)	0.14
PC ae C30:2	Glycerophospholipids	0.02(±0.03)	0.02(±0.03)	0.62	0.11(±0.03)	0.1(±0.03)	0.01	0(±0)	0(±0)	0.81
PC ae C32:1	Glycerophospholipids	0.2(±0.26)	0.22(±0.32)	0.95	3.2(±0.78)	3.21(±0.8)	0.86	0(±0.01)	0(±0.01)	0.73
PC ae C32:2	Glycerophospholipids	0.08(±0.16)	0.08(±0.15)	0.92	0.85(±0.22)	0.83(±0.22)	0.36	0(±0)	0(±0)	0.39
PC ae C34:0	Glycerophospholipids	0.53(±1.21)	0.66(±1.36)	0.76	1.72(±0.55)	1.66(±0.52)	0.21	0(±0)	0(±0)	0.65
PC ae C34:1	Glycerophospholipids	0.79(±1.07)	0.98(±1.59)	0.81	12.49(±3.19)	12.22(±3.12)	0.63	0.01(±0.03)	0(±0.02)	0.07
PC ae C34:2	Glycerophospholipids	0.63(±0.98)	0.74(±1.49)	0.88	13.67(±4.01)	13.17(±4.03)	0.18	0(±0.01)	0.01(±0.03)	0.24
PC ae C34:3	Glycerophospholipids	0.24(±0.31)	0.26(±0.47)	0.61	8.65(±2.7)	8.15(±2.68)	0.06	0(±0.01)	0(±0.03)	0.02
PC ae C36:0	Glycerophospholipids	0.28(±0.11)	0.29(±0.13)	0.58	0.77(±0.2)	0.78(±0.21)	0.44	0.01(±0.01)	0.01(±0.01)	0.04
PC ae C36:1	Glycerophospholipids	0.32(±0.35)	0.33(±0.38)	0.32	9.37(±2.48)	8.95(±2.38)	0.08	0.01(±0.01)	0.01(±0.03)	0.06
PC ae C36:2	Glycerophospholipids	0.35(±0.43)	0.36(±0.48)	0.26	15.26(±4.64)	14.28(±4.27)	0.04	0.01(±0.02)	0.01(±0.04)	0.02
PC ae C36:3	Glycerophospholipids	0.23(±0.37)	0.28(±0.57)	0.77	8.26(±2.28)	7.92(±2.22)	0.11	0(±0.01)	0(±0.02)	0.25
PC ae C36:4	Glycerophospholipids	0.22(±0.39)	0.29(±0.58)	0.78	18.7(±4.83)	19.12(±5.42)	0.61	0.01(±0.02)	0.01(±0.05)	0.56
PC ae C36:5	Glycerophospholipids	0.14(±0.17)	0.17(±0.27)	0.95	12.27(±3.17)	12.19(±3.53)	0.59	0(±0.01)	0(±0.04)	0.40
PC ae C38:0	Glycerophospholipids	0.22(±0.06)	0.24(±0.16)	0.53	2.12(±0.62)	1.88(±0.54)	0.0001	0.01(±0.01)	0.01(±0.01)	0.01
PC ae C38:1	Glycerophospholipids	0.06(±0.1)	0.07(±0.09)	0.85	0.21(±0.25)	0.24(±0.59)	0.76	0(±0)	0(±0.01)	0.91
PC ae C38:2	Glycerophospholipids	0.1(±0.18)	0.08(±0.09)	0.05	1.82(±0.56)	1.72(±0.51)	0.05	0(±0.01)	0(±0.01)	0.24
PC ae C38:3	Glycerophospholipids	0.13(±0.34)	0.09(±0.1)	0.24	4.26(±1.06)	4.11(±1.07)	0.11	0(±0.01)	0(±0.02)	0.046
PC ae C38:4	Glycerophospholipids	0.18(±0.18)	0.22(±0.28)	0.24	13.69(±2.88)	14.11(±3.18)	0.33	0.01(±0.01)	0.01(±0.04)	0.03
PC ae C38:5	Glycerophospholipids	0.17(±0.23)	0.22(±0.39)	0.69	18.02(±3.74)	18.39(±4.46)	0.90	0.02(±0.03)	0.02(±0.06)	0.04
PC ae C38:6	Glycerophospholipids	0.1(±0.11)	0.13(±0.38)	0.35	7.23(±1.83)	6.94(±2.04)	0.04	0.01(±0.01)	0.01(±0.02)	0.047
PC ae C40:1	Glycerophospholipids	0.05(±0.05)	0.06(±0.06)	0.78	1.17(±0.28)	1.06(±0.29)	0.0002	0(±0)	0(±0)	0.98
PC ae C40:2	Glycerophospholipids	0.05(±0.06)	0.05(±0.08)	0.94	1.89(±0.49)	1.81(±0.48)	0.10	0(±0)	0(±0.01)	0.67
PC ae C40:3	Glycerophospholipids	0.03(±0.04)	0.04(±0.05)	0.40	1.04(±0.27)	1.04(±0.29)	0.59	0(±0)	0(±0.01)	0.12
PC ae C40:4	Glycerophospholipids	0.11(±0.05)	0.12(±0.06)	0.23	2.15(±0.45)	2.21(±0.48)	0.30	0.01(±0.01)	0.01(±0.01)	0.08
PC ae C40:5	Glycerophospholipids	0.04(±0.05)	0.05(±0.09)	0.81	3.16(±0.62)	3.15(±0.7)	0.57	0(±0)	0(±0.02)	0.32

Metabolite	Metabolite Class	Stool (no neo.) Mean (±SD)	Stool (ACN) Mean (±SD)	P Value	Blood (no neo.) Mean (±SD)	Blood (ACN) Mean (±SD)	P Value	Urine (no neo.) Mean (±SD)	Urine (ACN) Mean (±SD)	P Value
PC ae C40:6	Glycerophospholipids	0.05(±0.05)	0.08(±0.19)	0.42	4.12(±1.11)	3.94(±1.12)	0.05	0(±0)	0(±0.01)	0.66
PC ae C42:0	Glycerophospholipids	1.08(±0.1)	1.11(±0.1)	0.04	0.62(±0.09)	0.62(±0.09)	0.38	0.07(±0.06)	0.06(±0.06)	0.09
PC ae C42:1	Glycerophospholipids	0.11(±0.04)	0.11(±0.05)	0.77	0.33(±0.07)	0.33(±0.09)	0.64	0.01(±0.01)	0.01(±0.01)	0.66
PC ae C42:2	Glycerophospholipids	0.03(±0.05)	0.04(±0.06)	0.72	0.51(±0.11)	0.5(±0.13)	0.17	0(±0)	0(±0)	0.97
PC ae C42:3	Glycerophospholipids	0.03(±0.05)	0.03(±0.05)	0.89	0.71(±0.19)	0.68(±0.18)	0.07	0(±0)	0(±0)	0.27
PC ae C42:4	Glycerophospholipids	0(±0.01)	0.01(±0.04)	0.26	0.74(±0.18)	0.77(±0.22)	0.31	0(±0)	0(±0)	0.31
PC ae C42:5	Glycerophospholipids	0.71(±0.04)	0.72(±0.05)	0.08	1.74(±0.34)	1.78(±0.42)	0.64	0.05(±0.05)	0.05(±0.05)	0.06
PC ae C44:3	Glycerophospholipids	0.07(±0.04)	0.07(±0.03)	0.44	0.14(±0.03)	0.13(±0.04)	0.51	0(±0)	0(±0)	0.13
PC ae C44:4	Glycerophospholipids	0.12(±0.03)	0.12(±0.03)	0.94	0.34(±0.08)	0.35(±0.09)	0.33	0.01(±0.01)	0.01(±0.01)	0.02
PC ae C44:5	Glycerophospholipids	0.07(±0.03)	0.08(±0.05)	0.79	1.38(±0.34)	1.42(±0.44)	0.37	0.01(±0.01)	0.01(±0.01)	0.43
PC ae C44:6	Glycerophospholipids	0.08(±0.02)	0.07(±0.03)	0.13	0.92(±0.23)	0.92(±0.26)	0.57	0.01(±0.01)	0(±0.01)	0.06
Hex2Cer(d18:1/14:0)	Glycosylceramides	0.11(±0.14)	0.13(±0.17)	0.48	0.18(±0.1)	0.16(±0.09)	0.05	0(±0)	0(±0)	0.18
Hex2Cer(d18:1/16:0)	Glycosylceramides	1.77(±2.3)	2.25(±5.04)	0.58	2.42(±0.6)	2.5(±0.71)	0.41	0.01(±0.08)	0(±0.01)	0.82
Hex2Cer(d18:1/18:0)	Glycosylceramides	1.08(±1.24)	1.09(±1.56)	0.32	0.17(±0.05)	0.17(±0.05)	0.95	0(±0)	0(±0)	0.54
Hex2Cer(d18:1/20:0)	Glycosylceramides	0.19(±0.17)	0.2(±0.24)	0.58	0.06(±0.04)	0.06(±0.04)	0.98	0(±0)	0(±0)	0.01
Hex2Cer(d18:1/22:0)	Glycosylceramides	0.93(±1.1)	0.98(±1.35)	0.32	0.16(±0.06)	0.17(±0.06)	0.18	0(±0.01)	0(±0)	0.35
Hex2Cer(d18:1/24:0)	Glycosylceramides	0.81(±1.08)	0.82(±1.24)	0.15	0.16(±0.04)	0.16(±0.06)	0.61	0(±0.01)	0(±0)	0.17
Hex2Cer(d18:1/24:1)	Glycosylceramides	0.67(±0.97)	0.86(±1.98)	0.63	0.38(±0.1)	0.39(±0.12)	0.57	0(±0.03)	0(±0)	0.51
Hex2Cer(d18:1/26:0)	Glycosylceramides	0.05(±0.08)	0.05(±0.08)	0.53	0(±0.01)	0(±0.01)	0.69	0(±0)	0(±0)	0.86
Hex2Cer(d18:1/26:1)	Glycosylceramides	0.02(±0.05)	0.03(±0.07)	0.20	0(±0.01)	0.01(±0.02)	0.49	0(±0)	0(±0)	0.84
Hex3Cer(d18:1/16:0)	Glycosylceramides	0.21(±0.27)	0.23(±0.33)	0.73	1.03(±0.23)	1.03(±0.27)	0.77	0(±0)	0(±0)	0.41
Hex3Cer(d18:1/18:0)	Glycosylceramides	0.11(±0.16)	0.1(±0.16)	0.29	0.1(±0.09)	0.1(±0.08)	0.63	0(±0)	0(±0)	0.16
Hex3Cer(d18:1_20:0)	Glycosylceramides	0.08(±0.11)	0.1(±0.12)	0.30	0.04(±0.05)	0.04(±0.05)	0.51	0(±0)	0(±0)	0.25
Hex3Cer(d18:1_22:0)	Glycosylceramides	0.09(±0.16)	0.08(±0.17)	0.26	0.22(±0.18)	0.21(±0.19)	0.40	0(±0)	0(±0)	0.54
Hex3Cer(d18:1/24:1)	Glycosylceramides	0.11(±0.14)	0.12(±0.16)	0.51	0.38(±0.13)	0.38(±0.12)	0.94	0(±0)	0(±0)	0.46
Hex3Cer(d18:1/26:1)	Glycosylceramides	0.1(±0.19)	0.12(±0.22)	0.71	0.03(±0.11)	0.04(±0.1)	0.28	0(±0)	0(±0)	0.85
HexCer(d16:1/22:0)	Glycosylceramides	0.11(±0.16)	0.12(±0.18)	0.89	0.16(±0.11)	0.15(±0.12)	0.37	0(±0)	0(±0)	0.65
HexCer(d16:1/24:0)	Glycosylceramides	0.1(±0.14)	0.11(±0.16)	0.76	0.07(±0.06)	0.07(±0.06)	0.63	0(±0)	0(±0)	0.26
HexCer(d18:1/14:0)	Glycosylceramides	0.25(±0.22)	0.23(±0.21)	0.64	0.01(±0.03)	0.01(±0.03)	0.27	0(±0)	0(±0)	0.60
HexCer(d18:1/16:0)	Glycosylceramides	2.21(±1.64)	2.44(±2.42)	0.66	1.03(±0.26)	1.08(±0.32)	0.26	0(±0)	0(±0)	0.54
HexCer(d18:1/18:0)	Glycosylceramides	0.62(±0.74)	0.64(±0.69)	0.55	0.14(±0.1)	0.15(±0.1)	0.14	0(±0)	0(±0)	0.43
HexCer(d18:1/18:1)	Glycosylceramides	0.21(±0.16)	0.18(±0.14)	0.08	0.04(±0.04)	0.04(±0.04)	0.58	0(±0)	0(±0)	0.59

Metabolite	Metabolite Class	Stool (no neo.) Mean (±SD)	Stool (ACN) Mean (±SD)	P Value	Blood (no neo.) Mean (±SD)	Blood (ACN) Mean (±SD)	P Value	Urine (no neo.) Mean (±SD)	Urine (ACN) Mean (±SD)	P Value
HexCer(d18:1/20:0)	Glycosylceramides	0.38(±0.34)	0.38(±0.37)	0.93	0.26(±0.1)	0.26(±0.12)	0.79	0(±0.01)	0(±0)	0.83
HexCer(d18:1/22:0)	Glycosylceramides	2.63(±2.52)	2.8(±2.86)	0.82	2.91(±0.83)	2.94(±0.99)	0.69	0(±0.01)	0(±0.02)	0.59
HexCer(d18:1/23:0)	Glycosylceramides	1.9(±1.72)	1.99(±1.97)	0.99	1.63(±0.48)	1.57(±0.53)	0.11	0(±0)	0(±0)	0.86
HexCer(d18:1/24:0)	Glycosylceramides	0.99(±0.96)	1.1(±1.2)	0.79	1.66(±0.47)	1.7(±0.56)	0.94	0(±0)	0(±0.01)	0.03
HexCer(d18:1/24:1)	Glycosylceramides	5.83(±4.35)	6.31(±5.58)	0.97	3.35(±0.9)	3.42(±1.16)	0.98	0.01(±0.02)	0.01(±0.03)	0.96
HexCer(d18:1/26:0)	Glycosylceramides	0.35(±0.25)	0.37(±0.29)	0.87	0.06(±0.06)	0.06(±0.06)	0.63	0(±0.01)	0(±0)	0.39
HexCer(d18:1/26:1)	Glycosylceramides	0.35(±0.39)	0.38(±0.44)	0.92	0.03(±0.05)	0.04(±0.05)	0.55	0(±0.01)	0(±0)	0.86
HexCer(d18:2/16:0)	Glycosylceramides	0.32(±0.29)	0.37(±0.36)	0.33	0.02(±0.03)	0.02(±0.03)	0.75	0(±0)	0(±0)	0.82
HexCer(d18:2/18:0)	Glycosylceramides	0.07(±0.11)	0.09(±0.13)	0.14	0.02(±0.03)	0.02(±0.04)	0.92	0(±0)	0(±0)	0.96
HexCer(d18:2/20:0)	Glycosylceramides	0.04(±0.08)	0.03(±0.07)	0.38	0.02(±0.03)	0.01(±0.02)	0.08	0(±0)	0(±0.01)	0.15
HexCer(d18:2/22:0)	Glycosylceramides	0.16(±0.26)	0.16(±0.28)	0.63	0.48(±0.23)	0.49(±0.23)	0.91	0(±0)	0(±0)	0.43
HexCer(d18:2/23:0)	Glycosylceramides	0.12(±0.25)	0.11(±0.24)	0.69	0.23(±0.18)	0.21(±0.17)	0.31	0(±0)	0(±0)	0.46
HexCer(d18:2/24:0)	Glycosylceramides	0.2(±0.31)	0.23(±0.36)	0.72	0.79(±0.31)	0.79(±0.3)	0.34	0(±0)	0(±0.01)	0.28
AbsAcid	Hormones	0.36(±1.64)	0.24(±0.94)	0.88	0.1(±0.14)	0.09(±0.14)	0.04	0.36(±0.67)	0.26(±0.57)	<.0001
Cortisol	Hormones	0.09(±0.05)	0.09(±0.05)	0.11	0.26(±0.1)	0.3(±0.13)	0.002	0.01(±0.01)	0.01(±0.01)	0.51
Cortisone	Hormones	0.08(±0.03)	0.08(±0.03)	0.74	0.07(±0.01)	0.07(±0.01)	0.01	0.03(±0.02)	0.03(±0.02)	0.64
DHEAS	Hormones	2.29(±4.09)	2.53(±4.46)	0.98	2.91(±1.87)	3(±2.3)	0.83	0.05(±0.09)	0.06(±0.17)	0.03
3-IAA	Indoles Derivatives	21.03(±33.16)	20.21(±26.83)	0.57	2.34(±1.65)	2.2(±1.3)	0.17	2.45(±2.59)	1.89(±2.72)	0.001
3-IPA	Indoles Derivatives	4.68(±4.22)	4.53(±3.9)	0.80	1(±1.11)	0.64(±0.59)	<.0001	0.01(±0)	0(±0.01)	0.02
Indole	Indoles Derivatives	0.86(±2.52)	0.66(±2.85)	0.50	4.8(±3.05)	5.15(±3.61)	0.28	13.75(±8.08)	14.52(±10.29)	0.04
Ind-SO4	Indoles Derivatives	166.38(±83.12)	160.28(±74.55)	0.07	57.98(±12.48)	57.18(±13.39)	0.58	18.94(±12.86)	17.28(±12.08)	0.98
Hypoxanthine	Nucleobases Related	179.65(±194.95)	198.67(±378.17)	0.98	4.74(±4.12)	5.83(±4.59)	0.0002	37.06(±55.42)	45.09(±88.24)	0.12
Xanthine	Nucleobases Related	230.97(±292.34)	250.58(±416.69)	0.72	0.75(±1.05)	1.24(±3.23)	<.0001	44.15(±50.98)	52.14(±99.93)	0.37
SM (OH) C14:1	Sphingolipids	0.15(±0.16)	0.2(±0.38)	0.59	7.19(±2.09)	6.81(±2.2)	0.05	0(±0.01)	0(±0.02)	0.30
SM (OH) C16:1	Sphingolipids	0.15(±0.2)	0.17(±0.21)	0.88	3.61(±1.01)	3.49(±1.08)	0.18	0(±0)	0(±0.01)	0.46
SM (OH) C22:1	Sphingolipids	0.19(±0.19)	0.21(±0.37)	0.41	10.75(±2.73)	10.28(±2.93)	0.14	0.01(±0.01)	0.01(±0.05)	0.04
SM (OH) C22:2	Sphingolipids	0.05(±0.05)	0.06(±0.1)	0.63	9.48(±2.5)	8.81(±2.66)	0.01	0(±0.01)	0(±0.03)	0.85
SM (OH) C24:1	Sphingolipids	0.06(±0.05)	0.06(±0.07)	0.17	1(±0.29)	0.97(±0.29)	0.19	0(±0)	0(±0)	0.60
SM C16:0	Sphingolipids	3.27(±3.56)	4(±6.1)	0.86	108.23(±21.05)	108.6(±24.38)	0.69	0.06(±0.15)	0.08(±0.5)	0.40
SM C16:1	Sphingolipids	0.11(±0.1)	0.15(±0.23)	0.74	16.01(±3.4)	15.6(±3.75)	0.14	0.01(±0.02)	0.01(±0.07)	0.58
SM C18:0	Sphingolipids	0.78(±1.54)	0.85(±1.49)	0.52	22.02(±5.39)	22.15(±6)	0.62	0.01(±0.02)	0.01(±0.09)	0.34
SM C18:1	Sphingolipids	0.06(±0.08)	0.08(±0.13)	0.50	10.3(±2.6)	10.31(±2.97)	0.89	0(±0.01)	0.01(±0.04)	0.83

Metabolite	Metabolite Class	Stool (no neo.) Mean (±SD)	Stool (ACN) Mean (±SD)	P Value	Blood (no neo.) Mean (±SD)	Blood (ACN) Mean (±SD)	P Value	Urine (no neo.) Mean (±SD)	Urine (ACN) Mean (±SD)	P Value
SM C20:2	Sphingolipids	0.03(±0.04)	0.02(±0.03)	0.14	0.32(±0.1)	0.31(±0.11)	0.18	0(±0)	0(±0)	0.48
SM C22:3	Sphingolipids	0.04(±0.06)	0.04(±0.05)	0.50	0.14(±0.8)	0.04(±0.21)	0.12	0(±0)	0(±0)	0.76
SM C24:0	Sphingolipids	0.4(±0.33)	0.45(±0.56)	0.69	15.08(±3.47)	14.89(±3.63)	0.49	0.02(±0.02)	0.02(±0.08)	0.04
SM C24:1	Sphingolipids	0.37(±0.45)	0.52(±0.99)	0.70	37.93(±8.36)	38.23(±10.43)	0.92	0.02(±0.05)	0.03(±0.18)	0.45
SM C26:0	Sphingolipids	0.03(±0.03)	0.03(±0.04)	0.82	0.13(±0.04)	0.13(±0.04)	0.97	0(±0)	0(±0)	0.55
SM C26:1	Sphingolipids	0.02(±0.03)	0.02(±0.04)	0.39	0.34(±0.1)	0.35(±0.15)	0.61	0(±0)	0(±0)	0.65
H1	Sugars	1223.06(±1059.28)	1305.38(±1097.89)	0.50	4444.09 (±1543.36)	4643.02 (±1464.26)	0.09	609.65(±4002.4)	185.72(±578.59)	0.003
TG(14:0_32:2)	Triacylglycerols	0.28(±0.73)	0.33(±1.06)	0.57	3.85(±3.56)	3.63(±4.37)	0.03	0(±0.01)	0(±0.01)	0.37
TG(14:0_34:0)	Triacylglycerols	0.47(±0.59)	0.52(±0.63)	0.13	7.29(±6.6)	7.35(±8.74)	0.28	0.06(±0.1)	0.06(±0.11)	0.41
TG(14:0_34:1)	Triacylglycerols	1.5(±2.67)	1.48(±3.72)	0.17	44.06(±35.77)	45.2(±53.72)	0.28	0.02(±0.03)	0.02(±0.06)	0.54
TG(14:0_34:2)	Triacylglycerols	0.79(±1.67)	0.84(±4.24)	0.00	22.27(±15.99)	22(±23.54)	0.09	0.01(±0.02)	0.01(±0.04)	0.39
TG(14:0_34:3)	Triacylglycerols	0.09(±0.22)	0.1(±0.38)	0.73	4.69(±3.35)	4.39(±4.44)	0.03	0(±0.01)	0(±0)	0.66
TG(14:0_35:1)	Triacylglycerols	0.11(±0.37)	0.16(±0.45)	0.16	1.18(±1.04)	1.15(±1.43)	0.10	0.01(±0.02)	0.01(±0.02)	0.25
TG(14:0_35:2)	Triacylglycerols	0.05(±0.14)	0.06(±0.15)	0.44	1.21(±0.86)	1.11(±1.14)	0.03	0(±0)	0(±0)	0.40
TG(14:0_36:1)	Triacylglycerols	0.39(±0.82)	0.42(±1.33)	0.27	9.21(±7.07)	9.32(±10.69)	0.23	0(±0.01)	0(±0.01)	0.79
TG(14:0_36:2)	Triacylglycerols	1.39(±2.81)	1.45(±5.9)	0.02	37.67(±24.47)	37.6(±39.56)	0.10	0(±0.01)	0(±0.04)	0.54
TG(14:0_36:3)	Triacylglycerols	1.2(±3)	0.67(±1.57)	0.002	23.94(±15.16)	22.24(±19.6)	0.02	0(±0.01)	0(±0.04)	0.93
TG(14:0_36:4)	Triacylglycerols	1.09(±3.21)	0.47(±1.19)	0.01	7.05(±4.91)	6.1(±5.54)	0.002	0(±0)	0(±0.02)	0.75
TG(14:0_38:4)	Triacylglycerols	0.02(±0.09)	0.02(±0.08)	0.37	0.81(±0.51)	0.79(±0.67)	0.19	0(±0)	0(±0)	0.74
TG(14:0_38:5)	Triacylglycerols	0.01(±0.07)	0.02(±0.11)	0.82	0.7(±0.46)	0.67(±0.59)	0.10	0(±0)	0(±0)	0.19
TG(14:0_39:3)	Triacylglycerols	0.01(±0.04)	0.01(±0.02)	0.30	0.01(±0.02)	0.01(±0.02)	0.38	0(±0)	0(±0)	0.43
TG(16:0_28:1)	Triacylglycerols	0.86(±1.9)	0.73(±2.56)	0.22	6.24(±6.86)	6.04(±10.13)	0.06	0.01(±0.02)	0.01(±0.02)	0.61
TG(16:0_28:2)	Triacylglycerols	0.29(±0.55)	0.25(±0.61)	0.16	1.61(±1.71)	1.47(±2.32)	0.03	0.03(±0.08)	0.03(±0.07)	0.37
TG(16:0_30:2)	Triacylglycerols	0.49(±1.43)	0.41(±1.42)	0.25	4.86(±4.3)	4.51(±5.62)	0.03	0(±0.01)	0(±0.01)	0.39
TG(16:0_32:0)	Triacylglycerols	12.04(±22.71)	9.61(±16.37)	0.86	53.56(±48.54)	56.75(±65.18)	0.72	1.07(±2.4)	1.04(±2.32)	0.73
TG(16:0_32:1)	Triacylglycerols	1.98(±3.29)	2.02(±5.3)	0.14	77.82(±63.61)	83.27(±97.95)	0.57	0.04(±0.05)	0.05(±0.11)	0.58
TG(16:0_32:2)	Triacylglycerols	1.09(±2.03)	1.16(±4.28)	0.04	30.28(±23.01)	30.25(±32.64)	0.16	0.01(±0.02)	0.01(±0.05)	0.68
TG(16:0_32:3)	Triacylglycerols	0.12(±0.24)	0.11(±0.25)	0.66	4.93(±3.95)	4.43(±4.62)	0.03	0(±0)	0(±0.01)	0.99
TG(16:0_33:1)	Triacylglycerols	0.55(±0.57)	0.48(±0.8)	0.03	12.08(±9.67)	12.24(±13.7)	0.42	0.03(±0.04)	0.03(±0.04)	0.34
TG(16:0_33:2)	Triacylglycerols	0.56(±0.71)	0.59(±1.51)	0.49	4.18(±2.95)	4.07(±3.78)	0.19	0(±0.01)	0(±0.01)	0.28
TG(16:0_34:0)	Triacylglycerols	5.15(±5.64)	5(±5.21)	0.62	36.4(±28.75)	40.07(±41.12)	0.75	1.27(±3.07)	1.25(±3.07)	0.71

Metabolite	Metabolite Class	Stool (no neo.) Mean (±SD)	Stool (ACN) Mean (±SD)	P Value	Blood (no neo.) Mean (±SD)	Blood (ACN) Mean (±SD)	P Value	Urine (no neo.) Mean (±SD)	Urine (ACN) Mean (±SD)	P Value
TG(16:0_34:1)	Triacylglycerols	27.58(±56.51)	20.51(±47.37)	0.01	235.59(±167.97)	265.57(±254.44)	0.46	0.14(±0.14)	0.16(±0.45)	0.14
TG(16:0_34:2)	Triacylglycerols	32.14(±103.86)	19.62(±43.04)	0.002	160.87(±102.41)	178.69(±153.97)	0.59	0.06(±0.07)	0.07(±0.36)	0.25
TG(16:0_34:3)	Triacylglycerols	3.69(±14.16)	2.49(±5.2)	0.01	38.57(±23.77)	40.12(±30.12)	0.97	0.01(±0.02)	0.01(±0.09)	0.88
TG(16:0_34:4)	Triacylglycerols	0.2(±0.7)	0.13(±0.32)	0.23	4.4(±3.08)	4.17(±3.43)	0.18	0(±0)	0(±0.01)	0.21
TG(16:0_35:1)	Triacylglycerols	0.76(±1.36)	0.52(±0.95)	0.01	7.8(±5.66)	8.06(±7.99)	0.64	0.02(±0.04)	0.02(±0.04)	0.29
TG(16:0_35:2)	Triacylglycerols	0.7(±1.31)	0.56(±1.32)	0.07	9.1(±5.73)	9.47(±8.04)	0.76	0(±0.01)	0(±0.02)	0.03
TG(16:0_35:3)	Triacylglycerols	0.29(±0.77)	0.21(±0.49)	0.11	3.04(±1.75)	3.02(±2.16)	0.40	0(±0)	0(±0)	0.10
TG(16:0_36:2)	Triacylglycerols	92.66(±249.6)	67.91(±200.19)	0.002	360.63(±197.23)	399.82(±289.98)	0.28	0.06(±0.1)	0.1(±0.65)	0.08
TG(16:0_36:3)	Triacylglycerols	80.59(±231.56)	49.21(±153.84)	<.0001	243.51(±127.21)	254.79(±159.8)	0.66	0.05(±0.11)	0.08(±0.69)	0.12
TG(16:0_36:4)	Triacylglycerols	71.4(±230.52)	34.5(±70.62)	0.001	70.83(±40.87)	68.99(±46.35)	0.41	0.01(±0.04)	0.03(±0.26)	0.19
TG(16:0_36:5)	Triacylglycerols	10(±47.84)	6.24(±17.48)	0.47	10.57(±6.82)	9.55(±7.32)	0.04	0(±0.01)	0(±0.03)	0.59
TG(16:0_36:6)	Triacylglycerols	9.23(±68.54)	5.32(±24.33)	0.63	1.4(±1.02)	1.28(±1.86)	0.003	0.01(±0.03)	0.01(±0.03)	0.22
TG(16:0_37:3)	Triacylglycerols	1.19(±3.33)	0.59(±1.1)	0.01	0.92(±0.5)	0.89(±0.66)	0.12	0(±0)	0(±0)	0.43
TG(16:0_38:1)	Triacylglycerols	0.47(±1.03)	0.59(±2.74)	0.13	2.07(±1.34)	2.1(±1.98)	0.25	0(±0)	0(±0.01)	0.85
TG(16:0_38:2)	Triacylglycerols	1.08(±2.29)	1.25(±5.13)	0.04	4.57(±2.66)	4.86(±4.16)	0.76	0(±0)	0(±0.01)	0.43
TG(16:0_38:3)	Triacylglycerols	0.65(±1.22)	0.66(±2.9)	0.08	5.51(±2.92)	5.84(±4.29)	0.86	0(±0)	0(±0.02)	0.27
TG(16:0_38:4)	Triacylglycerols	0.15(±0.42)	0.14(±0.38)	1.00	7.37(±3.91)	7.88(±5.76)	0.95	0(±0.01)	0.01(±0.02)	0.96
TG(16:0_38:5)	Triacylglycerols	0.08(±0.26)	0.07(±0.18)	0.76	7.72(±4.12)	8.08(±5.43)	0.96	0.04(±0.1)	0.04(±0.11)	0.73
TG(16:0_38:6)	Triacylglycerols	0.04(±0.15)	0.03(±0.11)	0.31	4.53(±2.49)	4.43(±2.63)	0.65	0.01(±0.03)	0.01(±0.03)	0.82
TG(16:0_38:7)	Triacylglycerols	0.09(±0.22)	0.09(±0.19)	0.67	1.14(±0.63)	1.04(±0.63)	0.08	0.02(±0.04)	0.02(±0.04)	0.47
TG(16:0_40:6)	Triacylglycerols	0.02(±0.11)	0.03(±0.1)	0.07	4.74(±2.39)	4.77(±2.47)	0.96	0.01(±0.03)	0.01(±0.03)	0.68
TG(16:0_40:7)	Triacylglycerols	0.03(±0.08)	0.03(±0.08)	1.00	3.98(±2.15)	3.86(±2.09)	0.71	0(±0.01)	0(±0.01)	0.17
TG(16:0_40:8)	Triacylglycerols	0.04(±0.1)	0.07(±0.16)	0.20	1.39(±0.86)	1.28(±0.76)	0.46	0(±0.01)	0(±0.01)	0.53
TG(16:1_28:0)	Triacylglycerols	0.22(±0.37)	0.19(±0.37)	0.22	2.15(±2.1)	2.05(±2.93)	0.02	0.01(±0.03)	0.01(±0.02)	0.32
TG(16:1_30:1)	Triacylglycerols	0.38(±0.62)	0.53(±2.61)	0.33	3.48(±2.91)	3.48(±4.61)	0.048	0.02(±0.03)	0.02(±0.04)	0.97
TG(16:1_32:0)	Triacylglycerols	0.55(±0.72)	0.66(±1.63)	0.71	14.37(±12.6)	16.97(±21.29)	0.70	0.03(±0.04)	0.03(±0.04)	0.75
TG(16:1_32:1)	Triacylglycerols	0.76(±1.24)	1.83(±12.11)	0.76	19.94(±15.67)	22.32(±27.42)	0.59	0.04(±0.05)	0.04(±0.05)	0.27
TG(16:1_32:2)	Triacylglycerols	0.78(±1.15)	1.91(±12.92)	0.82	5.08(±3.45)	5.1(±5.46)	0.07	0.01(±0.02)	0.01(±0.02)	0.89
TG(16:1_33:1)	Triacylglycerols	0.32(±0.35)	0.35(±0.42)	0.62	2.63(±1.84)	2.64(±2.64)	0.37	0.02(±0.03)	0.02(±0.04)	0.06
TG(16:1_34:0)	Triacylglycerols	0.37(±0.65)	0.51(±2.03)	0.23	10.54(±7.71)	12.23(±12.8)	0.56	0(±0.01)	0.01(±0.03)	0.40
TG(16:1_34:1)	Triacylglycerols	2.2(±4.74)	3.88(±20.29)	0.15	75.76(±51.51)	87.85(±85.97)	0.44	0.02(±0.04)	0.03(±0.12)	0.73
TG(16:1_34:2)	Triacylglycerols	2.37(±4.34)	5.37(±38.38)	0.01	37.63(±22.98)	41.32(±34.44)	0.84	0.01(±0.02)	0.01(±0.08)	0.47

Metabolite	Metabolite Class	Stool (no neo.) Mean (±SD)	Stool (ACN) Mean (±SD)	P Value	Blood (no neo.) Mean (±SD)	Blood (ACN) Mean (±SD)	P Value	Urine (no neo.) Mean (±SD)	Urine (ACN) Mean (±SD)	P Value
TG(16:1_34:3)	Triacylglycerols	0.28(±0.95)	0.46(±2.47)	0.47	6.6(±4)	6.65(±4.95)	0.46	0(±0)	0(±0.02)	0.75
TG(16:1_36:1)	Triacylglycerols	0.98(±1.99)	1.53(±8.49)	0.06	9.58(±5.85)	10.78(±9.87)	0.70	0(±0.01)	0(±0.02)	0.98
TG(16:1_36:2)	Triacylglycerols	5.02(±11.29)	7.78(±44.47)	0.001	43.82(±23.67)	48.32(±39.41)	0.80	0(±0.02)	0.01(±0.07)	0.94
TG(16:1_36:3)	Triacylglycerols	3.24(±6.84)	2.66(±7.67)	<.0001	27.11(±13.84)	27.65(±18.16)	0.65	0(±0.01)	0.01(±0.07)	0.54
TG(16:1_36:4)	Triacylglycerols	2.09(±5.25)	1.34(±2.39)	0.02	8.26(±4.6)	7.79(±5.02)	0.13	0(±0)	0(±0.03)	0.18
TG(16:1_36:5)	Triacylglycerols	0.23(±0.74)	0.2(±0.42)	0.36	1.51(±0.9)	1.32(±0.88)	0.01	0(±0)	0(±0.01)	0.99
TG(16:1_38:3)	Triacylglycerols	0.27(±0.8)	0.23(±0.66)	0.69	0.78(±0.37)	0.78(±0.57)	0.12	0(±0)	0(±0)	0.09
TG(16:1_38:4)	Triacylglycerols	0.39(±1.25)	0.22(±0.63)	0.19	1.31(±0.65)	1.35(±0.89)	0.71	0(±0)	0(±0.01)	0.43
TG(16:1_38:5)	Triacylglycerols	0.29(±0.87)	0.11(±0.4)	0.001	1.29(±0.65)	1.29(±0.76)	0.52	0(±0.01)	0(±0.01)	0.87
TG(17:0_32:1)	Triacylglycerols	0.17(±0.28)	0.21(±0.35)	0.59	2.03(±1.68)	1.92(±2.31)	0.13	0.02(±0.05)	0.02(±0.05)	0.72
TG(17:0_34:1)	Triacylglycerols	0.39(±0.68)	0.35(±0.68)	0.61	6.12(±4.31)	6.19(±5.95)	0.56	0(±0.01)	0.01(±0.01)	0.14
TG(17:0_34:2)	Triacylglycerols	0.38(±0.91)	0.29(±0.58)	0.11	3.62(±2.18)	3.64(±2.92)	0.39	0(±0.01)	0(±0.01)	0.44
TG(17:0_34:3)	Triacylglycerols	0.07(±0.31)	0.08(±0.24)	0.48	0.75(±0.43)	0.72(±0.52)	0.08	0(±0)	0(±0.01)	0.77
TG(17:0_36:3)	Triacylglycerols	0.77(±1.83)	0.5(±1.59)	0.001	4.52(±2.37)	4.28(±2.73)	0.11	0(±0)	0(±0.01)	0.44
TG(17:0_36:4)	Triacylglycerols	0.68(±1.78)	0.39(±0.72)	0.04	1.31(±0.71)	1.19(±0.79)	0.01	0(±0)	0(±0)	0.78
TG(17:1_32:1)	Triacylglycerols	0.08(±0.18)	0.11(±0.23)	0.36	1.97(±1.39)	1.98(±2.01)	0.24	0.07(±0.23)	0.07(±0.2)	0.64
TG(17:1_34:1)	Triacylglycerols	0.37(±0.82)	0.28(±1.02)	0.01	7.24(±4.61)	7.67(±6.56)	0.94	0(±0.01)	0(±0.01)	0.90
TG(17:1_34:2)	Triacylglycerols	0.22(±0.56)	0.2(±0.51)	0.50	3.43(±1.93)	3.49(±2.55)	0.54	0(±0.01)	0(±0.01)	0.40
TG(17:1_34:3)	Triacylglycerols	0.07(±0.34)	0.09(±0.39)	0.75	0.64(±0.36)	0.61(±0.42)	0.09	0(±0)	0(±0)	0.91
TG(17:1_36:3)	Triacylglycerols	0.57(±1.43)	0.42(±1.33)	0.04	2.22(±1.1)	2.12(±1.35)	0.10	0(±0)	0(±0.01)	0.59
TG(17:1_36:4)	Triacylglycerols	0.44(±1.15)	0.23(±0.6)	0.001	0.75(±0.4)	0.67(±0.43)	0.01	0(±0)	0(±0)	0.19
TG(17:1_36:5)	Triacylglycerols	0.08(±0.26)	0.06(±0.15)	0.70	0.12(±0.12)	0.1(±0.12)	0.17	0(±0.01)	0(±0.01)	0.63
TG(17:1_38:5)	Triacylglycerols	0.02(±0.06)	0.03(±0.08)	0.01	0.12(±0.09)	0.12(±0.09)	0.77	0(±0)	0(±0)	0.76
TG(17:1_38:6)	Triacylglycerols	0.02(±0.06)	0.03(±0.09)	0.49	0.07(±0.07)	0.07(±0.07)	0.46	0(±0.01)	0(±0.02)	0.13
TG(17:1_38:7)	Triacylglycerols	0(±0.01)	0(±0.01)	0.73	0(±0.01)	0(±0.01)	0.95	0(±0)	0(±0)	1.00
TG(17:2_34:2)	Triacylglycerols	0.08(±0.22)	0.07(±0.16)	0.34	0.24(±0.17)	0.23(±0.2)	0.58	0(±0)	0(±0)	0.43
TG(17:2_34:3)	Triacylglycerols	0.13(±0.29)	0.13(±0.27)	0.98	0.19(±0.19)	0.19(±0.21)	0.69	0(±0)	0(±0)	0.84
TG(17:2_36:2)	Triacylglycerols	0.33(±0.85)	0.29(±0.83)	0.34	0.37(±0.21)	0.36(±0.27)	0.10	0(±0)	0(±0)	0.74
TG(17:2_36:3)	Triacylglycerols	0.34(±0.85)	0.22(±0.55)	0.05	0.24(±0.16)	0.21(±0.18)	0.04	0(±0)	0(±0)	0.50
TG(17:2_36:4)	Triacylglycerols	0.64(±1.37)	0.34(±0.62)	0.001	0.44(±0.27)	0.45(±0.31)	0.96	0(±0.02)	0(±0.02)	0.87
TG(17:2_38:5)	Triacylglycerols	0.08(±0.19)	0.07(±0.18)	0.49	0.12(±0.1)	0.12(±0.11)	0.63	0(±0)	0(±0)	0.10
TG(17:2_38:6)	Triacylglycerols	0.04(±0.12)	0.03(±0.1)	0.21	0.09(±0.1)	0.08(±0.09)	0.36	0(±0)	0(±0)	0.90

Metabolite	Metabolite Class	Stool (no neo.) Mean (±SD)	Stool (ACN) Mean (±SD)	P Value	Blood (no neo.) Mean (±SD)	Blood (ACN) Mean (±SD)	P Value	Urine (no neo.) Mean (±SD)	Urine (ACN) Mean (±SD)	P Value
TG(17:2_38:7)	Triacylglycerols	0.03(±0.09)	0.02(±0.07)	0.28	0.03(±0.04)	0.03(±0.04)	0.75	0(±0.01)	0(±0.01)	0.63
TG(18:0_30:0)	Triacylglycerols	0.79(±2.13)	0.67(±0.94)	0.85	4.88(±5.04)	4.73(±5.87)	0.16	0.06(±0.1)	0.06(±0.09)	0.90
TG(18:0_30:1)	Triacylglycerols	0.43(±1.25)	0.37(±1.07)	0.88	3.15(±2.96)	3.05(±3.92)	0.11	0(±0.01)	0(±0.01)	0.94
TG(18:0_32:0)	Triacylglycerols	1.9(±1.34)	1.89(±0.98)	0.79	9.6(±9.59)	9.4(±10.79)	0.39	0.65(±1.59)	0.64(±1.58)	0.90
TG(18:0_32:1)	Triacylglycerols	0.95(±1.21)	0.99(±1.6)	0.44	10.95(±9.56)	11.67(±13.71)	0.63	0.01(±0.04)	0.01(±0.03)	0.58
TG(18:0_32:2)	Triacylglycerols	0.56(±1.17)	0.62(±1.51)	0.80	3.15(±2.5)	3.04(±3.24)	0.12	0(±0)	0(±0.01)	0.64
TG(18:0_34:2)	Triacylglycerols	7.99(±28.16)	4.59(±10.08)	0.06	17.94(±12.65)	19.12(±17.68)	0.99	0.01(±0.02)	0.01(±0.05)	0.60
TG(18:0_34:3)	Triacylglycerols	1(±3.08)	0.72(±1.94)	0.08	3.81(±2.53)	3.9(±3.25)	0.64	0(±0)	0(±0.01)	0.43
TG(18:0_36:1)	Triacylglycerols	5.26(±17.48)	3.31(±9.28)	0.048	7.77(±7.95)	7.97(±8.78)	0.93	0.03(±0.04)	0.03(±0.05)	0.63
TG(18:0_36:2)	Triacylglycerols	30.79(±107.56)	19.31(±60.32)	0.01	27.6(±19.69)	29.42(±29.19)	0.83	0.01(±0.02)	0.01(±0.06)	0.06
TG(18:0_36:3)	Triacylglycerols	22.62(±76.8)	12.47(±40.16)	0.001	23.09(±14.64)	23.14(±17.39)	0.80	0(±0.01)	0.01(±0.06)	0.77
TG(18:0_36:4)	Triacylglycerols	19.66(±66.67)	8.82(±26.49)	0.004	7.9(±5.4)	7.52(±5.97)	0.27	0(±0.01)	0(±0.02)	0.99
TG(18:0_36:5)	Triacylglycerols	2.83(±11.3)	1.74(±7.06)	0.02	1.38(±0.91)	1.25(±1.13)	0.03	0.01(±0.03)	0.01(±0.02)	0.38
TG(18:0_38:6)	Triacylglycerols	0.02(±0.09)	0.03(±0.08)	0.86	1.36(±0.64)	1.35(±0.67)	0.99	0.03(±0.09)	0.03(±0.07)	0.43
TG(18:0_38:7)	Triacylglycerols	0.06(±0.15)	0.07(±0.14)	0.47	0.28(±0.17)	0.26(±0.15)	0.23	0.01(±0.01)	0.01(±0.01)	0.62
TG(18:1_26:0)	Triacylglycerols	6.58(±11.15)	6.93(±15.03)	0.59	6.23(±6.9)	5.97(±10.41)	0.03	0(±0.02)	0(±0.01)	0.05
TG(18:1_28:1)	Triacylglycerols	1.11(±2.01)	1.1(±2.53)	0.67	6.59(±6.72)	6.25(±10.47)	0.02	0(±0.01)	0(±0.01)	0.79
TG(18:1_30:0)	Triacylglycerols	1.74(±3.49)	1.6(±4.19)	0.45	56.53(±45.75)	57.38(±69.03)	0.21	0.04(±0.06)	0.04(±0.1)	0.78
TG(18:1_30:1)	Triacylglycerols	1.76(±5.05)	1.97(±7.39)	0.57	32.66(±24.27)	31.58(±37.31)	0.02	0.01(±0.02)	0.01(±0.04)	0.85
TG(18:1_30:2)	Triacylglycerols	0.52(±1.16)	0.41(±0.97)	0.30	5.76(±4.63)	5.1(±5.87)	0.003	0(±0.01)	0(±0.01)	0.99
TG(18:1_31:0)	Triacylglycerols	2.38(±2.53)	2.51(±2.91)	0.17	7.47(±5.71)	7.21(±8.24)	0.12	0.13(±0.17)	0.12(±0.14)	0.38
TG(18:1_32:0)	Triacylglycerols	10.84(±19.15)	8.76(±16.94)	0.02	146.45(±102.45)	166.56(±160.61)	0.42	0.82(±2.07)	0.8(±2.2)	0.43
TG(18:1_32:1)	Triacylglycerols	4.07(±7.41)	5.62(±25.14)	0.05	158.91(±99.84)	172.85(±164.84)	0.84	0.02(±0.04)	0.04(±0.21)	0.95
TG(18:1_32:2)	Triacylglycerols	1.95(±3.21)	3.12(±18.98)	0.03	36.32(±21.75)	35.66(±31.81)	0.08	0(±0.01)	0.01(±0.06)	0.73
TG(18:1_32:3)	Triacylglycerols	0.29(±0.51)	0.24(±0.47)	0.24	4.55(±3.33)	3.99(±3.91)	0.005	0(±0)	0(±0)	0.44
TG(18:1_33:0)	Triacylglycerols	0.54(±0.72)	0.65(±1.03)	0.84	8.21(±5.98)	8.39(±8.43)	0.60	0.01(±0.03)	0.01(±0.03)	0.60
TG(18:1_33:1)	Triacylglycerols	0.94(±1.63)	0.84(±1.58)	0.51	20(±12.57)	20.39(±18.21)	0.57	0.01(±0.01)	0.01(±0.03)	0.27
TG(18:1_33:2)	Triacylglycerols	0.81(±0.96)	0.66(±0.86)	0.02	6.38(±3.64)	6.1(±4.67)	0.07	0(±0.01)	0(±0.01)	0.98
TG(18:1_33:3)	Triacylglycerols	0.24(±0.46)	0.23(±0.38)	0.85	0.85(±0.55)	0.74(±0.55)	0.02	0(±0)	0(±0)	0.91
TG(18:1_34:1)	Triacylglycerols	146.98(±399.34)	112.63(±325.09)	0.003	604.84(±328.16)	670.04(±453.26)	0.21	0.1(±0.17)	0.16(±1.07)	0.27
TG(18:1_34:2)	Triacylglycerols	78.36(±218.07)	57.92(±177.92)	0.0002	285.9(±146.22)	304.42(±197.22)	0.63	0.05(±0.12)	0.08(±0.65)	0.27
TG(18:1_34:3)	Triacylglycerols	8.98(±20.11)	6.72(±13.45)	0.002	45.84(±25.44)	45.35(±30.93)	0.39	0.01(±0.02)	0.01(±0.09)	0.01

Metabolite	Metabolite Class	Stool (no neo.) Mean (±SD)	Stool (ACN) Mean (±SD)	P Value	Blood (no neo.) Mean (±SD)	Blood (ACN) Mean (±SD)	P Value	Urine (no neo.) Mean (±SD)	Urine (ACN) Mean (±SD)	P Value
TG(18:1_34:4)	Triacylglycerols	0.63(±1.35)	0.43(±0.82)	0.02	4.79(±2.85)	4.36(±3.21)	0.03	0(±0)	0(±0.01)	0.04
TG(18:1_35:2)	Triacylglycerols	2.66(±5.86)	2.26(±6.42)	0.06	9.43(±5.09)	9.48(±7.19)	0.33	0(±0)	0(±0.02)	0.38
TG(18:1_35:3)	Triacylglycerols	0.69(±1.43)	0.52(±1.29)	0.09	2.51(±1.31)	2.38(±1.59)	0.06	0(±0)	0(±0)	0.69
TG(18:1_36:0)	Triacylglycerols	7(±22.86)	4.94(±14.59)	0.01	8.53(±6.44)	8.96(±9.04)	0.89	0.31(±0.81)	0.3(±0.81)	0.57
TG(18:1_36:1)	Triacylglycerols	105.71(±346.19)	78.89(±240.22)	0.005	66.16(±45.08)	71.06(±72.26)	0.89	0.02(±0.03)	0.02(±0.13)	0.85
TG(18:1_36:2)	Triacylglycerols	520.27(±1415.17)	421.48(±1210.48)	0.005	209.3(±134.74)	218.81(±200.21)	0.90	0.04(±0.08)	0.06(±0.36)	0.78
TG(18:1_36:3)	Triacylglycerols	256.32(±655.34)	175.72(±560.03)	0.0001	129.23(±87.72)	123.9(±100.56)	0.37	0.03(±0.06)	0.05(±0.33)	0.98
TG(18:1_36:4)	Triacylglycerols	145.73(±434.32)	78.17(±225.68)	0.0002	43.76(±33.13)	38.97(±34.04)	0.04	0.01(±0.03)	0.02(±0.13)	0.90
TG(18:1_36:5)	Triacylglycerols	15.95(±43.24)	9.45(±20.99)	0.01	8.66(±6.54)	7.45(±7.58)	0.002	0.03(±0.09)	0.03(±0.09)	0.74
TG(18:1_36:6)	Triacylglycerols	9.1(±49.56)	7.26(±32.69)	0.66	1.28(±0.99)	1.19(±2.38)	0.0002	0.07(±0.25)	0.07(±0.21)	0.51
TG(18:1_38:5)	Triacylglycerols	0.07(±0.19)	0.08(±0.22)	0.49	8.8(±3.92)	8.76(±4.23)	0.77	0(±0)	0(±0.02)	0.82
TG(18:1_38:6)	Triacylglycerols	0.07(±0.18)	0.08(±0.19)	0.59	4.64(±2.25)	4.39(±2.13)	0.32	0(±0)	0(±0.01)	0.04
TG(18:1_38:7)	Triacylglycerols	0.33(±0.72)	0.23(±0.46)	0.20	0.95(±0.47)	0.84(±0.43)	0.01	0(±0)	0(±0)	0.38
TG(18:2_28:0)	Triacylglycerols	0.55(±1.9)	0.4(±1.66)	0.09	5.8(±5.42)	5.15(±6.69)	0.01	0(±0.01)	0(±0)	0.39
TG(18:2_30:0)	Triacylglycerols	0.76(±2.13)	0.49(±1.14)	0.07	18.71(±14.34)	17.46(±18.39)	0.04	0(±0.01)	0.01(±0.04)	0.85
TG(18:2_30:1)	Triacylglycerols	0.49(±1.21)	0.36(±1.04)	0.04	10(±7.32)	8.98(±9.21)	0.01	0(±0.01)	0(±0.02)	0.22
TG(18:2_31:0)	Triacylglycerols	1.04(±0.48)	1(±0.43)	0.64	2.71(±1.25)	2.61(±1.42)	0.23	0.12(±0.12)	0.1(±0.11)	0.09
TG(18:2_32:0)	Triacylglycerols	12.03(±40.17)	6.72(±14.59)	0.003	50.39(±32.61)	53.12(±43.78)	0.87	0.09(±0.21)	0.1(±0.3)	0.23
TG(18:2_32:1)	Triacylglycerols	2.06(±4.55)	1.46(±3.14)	0.003	51.31(±29.58)	51.67(±39.73)	0.37	0.01(±0.02)	0.01(±0.1)	0.22
TG(18:2_32:2)	Triacylglycerols	2.02(±5.58)	1.02(±2.23)	0.01	11.71(±7.5)	10.53(±8.35)	0.01	0(±0.01)	0(±0.03)	0.91
TG(18:2_33:0)	Triacylglycerols	0.31(±0.69)	0.22(±0.45)	0.07	3.08(±2.01)	2.94(±2.42)	0.14	0(±0)	0(±0.01)	0.38
TG(18:2_33:1)	Triacylglycerols	0.52(±1.01)	0.43(±0.87)	0.21	6.88(±3.98)	6.62(±4.77)	0.16	0(±0.01)	0(±0.01)	0.28
TG(18:2_33:2)	Triacylglycerols	0.85(±1.49)	0.56(±0.88)	0.002	2.15(±1.25)	1.94(±1.36)	0.01	0(±0)	0(±0.01)	0.42
TG(18:2_34:0)	Triacylglycerols	11.32(±35.15)	6.54(±16.91)	0.002	31.82(±18.48)	33.4(±24.12)	0.75	0.13(±0.34)	0.13(±0.38)	0.77
TG(18:2_34:1)	Triacylglycerols	71.33(±203.86)	42.57(±124.53)	0.0001	228.49(±119.81)	238.22(±149.26)	0.66	0.04(±0.1)	0.07(±0.6)	0.01
TG(18:2_34:2)	Triacylglycerols	107.26(±346.85)	51.78(±112.55)	0.001	101.09(±58.53)	99.74(±65.35)	0.70	0.02(±0.07)	0.05(±0.41)	0.047
TG(18:2_34:3)	Triacylglycerols	10.08(±34.19)	6.05(±12.91)	0.02	15.14(±9.43)	13.81(±9.81)	0.07	0(±0.01)	0.01(±0.05)	0.63
TG(18:2_34:4)	Triacylglycerols	1.01(±4.13)	0.63(±1.7)	0.45	1.62(±1.03)	1.4(±1.05)	0.004	0(±0)	0(±0)	0.45
TG(18:2_35:1)	Triacylglycerols	2.11(±4.4)	1.41(±2.26)	0.002	5.06(±2.73)	4.82(±3.27)	0.10	0(±0)	0(±0.01)	0.38
TG(18:2_35:2)	Triacylglycerols	1.86(±4.55)	1.05(±2.02)	<.0001	3.19(±1.71)	2.98(±1.94)	0.06	0(±0)	0(±0.01)	0.86
TG(18:2_35:3)	Triacylglycerols	0.85(±2.13)	0.46(±1.01)	0.003	0.91(±0.51)	0.82(±0.55)	0.02	0(±0)	0(±0.01)	0.15
TG(18:2_36:0)	Triacylglycerols	3.49(±10.77)	1.97(±6.15)	0.001	3.37(±2.21)	3.35(±2.53)	0.70	0.03(±0.09)	0.03(±0.09)	0.73

Metabolite	Metabolite Class	Stool (no neo.) Mean (±SD)	Stool (ACN) Mean (±SD)	P Value	Blood (no neo.) Mean (±SD)	Blood (ACN) Mean (±SD)	P Value	Urine (no neo.) Mean (±SD)	Urine (ACN) Mean (±SD)	P Value
TG(18:2_36:1)	Triacylglycerols	32.41(±97.91)	19.85(±62.43)	0.0003	26.65(±16.66)	26.61(±20.22)	0.73	0(±0.02)	0.01(±0.08)	0.48
TG(18:2_36:2)	Triacylglycerols	162.61(±458.3)	102.55(±317.28)	<.0001	68.94(±46.8)	66.08(±51.72)	0.41	0.02(±0.04)	0.03(±0.22)	0.21
TG(18:2_36:3)	Triacylglycerols	245.58(±733.18)	127.2(±380.62)	0.0001	42.01(±36.26)	36.11(±30.03)	0.09	0.01(±0.03)	0.02(±0.18)	0.92
TG(18:2_36:4)	Triacylglycerols	226.35(±628.09)	105.47(±284.57)	0.002	15.98(±16.17)	12.64(±13.28)	0.01	0.01(±0.02)	0.01(±0.07)	0.01
TG(18:2_36:5)	Triacylglycerols	27.53(±116.53)	15.18(±41.54)	0.35	3.28(±3.27)	2.62(±3.62)	0.0004	0(±0)	0(±0.02)	0.57
TG(18:2_38:4)	Triacylglycerols	0.43(±0.97)	0.31(±0.55)	0.16	2.66(±1.3)	2.58(±1.4)	0.37	0(±0)	0(±0.01)	0.03
TG(18:2_38:5)	Triacylglycerols	0.07(±0.36)	0.07(±0.21)	0.56	3.43(±1.65)	3.21(±1.52)	0.30	0(±0)	0(±0.01)	0.45
TG(18:2_38:6)	Triacylglycerols	0.03(±0.17)	0.04(±0.14)	0.46	1.88(±1.08)	1.68(±0.89)	0.08	0(±0)	0(±0.01)	0.99
TG(18:3_30:0)	Triacylglycerols	0.04(±0.14)	0.04(±0.14)	0.72	2.86(±2.47)	2.58(±3.4)	0.01	0(±0.01)	0(±0.01)	0.25
TG(18:3_32:0)	Triacylglycerols	0.96(±4.61)	0.62(±1.57)	0.13	6.58(±4.92)	6.39(±6.26)	0.22	0(±0.01)	0.01(±0.02)	0.85
TG(18:3_32:1)	Triacylglycerols	0.19(±0.53)	0.16(±0.37)	0.95	7.25(±5.05)	6.66(±5.96)	0.03	0.01(±0.02)	0.01(±0.02)	0.35
TG(18:3_33:2)	Triacylglycerols	0.11(±0.33)	0.13(±0.38)	0.43	0.24(±0.2)	0.19(±0.21)	0.004	0(±0)	0(±0)	0.68
TG(18:3_34:0)	Triacylglycerols	1.06(±3.31)	0.75(±2.13)	0.74	3.93(±2.53)	3.88(±3.23)	0.35	0.01(±0.02)	0.01(±0.02)	0.80
TG(18:3_34:1)	Triacylglycerols	8.25(±23.07)	5.37(±12.2)	0.02	29.43(±17.88)	28.2(±21.05)	0.14	0(±0.01)	0.01(±0.06)	0.44
TG(18:3_34:2)	Triacylglycerols	10.01(±49.27)	6.27(±17.29)	0.47	13.88(±8.45)	12.64(±9.7)	0.02	0(±0.01)	0(±0.04)	0.69
TG(18:3_34:3)	Triacylglycerols	14.03(±94.55)	8.93(±40.24)	0.86	2.28(±1.64)	2.12(±3.64)	0.002	0(±0)	0(±0.01)	0.85
TG(18:3_35:2)	Triacylglycerols	0.27(±1.28)	0.16(±0.46)	0.21	0.55(±0.36)	0.46(±0.41)	0.001	0(±0)	0(±0)	0.88
TG(18:3_36:1)	Triacylglycerols	3.6(±10.18)	2.27(±6.54)	0.003	3.69(±2.72)	3.43(±3.31)	0.04	0(±0)	0(±0.01)	0.30
TG(18:3_36:2)	Triacylglycerols	15.22(±41.78)	9.66(±25.4)	0.001	12.96(±9.79)	11.63(±11.42)	0.01	0(±0)	0(±0.03)	0.50
TG(18:3_36:3)	Triacylglycerols	22.53(±76.83)	13.58(±41.03)	0.01	7.39(±6.11)	6.42(±8.07)	0.001	0(±0.01)	0(±0.03)	0.74
TG(18:3_36:4)	Triacylglycerols	32.9(±155.82)	21.99(±80.13)	0.42	2.91(±2.89)	2.69(±6.61)	<.0001	0(±0)	0(±0.01)	0.77
TG(18:3_38:5)	Triacylglycerols	0.08(±0.6)	0.06(±0.25)	0.32	0.51(±0.27)	0.43(±0.27)	0.003	0(±0)	0(±0)	0.37
TG(18:3_38:6)	Triacylglycerols	0.07(±0.47)	0.04(±0.26)	0.51	0.27(±0.18)	0.23(±0.17)	0.02	0(±0)	0(±0)	0.57
TG(20:0_32:3)	Triacylglycerols	0.17(±0.62)	0.12(±0.37)	0.52	0.59(±0.32)	0.63(±0.37)	0.64	0(±0)	0(±0)	0.43
TG(20:0_32:4)	Triacylglycerols	0.58(±1.91)	0.23(±0.58)	<.0001	0.51(±0.3)	0.5(±0.32)	0.42	0(±0)	0(±0)	0.92
TG(20:0_34:1)	Triacylglycerols	0.98(±1.59)	0.96(±3.26)	0.01	2.13(±1.13)	2.19(±1.79)	0.35	0.01(±0.02)	0.01(±0.02)	0.64
TG(20:1_24:3)	Triacylglycerols	0.04(±0.09)	0.07(±0.13)	0.06	0.3(±0.1)	0.29(±0.11)	0.90	0(±0.01)	0(±0.01)	0.78
TG(20:1_26:1)	Triacylglycerols	0.03(±0.07)	0.03(±0.06)	0.31	0.08(±0.07)	0.08(±0.06)	0.49	0(±0)	0(±0)	0.19
TG(20:1_30:1)	Triacylglycerols	0.04(±0.14)	0.06(±0.16)	0.13	0.23(±0.18)	0.22(±0.22)	0.07	0(±0)	0(±0)	0.59
TG(20:1_31:0)	Triacylglycerols	1.58(±0.8)	1.71(±0.99)	0.18	2.14(±1.1)	2.2(±1.6)	0.95	0.16(±0.16)	0.15(±0.14)	0.30
TG(20:1_32:1)	Triacylglycerols	0.03(±0.12)	0.11(±0.72)	0.40	1.55(±0.96)	1.61(±1.45)	0.36	0.03(±0.09)	0.03(±0.08)	0.50
TG(20:1_32:2)	Triacylglycerols	0.06(±0.2)	0.14(±1.08)	0.86	0.49(±0.28)	0.48(±0.35)	0.15	0(±0)	0(±0)	0.76

Metabolite	Metabolite Class	Stool (no neo.) Mean (±SD)	Stool (ACN) Mean (±SD)	P Value	Blood (no neo.) Mean (±SD)	Blood (ACN) Mean (±SD)	P Value	Urine (no neo.) Mean (±SD)	Urine (ACN) Mean (±SD)	P Value
TG(20:1_32:3)	Triacylglycerols	0.05(±0.15)	0.04(±0.1)	0.41	0.14(±0.12)	0.12(±0.13)	0.04	0(±0)	0(±0)	0.84
TG(20:1_34:0)	Triacylglycerols	0.1(±0.25)	0.18(±0.44)	0.13	0.7(±0.46)	0.76(±0.68)	0.98	0.02(±0.04)	0.02(±0.04)	0.48
TG(20:1_34:1)	Triacylglycerols	0.77(±1.83)	1.04(±3.7)	0.26	4.05(±2.51)	4.45(±3.97)	0.70	0(±0)	0(±0.01)	0.67
TG(20:1_34:2)	Triacylglycerols	0.74(±1.36)	1.02(±4.46)	0.28	2.32(±1.25)	2.34(±1.7)	0.43	0(±0)	0(±0)	0.04
TG(20:1_34:3)	Triacylglycerols	0.21(±0.55)	0.16(±0.51)	0.05	0.45(±0.28)	0.43(±0.31)	0.08	0(±0)	0(±0)	0.44
TG(20:2_32:0)	Triacylglycerols	0.05(±0.11)	0.05(±0.12)	0.74	0.87(±0.59)	0.99(±0.98)	0.81	0(±0.01)	0(±0.01)	0.14
TG(20:2_32:1)	Triacylglycerols	0.07(±0.31)	0.07(±0.25)	0.40	1.41(±0.78)	1.52(±1.3)	0.58	0(±0.01)	0(±0.02)	0.87
TG(20:2_34:1)	Triacylglycerols	0.09(±0.33)	0.1(±0.35)	0.66	3.88(±2.02)	4.24(±3.32)	0.91	0(±0)	0(±0.01)	0.09
TG(20:2_34:2)	Triacylglycerols	0.34(±0.88)	0.24(±0.72)	0.01	1.98(±0.96)	2.03(±1.38)	0.33	0(±0)	0(±0)	0.88
TG(20:2_34:3)	Triacylglycerols	0.36(±0.89)	0.24(±0.51)	0.06	0.39(±0.22)	0.37(±0.27)	0.24	0(±0)	0(±0)	0.13
TG(20:2_34:4)	Triacylglycerols	0.3(±0.84)	0.19(±0.52)	0.33	0.06(±0.12)	0.05(±0.1)	0.32	0(±0)	0(±0.01)	0.48
TG(20:2_36:5)	Triacylglycerols	0.04(±0.13)	0.04(±0.14)	0.67	0.05(±0.06)	0.04(±0.06)	0.45	0(±0)	0(±0.01)	0.64
TG(20:3_32:0)	Triacylglycerols	0.03(±0.08)	0.02(±0.08)	0.18	2.21(±1.44)	2.48(±2.35)	0.93	0(±0)	0(±0.01)	0.06
TG(20:3_32:1)	Triacylglycerols	0.13(±0.45)	0.09(±0.22)	0.97	2.19(±1.34)	2.31(±2.06)	0.47	0(±0)	0(±0)	0.59
TG(20:3_32:2)	Triacylglycerols	0.18(±1.04)	0.25(±1.16)	0.34	0.55(±0.33)	0.51(±0.4)	0.06	0(±0)	0(±0)	0.41
TG(20:3_34:0)	Triacylglycerols	0.02(±0.09)	0.01(±0.08)	0.68	1.02(±0.56)	1.1(±0.87)	0.89	0(±0.01)	0(±0.01)	0.92
TG(20:3_34:1)	Triacylglycerols	0.09(±0.3)	0.11(±0.3)	0.43	8.14(±4.37)	8.71(±6.47)	0.99	0(±0)	0(±0.02)	0.51
TG(20:3_34:2)	Triacylglycerols	0.31(±0.9)	0.2(±0.49)	0.51	4.2(±2.05)	4.24(±2.66)	0.44	0(±0.01)	0(±0.02)	0.17
TG(20:3_34:3)	Triacylglycerols	0.34(±1.03)	0.18(±0.43)	0.13	0.84(±0.41)	0.78(±0.44)	0.05	0(±0)	0(±0)	0.30
TG(20:3_36:3)	Triacylglycerols	0.07(±0.18)	0.07(±0.2)	0.41	1.96(±0.9)	1.9(±1)	0.25	0(±0)	0(±0.01)	0.65
TG(20:3_36:4)	Triacylglycerols	0.05(±0.18)	0.06(±0.21)	0.46	0.83(±0.39)	0.76(±0.41)	0.05	0(±0)	0(±0)	0.72
TG(20:3_36:5)	Triacylglycerols	0.03(±0.13)	0.01(±0.06)	0.17	0.16(±0.11)	0.13(±0.12)	0.01	0(±0)	0(±0)	1.00
TG(20:4_30:0)	Triacylglycerols	0.03(±0.11)	0.04(±0.13)	0.95	1.83(±1.54)	1.8(±2)	0.16	0(±0)	0(±0)	0.40
TG(20:4_32:0)	Triacylglycerols	0.02(±0.07)	0.03(±0.1)	0.44	5.04(±3.72)	5.56(±5.16)	0.71	0(±0)	0(±0)	0.14
TG(20:4_32:1)	Triacylglycerols	0.05(±0.17)	0.09(±0.23)	0.10	4.65(±3.31)	4.9(±4.48)	0.68	0(±0)	0(±0.01)	0.76
TG(20:4_32:2)	Triacylglycerols	0.08(±0.2)	0.11(±0.24)	0.37	1.18(±0.7)	1.14(±0.87)	0.16	0(±0)	0(±0)	1.00
TG(20:4_33:2)	Triacylglycerols	0.06(±0.12)	0.07(±0.14)	0.44	0.13(±0.09)	0.12(±0.1)	0.15	0(±0)	0(±0)	0.73
TG(20:4_34:0)	Triacylglycerols	0.02(±0.09)	0.03(±0.14)	0.37	3.21(±1.84)	3.55(±2.49)	0.32	0(±0)	0(±0.01)	0.42
TG(20:4_34:1)	Triacylglycerols	0.08(±0.23)	0.13(±0.38)	0.76	19.33(±10.66)	20.87(±14.39)	0.52	0(±0.01)	0(±0.04)	0.97
TG(20:4_34:2)	Triacylglycerols	0.1(±0.26)	0.11(±0.26)	0.72	9.56(±5.11)	9.97(±6.42)	0.74	0(±0.01)	0(±0.03)	0.13
TG(20:4_34:3)	Triacylglycerols	0.09(±0.26)	0.05(±0.15)	0.07	1.55(±0.85)	1.47(±0.88)	0.24	0(±0)	0(±0)	0.76
TG(20:4_35:3)	Triacylglycerols	0.02(±0.06)	0.03(±0.07)	0.23	0.04(±0.05)	0.03(±0.05)	0.02	0(±0)	0(±0)	0.30

Metabolite	Metabolite Class	Stool (no neo.) Mean (±SD)	Stool (ACN) Mean (±SD)	P Value	Blood (no neo.) Mean (±SD)	Blood (ACN) Mean (±SD)	P Value	Urine (no neo.) Mean (±SD)	Urine (ACN) Mean (±SD)	P Value
TG(20:4_36:2)	Triacylglycerols	0.08(±0.26)	0.09(±0.38)	0.82	10.2(±4.48)	10.42(±5.01)	0.74	0(±0)	0(±0.02)	0.14
TG(20:4_36:3)	Triacylglycerols	0.07(±0.17)	0.07(±0.19)	0.28	5.44(±2.72)	5.2(±2.55)	0.53	0(±0)	0(±0.02)	0.73
TG(20:4_36:4)	Triacylglycerols	0.03(±0.1)	0.03(±0.1)	0.61	2.09(±1.22)	1.94(±1.09)	0.24	0(±0)	0(±0.01)	0.78
TG(20:4_36:5)	Triacylglycerols	0.02(±0.12)	0.02(±0.07)	0.52	0.58(±0.39)	0.49(±0.34)	0.03	0(±0)	0(±0)	0.19
TG(20:5_34:0)	Triacylglycerols	0.03(±0.1)	0.03(±0.09)	0.37	0.78(±0.55)	0.72(±0.47)	0.29	0(±0.01)	0(±0.01)	0.83
TG(20:5_34:1)	Triacylglycerols	0.12(±0.29)	0.17(±0.33)	0.05	4.87(±3.25)	4.25(±2.59)	0.09	0.01(±0.02)	0.01(±0.02)	0.38
TG(20:5_34:2)	Triacylglycerols	0.12(±0.27)	0.12(±0.26)	0.51	2.63(±1.69)	2.2(±1.2)	0.02	0.01(±0.03)	0.01(±0.02)	0.52
TG(20:5_36:2)	Triacylglycerols	0.22(±0.53)	0.21(±0.89)	0.03	2.77(±1.86)	2.27(±1.35)	0.002	0(±0.01)	0(±0.01)	0.41
TG(20:5_36:3)	Triacylglycerols	0.33(±0.84)	0.23(±0.44)	0.63	1.77(±1.19)	1.4(±0.78)	0.001	0(±0)	0(±0.01)	0.43
TG(22:0_32:4)	Triacylglycerols	0.03(±0.1)	0.02(±0.05)	0.07	0.02(±0.02)	0.01(±0.02)	0.71	0(±0)	0(±0)	0.70
TG(22:1_32:5)	Triacylglycerols	0.11(±0.26)	0.07(±0.14)	0.17	0.03(±0.04)	0.02(±0.04)	0.09	0(±0)	0(±0.01)	0.42
TG(22:2_32:4)	Triacylglycerols	0.72(±2.03)	0.38(±0.93)	0.01	0.09(±0.11)	0.07(±0.1)	0.01	0.01(±0.02)	0.01(±0.02)	0.67
TG(22:3_30:2)	Triacylglycerols	0.03(±0.08)	0.04(±0.1)	0.32	0.02(±0.04)	0.02(±0.03)	0.70	0(±0)	0(±0)	0.15
TG(22:4_32:0)	Triacylglycerols	0.01(±0.06)	0.01(±0.06)	0.62	0.53(±0.37)	0.61(±0.52)	0.36	0(±0)	0(±0)	0.30
TG(22:4_32:2)	Triacylglycerols	0.03(±0.08)	0.03(±0.07)	0.95	0.05(±0.05)	0.05(±0.05)	0.71	0(±0)	0(±0)	0.05
TG(22:4_34:2)	Triacylglycerols	0.06(±0.19)	0.04(±0.14)	0.62	1.18(±0.58)	1.28(±0.72)	0.23	0(±0)	0(±0.01)	0.10
TG(22:5_32:0)	Triacylglycerols	0.06(±0.15)	0.05(±0.13)	0.71	1.48(±0.91)	1.64(±1.13)	0.25	0.03(±0.07)	0.03(±0.07)	0.57
TG(22:5_32:1)	Triacylglycerols	0.04(±0.12)	0.04(±0.13)	0.61	1.45(±0.79)	1.49(±1.01)	0.71	0(±0.02)	0(±0.02)	0.76
TG(22:5_34:1)	Triacylglycerols	0.13(±0.42)	0.11(±0.48)	0.81	7.56(±3.76)	7.8(±4.05)	0.58	0.01(±0.02)	0.01(±0.02)	0.33
TG(22:5_34:2)	Triacylglycerols	0.08(±0.25)	0.13(±0.31)	0.04	3.51(±1.64)	3.51(±1.64)	0.98	0(±0)	0(±0.01)	0.67
TG(22:5_34:3)	Triacylglycerols	0.03(±0.1)	0.06(±0.16)	0.26	0.61(±0.29)	0.55(±0.28)	0.13	0(±0)	0(±0)	0.75
TG(22:6_32:0)	Triacylglycerols	0.04(±0.14)	0.04(±0.12)	0.96	2.71(±1.9)	2.9(±2.25)	0.57	0.01(±0.04)	0.02(±0.04)	0.26
TG(22:6_32:1)	Triacylglycerols	0.04(±0.14)	0.05(±0.15)	0.38	2.76(±1.88)	2.66(±1.98)	0.47	0.02(±0.08)	0.02(±0.07)	0.49
TG(22:6_34:1)	Triacylglycerols	0.05(±0.15)	0.07(±0.22)	0.79	11.57(±7.77)	11.48(±7.42)	0.84	0(±0)	0(±0.02)	0.31
TG(22:6_34:2)	Triacylglycerols	0.07(±0.19)	0.05(±0.23)	0.07	6.54(±4.72)	6.08(±3.77)	0.77	0(±0)	0(±0.01)	0.64
TG(22:6_34:3)	Triacylglycerols	0.02(±0.09)	0.02(±0.09)	0.84	0.92(±0.7)	0.78(±0.5)	0.13	0(±0)	0(±0)	0.47
Choline	Vitamins & Cofactors	21.46(±21.13)	24.51(±23.32)	0.06	6.77(±1.96)	7.06(±2.23)	0.17	1.49(±0.63)	1.53(±0.74)	0.83

Significant different concentrations of metabolites between study participants with and without advanced colorectal neoplasms are marked in grey (Mann-Whitney U test); **Abbreviations**: 3-IAA, 3-Indoleacetic acid; 3-IPA, 3-Indolepropionic acid; AA, arachidonic acid; AABA, alpha-Aminobutyric acid; ACN, advanced colorectal neoplasms; ADMA, Asymmetric dimethylarginine; Ala, Alanine; alpha-AAA, alpha Aminoadipic acid; Asn, asparagine; BABA, beta-Aminobutyric acid; C8, Octanoylcarnitine; C10, Decanoylcarnitine; C10:1, Decenoylcarnitine; C12, Dodecanoylcarnitine; C16, Hexadecanoylcarnitine; CE, Cholesteryl ester; Cer, Ceramide; Cys, Cysteine; DCA, Deoxycholic acid; DG, Diglycerides; DHA, Docosahexaenoic acid; FA, Fatty acid; FA (16:0), Palmitic acid; FA (18:1), Octadecenoic acid; FA (20:1), Eicosenoic acid; FA(20:2), Eicosadienoic acid; FA (20:3); Eicosatrienoic acid; GABA,

gamma-Aminobutyric acid; Gln, Glutamine; Glu, Glutamate; HArg, Homoarginine; HexCer, Hexosylceramide; Hex2Cer, Dihexosylceramide; His, Histidine; lysoPC, Lysophosphatidylcholine; Met-So, Methionine sulfoxide; no neo., no neoplasms; PC, Phosphatidylcholine; SDMA, Symmetric dimethylarginine; Ser, Serine; SM, Sphingomyelin; TG, Triglyceride; Thr, Threonine; Trp, Tryptophan; Tyr, Tyrosine.
